# Isometric *versus* isotonic exercise in individuals with rotator cuff tendinopathy—Effects on shoulder pain, functioning, muscle strength, and electromyographic activity: A protocol for randomized clinical trial

**DOI:** 10.1371/journal.pone.0293457

**Published:** 2023-11-13

**Authors:** Bianca Rodrigues da Silva Barros, Denise Dal’Ava Augusto, João Felipe de Medeiros Neto, Lori Ann Michener, Rodrigo Scattone Silva, Catarina de Oliveira Sousa

**Affiliations:** 1 Department of Physical Therapy, Postgraduate Program of Physical Therapy, Federal University of Rio Grande do Norte, Natal, State of Rio Grande do Norte, Brazil; 2 Department of Surgery, Federal University of Rio Grande do Norte, Natal, State of Rio Grande do Norte, Brazil; 3 Division of Biokinesiology and Physical Therapy, University of Southern California, Los Angeles, California, United States of America; 4 Faculty of Health Sciences of Trairi, Postgraduate Program in Rehabilitation Sciences, Federal University of Rio Grande do Norte, Santa Cruz, State of Rio Grande do Norte, Brazil; Fondazione Policlinico Universitario Gemelli IRCCS, ITALY

## Abstract

**Introduction:**

Rotator cuff tendinopathy is a common shoulder disorder in which the primary treatment is resistance exercises. Isometric exercises are being studied for lower limb tendinopathies but not for rotator cuff tendinopathy. This protocol for a randomized clinical trial aims to compare the effects of two types of exercise (isometric and isotonic) on shoulder pain, functioning, muscle strength, and electromyographic activity in individuals with rotator cuff tendinopathy.

**Methods:**

Forty-six individuals (18 to 60 years old) with shoulder pain for more than three months and unilateral supraspinatus and/or infraspinatus tendinopathy will participate in this trial. Individuals will be randomized into two exercise groups: isometric or isotonic. The following outcomes will be evaluated before and after the first session and after six weeks of intervention: shoulder pain and functioning; isometric strength of shoulder elevation and lateral and medial rotation; and electromyographic activity of medial deltoid, infraspinatus, serratus anterior, and lower trapezius. Groups will perform stretching and strengthening of periscapular muscles. The isometric group will perform three sets of 32 s, at 70% of maximal isometric strength. The isotonic group will perform concentric and eccentric exercises (2 s for each phase) in three sets of eight repetitions at a load of eight repetition maximum. The total time under tension of 96 s will be equal for both groups, and load will be adjusted in weeks three and five of the protocol. Treatment effect between groups will be analyzed using linear mixed model.

**Trial registration:**

**Trial registration number:** Universal Trial Number (UTN) code U1111-1284-7528 and Brazilian Clinical Trials Registry platform–RBR-3pvdvfk.

## Introduction

Rotator cuff (RC) tendinopathy, also known as subacromial impingement [1,2], is a common shoulder disorder with prevalence of up to 27% in individuals under 70 years old [3,4]. Intrinsic and extrinsic factors may lead to development of the disorder [2], including tendon mechanical properties (composition and vascularization) and scapular and glenohumeral movement alteration, contributing to internal and external impingement [2,5].

Pain due to RC tendinopathy negatively impacts activities of daily living [6,7], work, and leisure [3] and leads to neuromuscular alteration [8]. Compared with surgical intervention, exercises provide similar results on RC tendinopathy and partial or total RC rupture [1]. Furthermore, concentric and eccentric isotonic exercises have been shown to be equally effective for improving shoulder pain, functioning [9,10], and muscular strength [11].

Isometric contractions have been shown to increase pain tolerance in young healthy participants [12] and older people [13], as well as decrease temporal summation of pain in healthy participants 15 minutes after the exercise [12], which demonstrate that central mechanisms are also involved in exercise induced hypoalgesia. Some studies reported pain improvement in patients with patellar tendinopathy after isometric exercises. Rio et al. [14] observed isometric exercises improved pain (immediately after the exercise and after four weeks) and quadriceps function (after four weeks) compared with isotonic exercises in patients with patellar tendinopathy. Other authors also compared the effects of these exercises after 45 minutes [15] and four weeks [15,16] in patients with patellar tendinopathy and observed improved strength and reduced cortical inhibition and pain. For the shoulder complex, however, the use of isometric exercise with load progression is limited.

A recent systematic review found that isometric was not superior to isotonic exercises on pain due to chronic tendinopathies, except immediately post-intervention in patellar tendinopathy [17]. However, it’s important to note that these findings were derived from studies focusing on tendinopathies in various body regions and included only one study addressing individuals with rotator cuff tendinopathy, which compared isometric exercise with cryotherapy. Existing literature consistently demonstrates the advantages of resisted and progressive exercises in reducing shoulder pain and improving functioning of patients with RC tendinopathy when compared to non-resisted, non-progressive exercises, as well as placebo or no treatment [18]. This highlights the important role of progressive loading in the management and treatment of tendinopathy. However, it’s worth noting that there is limited literature to date that has explored isometric exercises as a treatment modality, particularly in terms of load progression [18]. Furthermore, there is a lack of studies that directly compare isometric exercises to other exercise modalities [19].

Therefore, we aim to compare the effects of isometric and isotonic exercises on shoulder pain, functioning, muscle strength, and electromyographic activity of individuals with RC tendinopathy. Medium-term effects on pain and functioning of individuals with RC three months after the treatment will also be evaluated. To our knowledge, this will be the first study comparing the effects of isometric exercises with load control (adapted from a protocol for patellar tendinopathy) with isotonic exercises on shoulder pain, functioning, isometric strength, and electromyographic activity of people with RC tendinopathy.

Rotator cuff muscles are constantly active during arm elevation movement to avoid translation of humeral head and dynamically stabilize the joint [20,21]. Thus, we believe isometric contraction is important for training these muscles. Moreover, isometric exercises present a good response on pain tolerance and temporal summation. Therefore, we hypothesize individuals with RC tendinopathy treated with isometric exercises will present greater improvements in shoulder pain, functioning, and neuromuscular control immediately after the protocol and after six weeks of treatment than isotonic exercises, and the effects on pain and functioning will last for at least three months after treatment.

## Methods

### Study design

A randomized clinical trial, with two treatment groups, will be developed in the Department of Physical Therapy of the Federal University of Rio Grande do Norte (UFRN), Brazil. The protocol was approved by the research ethics committee of the Federal University of Rio Grande do Norte (protocol CAAE—12107519.0.0000.5537 and approval no. 3.434.684 and no. 5.915.806) and registered in the Brazilian Clinical Trials Registry platform (RBR-3pvdvfk) with Universal Trial Number (UTN) code U1111-1284-7528. The study will follow SPIRIT (Standard Protocol Items: Recommendations for Interventional Trials) for protocol studies [22] and will be reported according to CONSORT (Consolidated Standards of Reporting Trials) recommendations for randomized clinical trials [23]. All procedures will be performed according to the Declaration of Helsinki, and all individuals will sign the informed consent from (Appendices 1) providing their written consent prior to participation after being advised, verbally and written, of the objectives, risks, and benefits of the study.

### Participants

Participants will be individuals of both genders, with supraspinatus and/or infraspinatus tendinopathy. They will be randomly distributed into two groups of resistance exercises for the RC muscles: isometric group (IMG), which will perform isometric resistance training, and isotonic group (ITG), which will perform isotonic resistance training.

Sample size was calculated (G*Power 3.1, Christian-Albrechts-Universitat, Kiel, Germany) using pain during arm elevation (no load) as the primary outcome, α = 0.05, and power of 80% for between-group analysis [24]. Considering a mean difference of 2.05 and a standard deviation of 2.30 in the numerical pain rating scale (NPRS) between groups [25], sample size was estimated as 21 individuals per group. We will add 10% in each group to account for potential sample losses, totalizing 48 individuals (24 per group).

A non-probability sampling of consecutive cases will be recruited (from July 1^st^, 2022 to September 30^th^, 2023) from a waiting list for physical therapy service of the Physical Therapy sector at UFRN, referral of an orthopedist, and advertising at the university and social media. We did not register this study before enrolment of participants started because some important modifications, such as age range of the participants, was submitted to the University’s Ethics Committee via an amendment and the implementations were made after respectively approval.

### Inclusion criteria

We will include individuals aged between 18 and 60 years, with shoulder pain for at least three months [26] considering the most recent acute episode of pain; at least one positive specific test in physical exam (Jobe test or resisted external rotation) [7]; and diagnosed with tendinopathy by an orthopedist through confirmed morphological alterations in the RC tendons (supraspinatus and/or infraspinatus) observed using nuclear magnetic resonance or ultrasonography [27].

### Exclusion criteria

We will exclude individuals that perform high-intensity sports with high shoulder demand [28]; impaired long head of biceps; adhesive capsulitis [29]; history of glenohumeral luxation or subluxation; history of clavicle, scapula, or humerus fracture [30]; history of RC surgical intervention [31]; signs of partial or complete rupture of RC [32]; acromioclavicular joint osteoarthritis [33]; neurologic [34] or rheumatologic dysfunctions; corticoid application at least three months before the baseline assessment [30]; body mass index>28 kg/m^2^ since it may compromise quality of electromyography data [30]; and individuals under treatment with fluoroquinolone antibiotics [35] or diabetes [36] because both may affect tendon metabolism.

### Procedures

Eligibility criteria will be assessed by a physical therapist with 6 years of experience (assessor one), who will screen the individuals, collect personal data and medical history, and perform a physical exam. The Portuguese version of the International Physical Activity Questionnaire—Short Form will assess the level of physical activity [37]. Based on screening, individuals with symptoms of RC tendinopathy will be referred to shoulder ultrasonography or magnetic resonance imaging that must be performed within two weeks.

**Fig 1:**
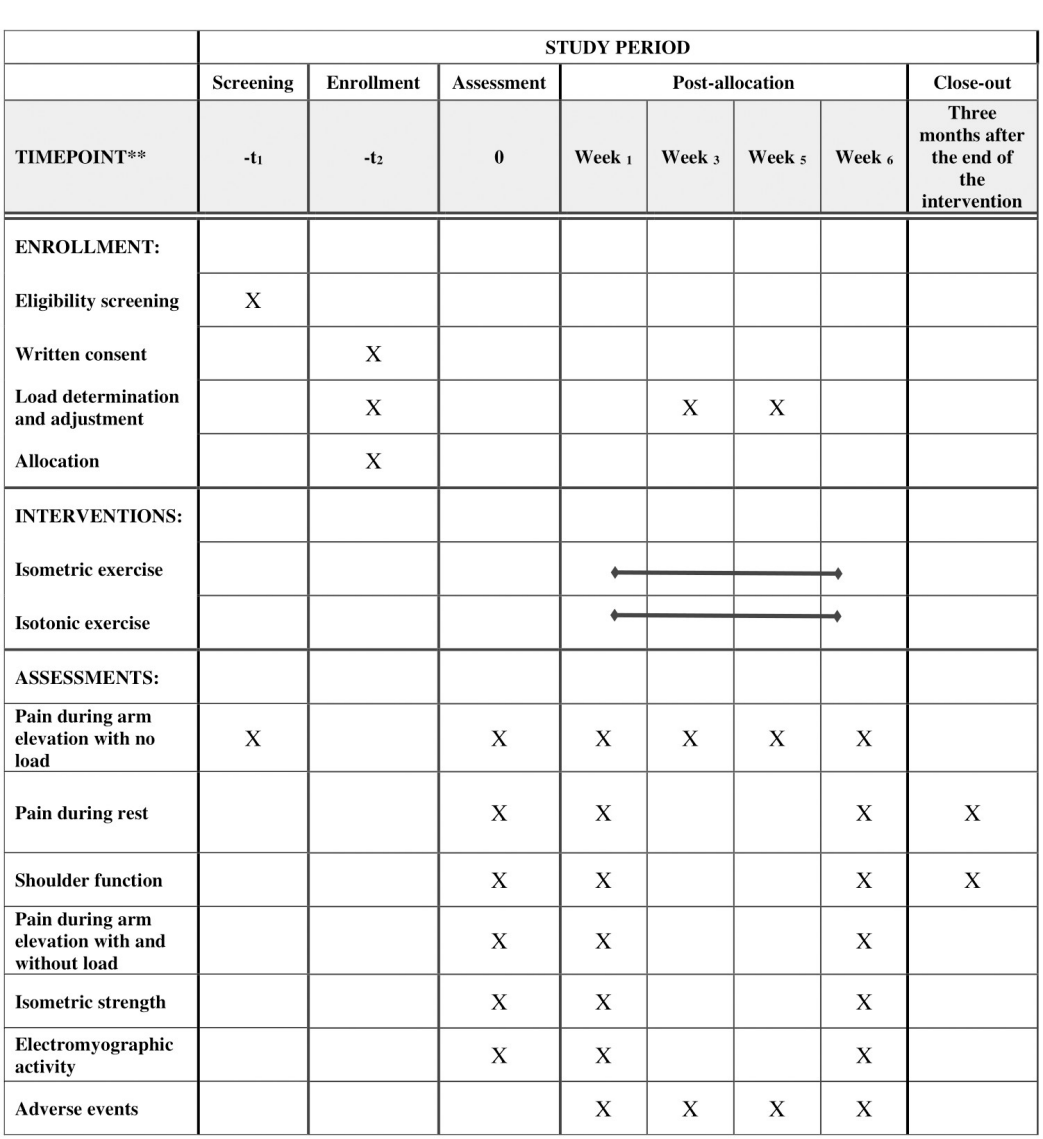
Schedule of enrollment, interventions, and assessments.

After results of imaging exams, if the participant meets inclusion criteria and is willing to participate in the study, they will sign the consent form and will be randomized into one of the two groups. Outcomes will be evaluated by assessor two, who performed reliability of measurements in 12 healthy participants. Primary outcome will be pain during arm elevation with no load. Secondary outcomes will be pain during rest, during isometric contraction, and resisted arm elevation; shoulder functioning; isometric strength; and electromyographic activity of shoulder muscles. Assessments will occur in four moments: a) pre-intervention; b) immediately after the first session, in which all variables will be assessed (except shoulder functioning); c) six months after initiating interventions; and d) follow-up, in which pain and functioning will be assessed, three months after finishing the intervention. Fig 1 shows the specific procedures of the study.

### Randomization and blinding

Individuals will be informed about intervention protocols (i.e., comparison between two types of exercise) and randomly allocated into IMG or ITG group after signing a written consent form. Individuals will be blinded since they will not know the exercise performed in the other group, and interventions will be performed individually.

**Fig 2:**
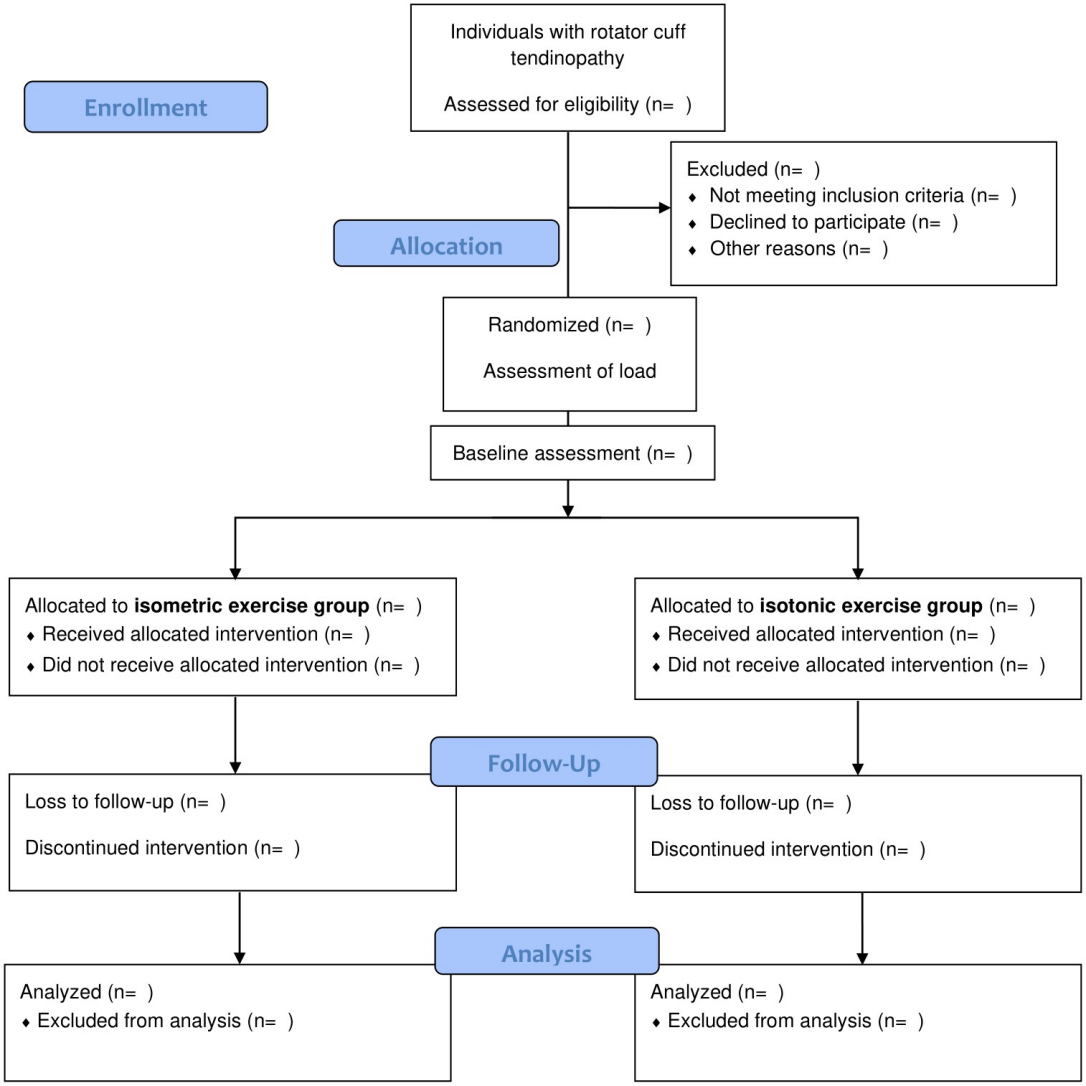
Flow diagram for consolidated standards of reporting trials.

An independent researcher who will not participate in any other procedure will perform block randomization using the website randomization.com with a 1:1 allocation ration. Allocation will be performed using opaque and sealed envelopes consecutively numbered, which will be opened by assessor one to determine resistance load after the individuals sign the written consent form. Assessor one will apply the intervention in both groups and instruct individuals to not talk with assessor two or any other participant about exercises performed. Also, assessor one will not participate in the assessment to minimize risk of bias. Only assessor two will perform assessments and will be blinded to interventions. Fig 2 shows the flowchart of participants and randomization.

### Reliability and training

Assessor two performed reliability of measures by assessing 12 healthy individuals. Intraclass correlation coefficient (ICC), standard error (SEM) and minimal detectable change (MDC) were obtained for strength measures. The strength was collected in Kgf and normalized against body mass and expressed as a percentage of body weight (BW%). For intraday reliability, ICC varied from 0.95 to 0.98, SEM varied from 0.58 BW% to 0.68BW%, and MDC from 0.82BW% to 0.96BW%. For interday reliability, ICC varied from 0.84 to 0.96, SEM varied from 0.77BW% to 1.05BW%, and MDC from 1.08B%W to 1.49BW%. Similarly, assessor one will familiarize with the intervention protocol and measures of load determination in ten individuals.

### Strategies to increase treatment adherence

Assessors will regularly communicate with individuals asking them about any discomfort, encouraging them to promptly inform any increase in pain, and guaranteeing that if needed some modifications to the treatment can be made. Also, interventions will be scheduled according to availability of individuals to keep them engaged and maintain continuity with the treatment.

### Assessments

#### Pain and general shoulder functioning

We will use the translated version of Penn Shoulder Score Questionnaire (Penn) and Western Ontario Rotator Cuff Index (WORC) to assess shoulder pain and functioning. Penn has three domains (pain, satisfaction, and function), and total score ranges from 0 to 100, in which 100 represents high functioning, low pain, and high satisfaction with shoulder functioning; minimum clinically important difference (MCID) of the questionnaire is 11.4 [38,39]. WORC has five domains regarding life and health quality (physical symptoms, sports/recreation, work, lifestyle, and emotions). Each item varies from 0 to 100 mm scored on a visual analog scale. Total score varies from 0 to 2100 mm and is converted into a percentage score, in which 0% represents the worst possible score and 100% implies no reduction of health-related quality of life [40,41]. The MCID of WORC has been shown to be 245.26 mm [42].

Current pain of individuals will be assessed using NPRS (0 to 10) before and after assessments, during strength assessment, and during arm elevation with and without load, as well as before and after the intervention sessions. The MDCI for NPRS in individuals with shoulder pain is 1.1 [43].

#### Isometric strength

**Fig 3:**
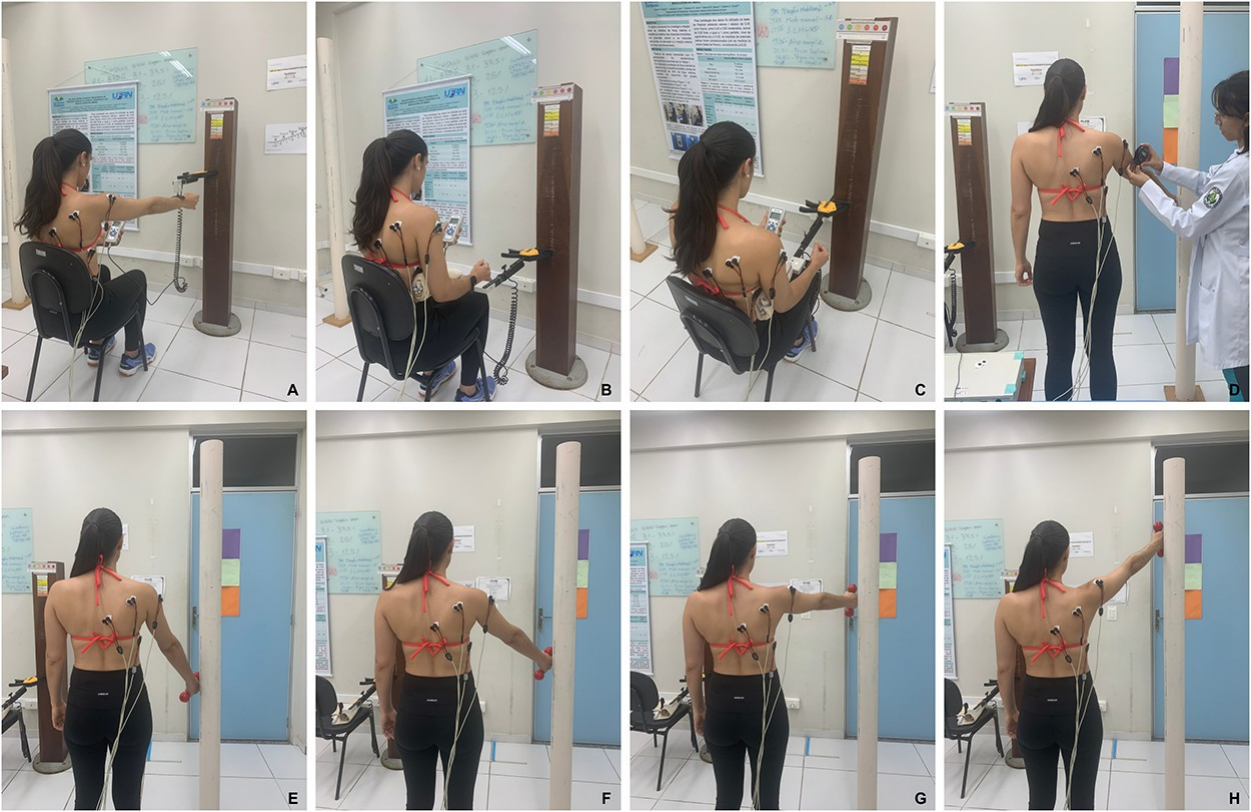
Positioning of electromyography and isometric strength test. (A) shoulder elevation, (B) external rotation, and (C) internal rotation; (D) use of inclinometer to measure degrees of arm elevation; isometric arm elevation at (E) 30, (F) 60, (G) 90, and (H) 120 degrees.

Maximal isometric strength during arm elevation and lateral and medial shoulder rotation will be measured using a dynamometer Nextech (DFS-X1000 model, Nextech Global Company Limited, Thailand) with the individuals in a seated position [44]. The dynamometer will be attached to a metal apparatus which is attached to an immoveable wooden column to ensure isometric contraction. For arm elevation, the shoulder will be elevated at 90° to the scapular plane. For rotations, the shoulder will be positioned at 0° of abduction, elbow flexed at 90°, and wrist in neutral position. Individuals will receive instructions to push the dynamometer with their maximum possible strength. The strength will be collected in Kgf, normalized against body mass, and expressed as BW%. Each test will be performed twice, with 5 s of contraction and 60-s interval between attempts, adapted from Bandholm et al. [28]. The mean value will be used for analysis, and participants will be verbally encouraged during each contraction. Fig 3A–3C shows the position for isometric strength testing.

#### Electromyographic activity

Electromyographic signals during maximal isometric contractions will be acquired from the following muscles using surface electrodes (Miotec^®^, Porto Alegre, RS, Brazil): middle deltoid, infraspinatus, serratus anterior, and lower trapezius. We will use a signal conditioning module (SCM 1000) of four channels (EMG system do Brazil^®^, São José dos Campos, SP, Brazil). Electrodes will be positioned according to Michener et al. [44], and the reference electrode will be fixed at the ulnar styloid process contralateral to the evaluated side [45]. Root mean square normalized to maximum peak value during pre-intervention assessment will be considered for analysis. These EMG measurements have been demonstrated to be reliable and valid tools in prior study [46].

The assessment of electromyographic signal will be performed during the isometric strength assessment of arm elevation and shoulder rotations, and during the isometric arm elevation in scapular plane with and without load at 30 (Fig 3E), 60 (Fig 3F), 90 (Fig 3G), and 120 degrees of elevation in the scapular plane (Fig 3H). For arm elevation with load participants will hold 1.5kg for those weighting 68 kg or less and 2.5kg for those above 68 kg of body weight [40]. Each angle will be determined using an Acumar digital inclinometer (Lafayette Instrument Company^®^, Lafayette, IN, USA) [47].

### Intervention protocol

**Fig 4:**
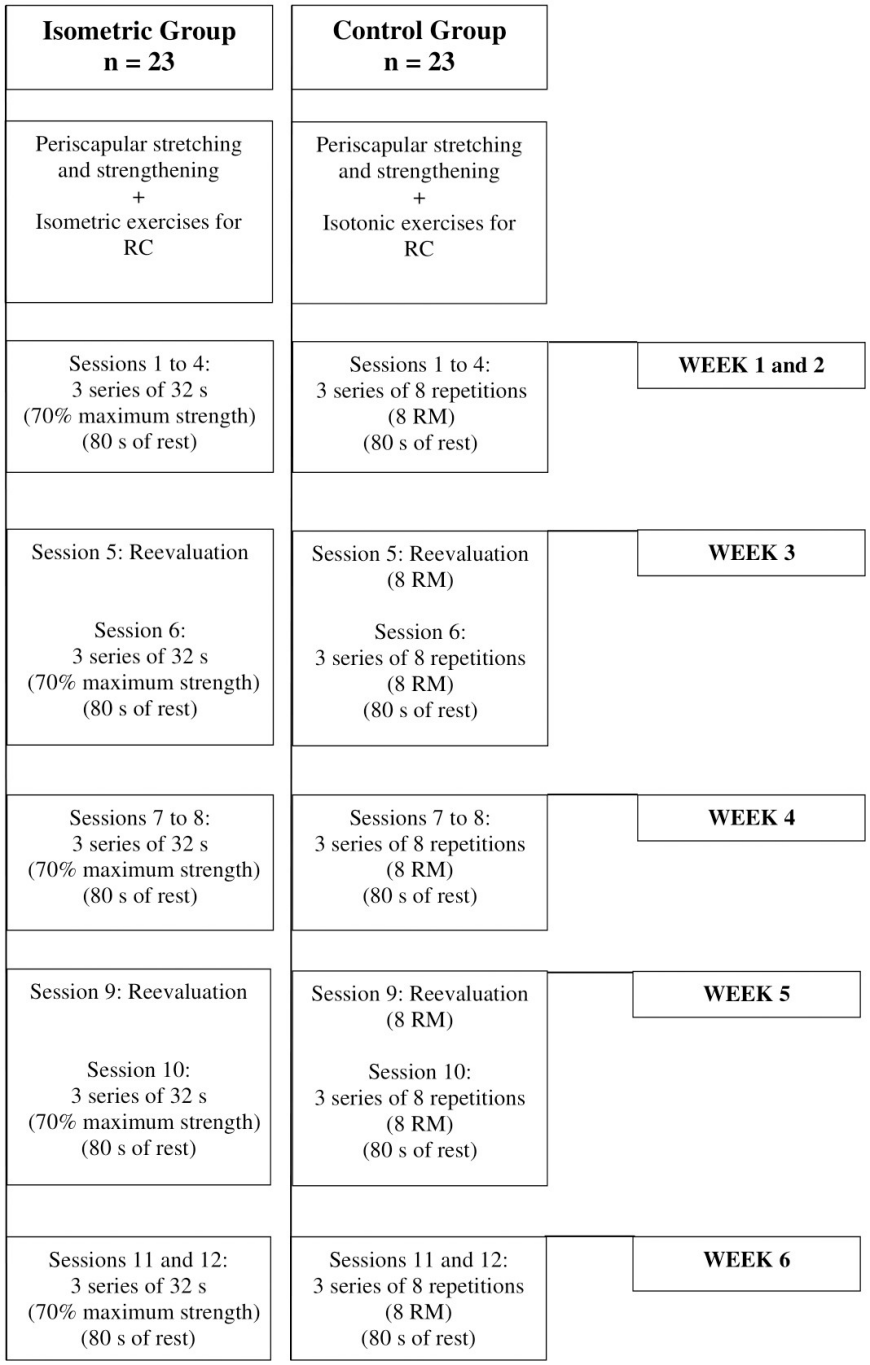
Flow diagram of intervention protocol and progression of groups. RC: Rotator cuff; s: Seconds; RM: Repetition maximum.

All participants will perform one exercise session on the assessment day, to verify the immediate effects of the exercises, followed by a six-week intervention (twice a week, with a minimum interval of 48 hours) to verify the effects of the RC strengthening program. Initial resistance load will be determined one week before the first assessment. Resistance load for both groups will be reevaluated in weeks three and five, as adapted from Rio et al. [11]. Contraction and relaxation time will be the same for both groups: 96 s and 80 s, respectively for RC strengthening. Participants will familiarize with each exercise using a light resistance and will be instructed regarding the correct technique. Fig 4 shows the flow diagram of intervention protocols.

**Fig 5:**
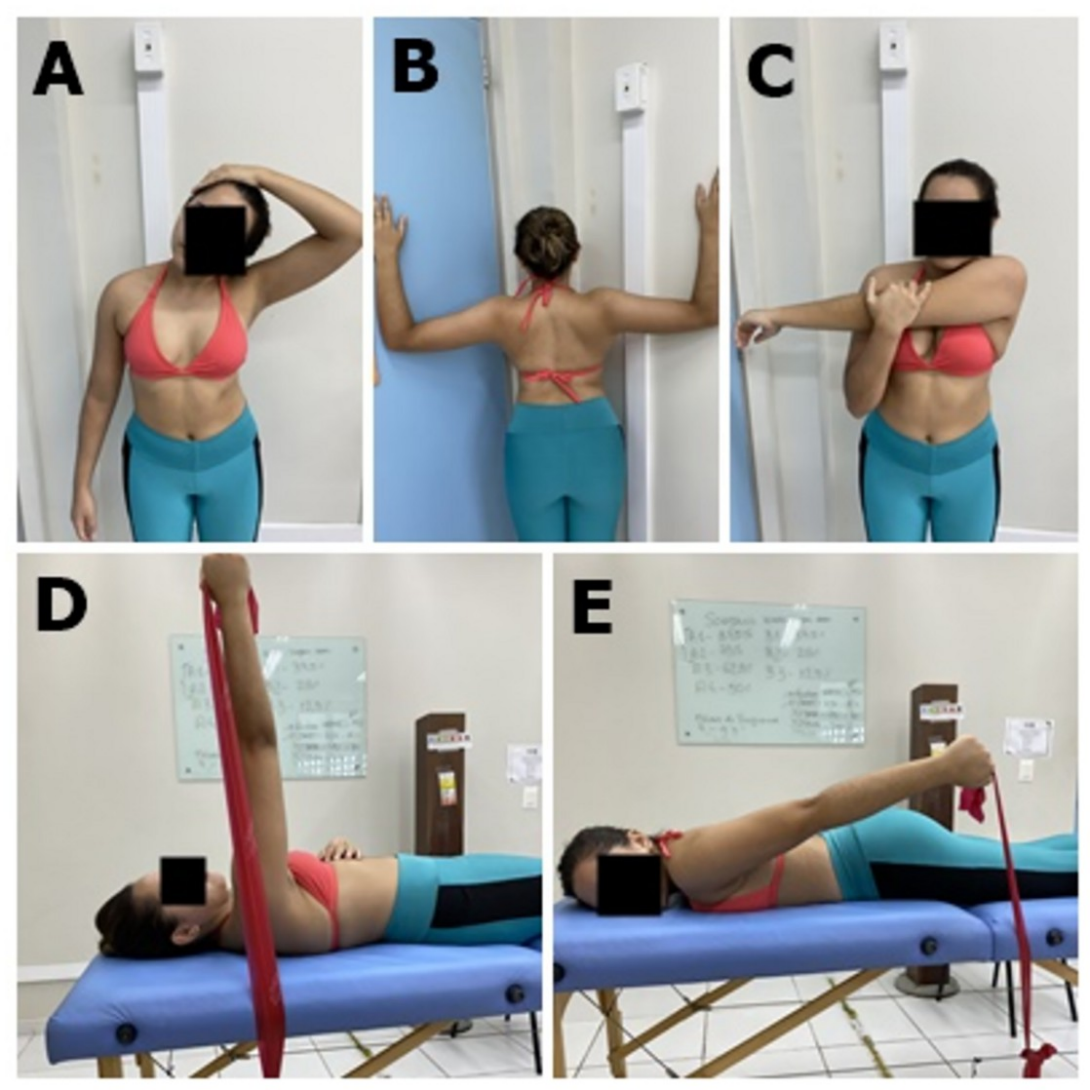
Stretching and strengthening of periscapular muscles. (A) upper trapezius, (B) pectoralis minor, (C) cross-body stretch, (D) serratus anterior and (E) lower trapezius.

Individuals from both groups will stretch and strengthen the periscapular musculature, following the protocol used by Camargo et al. [8]: stretching of upper trapezius, pectoralis minor, and cross-body stretch; and strengthening of serratus anterior and lower trapezius. Participants will perform three sets of stretching exercises, sustaining each limb for 30 s, with a 30 s interval between repetitions. Strengthening exercises will be performed in three sets of 10 repetitions for each exercise with 1-min intervals. Elastic bands with color progression (TheraBand: The Hygenic Corporation, Akron, OH) will provide resistance [8]. When performing the three series is easy for the individual, the resistance will be increased by changing the color of the elastic band (Fig 5).

All treatment sessions will be performed individually with a one-to-one supervision by a certified physical therapist who will encourage participants to keep the target force in the isometric group and to keep the pace following the metronome for the isotonic group.

We will use the OMNI-Resistance exercise scale 30 min after the load assessments and after the interventions in both groups [48] to monitor perceived effort and internal load of exercises, as well as observe whether the effort level is homogeneous between groups. The scale varies from 0 to 100 (0 represents extremely easy and 100 extremely difficult), and it will be shown to participants after each repetition [49]. NPRS will be applied at the beginning and end of each session and after each exercise series.

#### Protocol for isometric exercise

**Fig 6:**
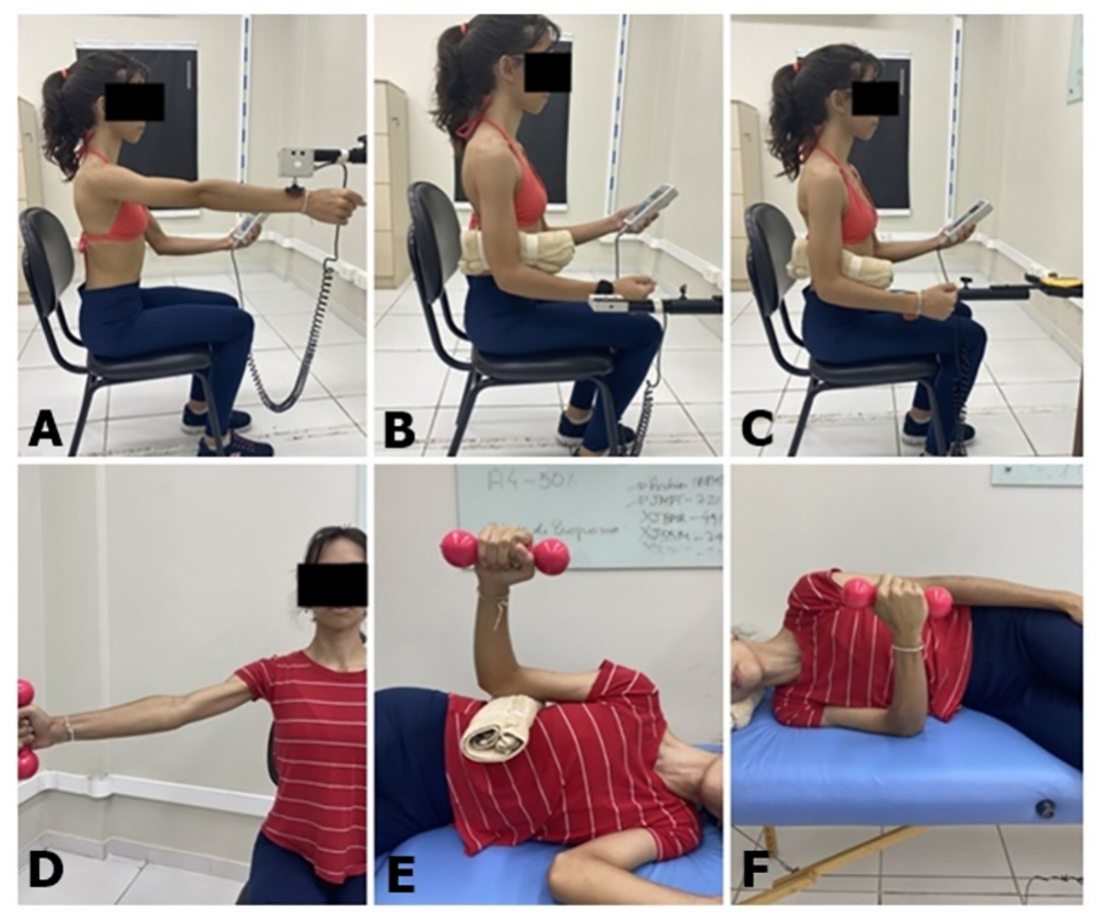
Positioning for intervention. (A) isometric shoulder elevation in scapular plane, (B) isometric external rotation, and (C) isometric internal rotation; (D) isotonic shoulder elevation in scapular plane, (E) isotonic external rotation, and (F) isotonic internal rotation.

Protocol for isometric exercises will involve submaximal isometric contractions of the RC muscles. Specifically, participants will perform isometric contraction of the arm at 90° of elevation in the scapular plane Fig 6A. External and internal rotations (Fig 6B and 6C, respectively) will be performed in the same positions of isometric strength assessment. Fig 6 shows the position of the interventions for both groups.

The protocol for isometric exercise was adapted from Rio et al. [14,15] for lower limb tendinopathy. Individuals will perform three sets, sustained for 32 s, at 70% of maximal voluntary isometric contraction (MVIC), resting for 80 seconds between sets [16]. Resistance load will be determined according to maximal isometric strength of elevation and external and internal rotations measured during the pre-intervention assessment. Visual feedback will be provided by the dynamometer during the entire intervention to ensure that the exercise will be maintained at 70% of MVIC. Participants will also be encouraged to keep the target force during the contractions.

#### Protocol for isotonic exercise

Protocol for isotonic exercises will involve concentric and eccentric strengthening exercises for the RC muscles using dumbbells. Exercises will consist of three sets of eight repetitions with intensity of eight repetition maximum (RM). Interval between sets will be 80 s [16]. To determine 8 RM (the maximum load with which the individual can lift 8 times maintaining the correct exercise technique) the individual will perform one to four attempts with 5-min rest between attempts to reach the load [50].

External rotation will be performed in side-lying, contralateral to the affected shoulder, at 0° abduction, neutral rotation and elbow flexion of 90° (a towel will be positioned between arm and trunk of individuals) (Fig 6E). For internal rotation, individuals will be positioned in side-lying over the affected shoulder, at 0° abduction, neutral rotation and elbow flexion at 90° [11] (Fig 6F). Individuals will be instructed to perform rotations starting from a neutral rotation until their maximum individual external and internal rotation.

For arm elevation exercise, individuals will perform elevations from 60° to 90° in the scapular plane, with the wrist in a neutral position and the thumb pointing upward (Fig 6D). This range of motion has a low risk of compressing the supraspinatus tendon in the coracoacromial arch, which could increase pain or cause additional injury [51].

Individuals will be instructed to perform contractions of 2 s for concentric phase and 2 s for eccentric phase, controlled by a metronome that will also serve as feedback during arm elevation and external and internal rotations.

If an individual experiences severe pain and is unable to perform movements in synchronization with the metronome in the isotonic group, we will adapt the resistance load to facilitate their compliance with the metronome cadence and maintain time under tension. Similarly, if an individual in the isometric group encounters difficulty in sustaining the 32-second contraction due to pain, we will adjust the load to enable them to maintain the required duration of the contraction. Any modifications made to the intervention for any participant will be thoroughly documented and reported.

In each assessment and intervention session, participants will be questioned about medication use and possible adverse events, pain in the supraspinatus, infraspinatus, long head of biceps muscle, or acromioclavicular joint, and increase in pain during or after the intervention. If pain increases and persists after the intervention, participants should contact the physical therapist in charge to make modification in the interventions, such as adjustment in loading maintaining time under tension controlled by the metronome. The percentage of people who experience pain during and/or after the intervention will be reported as adverse effects for both groups, and we will also report any modification needed in the intervention. The participants will be asked not to undergo another type of therapy during their participation in the study.

### Data analysis

Data will be collected manually on paper forms and transferred to a computer. Manual data entry will be conducted by two distinct individuals, and a specialized software application will be utilized to conduct a thorough comparison of the entered data. The data will be kept by the principal investigator in a safe computer protected by password at the University or in a locked cabinet, for the forms, for a period of five years, and will be assessed only by the study staff. After study completion the data can be shared by the principal investigator upon request.

Data will be analyzed by a blind statistician and will be presented descriptively and inferentially. Groups will be designed to statistician by distinct colors. Mean and standard deviation will be calculated for all demographic data and dependent variables. For primary and secondary outcomes, we will compare groups (IMG and ITG) and assessments using linear mixed models with repeated measures. The model includes group and assessment as fixed effects and subject as random effects. Interaction term between group and assessment will be of main interest. When appropriate, Tukey’s post hoc tests will be performed. Intention-to-treat (ITT) analysis will be performed to ensure randomization and distribution of prognostic factors uniformly between groups. In order to be included in ITT, participants must have completed at least 70% of the intervention. Missing data will be addressed in the linear mixed model without any hoc imputation, since this analysis shows less decrease in power when missing data is present. Analyses will be performed in the Statistical Package for the Social Sciences software, version 20.0, with significance level α ≤ 0.05 (two-tailed).

## Discussion

This manuscript describes a randomized clinical trial protocol to compare the effects of isometric and isotonic exercises on RC muscles of individuals with RC tendinopathy.

Isometric exercises have been shown to be promising in comparison to isotonic exercises for individuals with patellar tendinopathy [14–16,52]. Thus, we developed this protocol of isometric exercise, adapted from Rio et al. [14,15], to compare with commonly used isotonic exercises in individuals with RC tendinopathy. This study will demonstrate the effects of isometric and isotonic exercises immediately after the first intervention, after six weeks, and in the medium-term (three months after intervention) on shoulder pain, functioning, muscle strength, and electromyographic activity of individuals with RC tendinopathy.

In this protocol, we tried to minimize biases by randomizing individuals, blinding the assessor two (who will not know allocation of individuals), and blinding participants. Volume will be similar in both groups to identify possible effects of one modality over the other and avoid benefiting any group individually. Resistance load will be adjusted for both groups, considering adaptations of the neuromuscular system to exercises.

This protocol has some limitations, such as the absence of a control group with no intervention, to account for the natural history of the disease. However, we believe this limitation is minimized considering that the prognosis of wait-and-see approaches is not favorable in chronic tendinopathy. Also, the researcher responsible for the intervention will not be blinded due to the intervention characteristics.

The literature lacks studies regarding isometric exercises for improving pain, functioning, muscle strength and electromyographic activity of individuals with RC tendinopathy. Therefore, to our knowledge, this protocol will serve as basis for further elucidating the effects of this exercise modality in individuals with RC tendinopathy.

### Confidentiality

The data that will be provided is confidential and will only be disclosed in congresses or scientific publications, always anonymously, with no disclosure of any data that could identify participants. These data will be kept by the researcher responsible for this research in a safe place and for a period of 5 years.

### Ancillary and post-trial care

In case of any problem the participant may have related to the research, they will have the right to free assistance that will be provided by those responsible for the research at the University’s Physiotherapy service.

### Dissemination policy

The information is this protocol, and its outcomes will be presented to the community in form of a manuscript publication.

## Supporting information

S1 File SPIRIT checklist. (PDF)

S2 File Informed consent form. (PDF)

S3 File Study protocol approved by Research Ethics Committee English. (PDF)

S4 File Study protocol approved by Research Ethics Committee Portuguese. (PDF)
