# Correction: Isometric *versus* isotonic exercise in individuals with rotator cuff tendinopathy—Effects on shoulder pain, functioning, muscle strength, and electromyographic activity: A protocol for randomized clinical trial

**DOI:** 10.1371/journal.pone.0297630

**Published:** 2024-01-19

**Authors:** Bianca Rodrigues da Silva Barros, Denise Dal’Ava Augusto, João Felipe de Medeiros Filho, Lori Ann Michener, Rodrigo Scattone Silva, Catarina de Oliveira Sousa

The third author’s name is spelled incorrectly. The correct name is: João Felipe de Medeiros Filho. The correct citation is: Rodrigues da Silva Barros B, Dal’Ava Augusto D, de Medeiros Filho JF, Michener LA, Silva RS, Sousa CdO (2023) Isometric versus isotonic exercise in individuals with rotator cuff tendinopathy—Effects on shoulder pain, functioning, muscle strength, and electromyographic activity: A protocol for randomized clinical trial. PLoS ONE 18(11): e0293457. https://doi.org/10.1371/journal.pone.0293457
