# Correction: Correction: Isometric *versus* isotonic exercise in individuals with rotator cuff tendinopathy—Effects on shoulder pain, functioning, muscle strength, and electromyographic activity: A protocol for randomized clinical trial

**DOI:** 10.1371/journal.pone.0300483

**Published:** 2024-03-07

**Authors:** The PLOS ONE Staff

## Notice of Republication

There was an error in the names of the first and second authors in the correction published on January 19, 2024. This correction notice has been republished on February 15, 2024, to rectify the error. The publisher apologizes for the error. Please download this notice again to view the correct version.

## References

[1] Rodrigues da Silva Barros B, Dal’Ava Augusto D, de Medeiros Neto JF, Michener LA, Silva RS, Sousa CdO (2023) Isometric versus isotonic exercise in individuals with rotator cuff tendinopathy—Effects on shoulder pain, functioning, muscle strength, and electromyographic activity: A protocol for randomized clinical trial. PLoS ONE 18(11): e0293457. doi: 10.1371/journal.pone.0293457 37956135 PMC10642785

[2] Rodrigues da Silva Barros B, Dal’Ava Augusto D, Filho JFdM, Michener LA, Silva RS, Sousa CdO (2024) Correction: Isometric versus isotonic exercise in individuals with rotator cuff tendinopathy—Effects on shoulder pain, functioning, muscle strength, and electromyographic activity: A protocol for randomized clinical trial. PLoS ONE 19(1): e0297630. doi: 10.1371/journal.pone.0297630 38241237 PMC10798434

