# Supplementary material for: Isometric versus isotonic exercise in individuals with rotator cuff tendinopathy—Effects on shoulder pain, functioning, muscle strength, and electromyographic activity: A protocol for randomized clinical trial
Source: PLoS One. 2023 Nov 13;18(11):e0293457. doi: 10.1371/journal.pone.0293457 (PMC10642785; doi:10.1371/journal.pone.0293457)
Supplement: S2 File — (PDF) [file pone.0293457.s002.pdf]

FEDERAL UNIVERSITY OF RIO GRANDE DO NORTE
HEALTH SCIENCE CENTER
DEPARTMENT OF PHYSICAL THERAPY

INFORMED CONSENT

This is an invitation to participate in the research: isometric *versus* isotonic exercise in individuals with rotator cuff tendinopathy – effects on shoulder pain, function, muscle strength, and neuromuscular control: a randomized clinical trial, developed by the PhD student in Physiotherapy Bianca Rodrigues da Silva Barros, whose principal investigator is Professor Catarina de Oliveira Sousa.

The aim of this research is to compare and characterize the effects of two types of shoulder strengthening exercises on shoulder pain, function, and control in individuals with rotator cuff tendinopathy, a muscle group responsible for stabilizing the shoulder.

The reason that led us to carry out this study is to better understand the effects and characteristics of strengthening exercises used in the treatment of shoulder disorders. Conservative treatment, based on physiotherapeutic intervention, is indicated for the treatment of tendinopathies and ruptures of the rotator cuff muscles, especially resistance training that progressively imposes load on the tendon, in order to help it to repair by altering its metabolism, mechanical and structural properties. Among the various forms of strengthening, eccentric and concentric exercises (which are performed with shoulder movement) have been shown to be effective in improving general shoulder function, and few studies have been developed evaluating the effects of isometric exercise (performed without shoulder motion) in rotator cuff tendinopathy.

If you have rotator cuff tendinopathy diagnosed by an orthopedic surgeon through MRI or ultrasound imaging, and you decide to accept the invitation, you will undergo some assessment and intervention procedures. The assessment procedures will occur in three moments, and you will need to come in three different days. The intervention procedures will be done twice a week for a total period of six weeks. Your participation is voluntary, and you may withdraw from the study at any time if you wish. In the first moment of the evaluation, you will be examined by a physical therapist with six years of experience. This

evaluation consists of taking personal data and medical history, and physical examination to see if you have signs and symptoms of tendinopathy in one of the rotator cuff muscles, and to exclude other painful shoulder conditions.

After completing the initial assessment, you will undergo an evaluation to determine the load and familiarize yourself with the exercises which will be performed during intervention. Between 3 and 7 days you will be scheduled to the assessment of shoulder pain, function, electromyography activity of muscles, and analysis of shoulder movement. These assessments will be repeated in two other moments: right after the first training session with the exercises and after the total training period, at the end of the 6 weeks. For the evaluation of the electrical activity of the muscles and movement, sensors will be attached with double-sided adhesives and hypoallergenic tape at specific anatomical points on the shoulder and you will be instructed to perform some arm elevation movements. These sensors do not generate any stimulation or painful sensations.

After these evaluation procedures, you will participate in a 6-week training program, according to the group you will be allocated to by lot: 1) exercises involving neck and shoulder musculature stretching, strengthening of the muscles that move the scapula and strengthening of the rotator cuff muscles isometrically; or 2) exercises involving neck and shoulder muscle stretching, strengthening the muscles that move the scapula and isotonic strengthening of the rotator cuff muscles.

All procedures, from assessments to the intervention program, will be performed at the Neuromuscular Performance Analysis Laboratory at the Physical Therapy Department of the Federal University of Rio Grande do Norte (UFRN), ensuring complete privacy for study participants.

The assessments and interventions are not invasive, however, during the research, occasional muscle discomfort may occur as a result of performing the physical exercises, which can be minimized with the application of cryotherapy to reduce these discomforts.

As benefits from the research, you will receive a complete assessment of your shoulder and receive a training program aimed at reducing pain and improving function and motor control of your shoulder. If we observe at the end of the research a superiority

in the results of one group over the other, we will guarantee the participants that treatment protocol with the best results.

In case of any problem that you may have related to the research, you will have the right to free assistance that will be provided by those responsible for the research at the UFRN Physiotherapy service.

During the entire period of the research, you will be able to resolve your doubts by calling Professor Catarina de Oliveira Sousa (responsible researcher) on the phone (84) 99959-6269 or by e-mail sousa.catarinaoliveira@gmail.com.

You have the right to refuse to participate or withdraw your consent at any stage of the research, without prejudice to you.

The data you will provide us will be confidential and will only be disclosed in congresses or scientific publications, always anonymously, with no disclosure of any data that could identify you. These data will be kept by the researcher responsible for this research in a safe place and for a period of 5 years.

Any eventual expenses incurred by your participation in this research, they will be assumed by the responsible researcher and reimbursed to you.

If you suffer any damage because of this research, whether immediate or delayed, foreseen or unforeseen, you will be indemnified.

If you have any questions about the ethics of this research, you should call the Research Ethics Committee – an institution that assesses the ethics of research before they begin and provides protection to participants – at the Federal University of Rio Grande do Norte, on the telephone number (84) 3215-3135 / (84) 9.9193.6266, via email cepufrn@reitoria.ufrn.br. You can still go in person to their headquarters, from Monday to Friday, from 08:00h to 12:00h and from 14:00h to 18:00h, at the Federal University of Rio Grande do Norte, Av. Senator Salgado Filho, s/n. Central Campus, Lagoa Nova. Natal, RN.

This document has been printed in duplicate. One will stay with you and the other with the responsible researcher Professor Catarina de Oliveira Sousa.

Statement of consent

After being clarified about the objectives, importance and way in which the data will be collected in this research, in addition to knowing the risks, discomforts and benefits

that it will bring to me and having become aware of all my rights, I agree to participate in the research " Isometric versus isotonic exercise in rotator cuff tendinopathy – effects on pain, function, muscle strength and neuromuscular control: a randomized clinical trial", and I authorize the disclosure of the information provided by me in congresses and/or scientific publications as long as no data can identify me.

Natal (RN), / / .

Participant's fingerprint

Participant's signature

Statement by the principal investigator

As the principal investigator of the study "Isometric versus isotonic exercise in rotator cuff tendinopathy – effects on pain, function and neuromuscular control: a randomized clinical trial", I declare that I assume full responsibility for faithfully complying with the methodological procedures and rights that have been clarified and assured to the participant of this study, as well as maintaining secrecy and confidentiality about his/her identity.

I also declare that I am aware that in failing to comply with the commitment assumed herein, I will be violating the rules and guidelines proposed by Resolution 466/12 of the National Health Council - CNS, which regulates research involving human beings.

Natal _____/_____/_______.

____

Prof. Dr. Catarina de Oliveira Sousa

Principal investigator
