# Supplementary material for: Isometric versus isotonic exercise in individuals with rotator cuff tendinopathy—Effects on shoulder pain, functioning, muscle strength, and electromyographic activity: A protocol for randomized clinical trial
Source: PLoS One. 2023 Nov 13;18(11):e0293457. doi: 10.1371/journal.pone.0293457 (PMC10642785; doi:10.1371/journal.pone.0293457)
Supplement: S3 File — (PDF) [file pone.0293457.s003.pdf]

FEDERAL UNIVERSITY OF RIO GRANDE DO NORTE
HEALTH SCIENCE CENTER
POSTGRADUATE PROGRAM IN PHYSICAL THERAPY
DEPARTAMENT OF PHYSICAL THERAPY

BIANCA RODRIGUES DA SILVA BARROS

ISOMETRIC VERSUS ISOTONIC EXERCISE IN INDIVIDUALS WITH ROTATOR CUFF TENDINOPATHY – EFFECTS ON PAIN, FUNCTION AND NEUROMUSCULAR CONTROL – A RANDOMIZED CLINICAL TRIAL

NATAL – RN
2019

FEDERAL UNIVERSITY OF RIO GRANDE DO NORTE
HEALTH SCIENCE CENTER
POSTGRADUATE PROGRAM IN PHYSICAL THERAPY
DEPARTAMENT OF PHYSICAL THERAPY

BIANCA RODRIGUES DA SILVA BARROS

ISOMETRIC VERSUS ISOTONIC EXERCISE IN INDIVIDUALS WITH ROTATOR CUFF TENDINOPATHY – EFFECTS ON PAIN, FUNCTION AND NEUROMUSCULAR CONTROL – A RANDOMIZED CLINICAL TRIAL

Advisor: Dr. Catarina de Oliveira Sousa

Doctoral research project presented to the Committee for Ethics and Research on Human beings of the Federal University of Rio Grande do Norte – CEP/UFRN.

NATAL – RN

2019

ABSTRACT

The aim of this project is to characterize the effects of two types of exercise – isometric versus isotonic – on shoulder pain and function and the relationship with neuromuscular control in individuals with rotator cuff (RC) tendinopathy. Forty-six individuals (18-60 years old) with shoulder pain (>3 months) and proven unilateral tendinopathy in the supraspinatus and/or infraspinatus by means of ultrasound or magnetic resonance imaging will participate in this study. Subjects will be randomly allocated to one of two exercise groups: isometric and isotonic. They will be evaluated before and after the first treatment session, and again after six weeks of treatment. The outcomes to be evaluated are: a) pain and general shoulder function, using the Penn Shoulder Score and The Western Ontario Rotator Cuff Index questionnaire; b) neuromuscular control through the electromyographic activity of the trapezius (lower portion), serratus anterior, deltoid (middle portion), and infraspinatus, during maximal isometric contractions of shoulder elevation at 90º, internal and external rotation of the shoulder at 0º of arm elevation and during elevation of the arm at 30, 60, 90 and 120 degrees with and without load using a surface electromyographic signal recording system (EMG system do Brazil®); we will evaluate the amplitude of muscular activity of each muscle and the ratio between muscle pairs: 1) infraspinatus and middle deltoid; 2) infraspinatus and lower trapezius; and 3) lower trapezius and serratus anterior; and c) functional performance through tests that simulate activities of daily living, which include: reaching a point above the head; reach the back of the head; and reaching the back or the opposite scapula, where the performance time and levels of pain and effort will be evaluated. The two groups will undergo a protocol of stretching and strengthening of the periscapular musculature. The isometric group will perform additional exercises for the RC, with 3 repetitions of 32s at 70% of the maximum isometric force for each exercise. The isotonic group will perform exercises for RC in a concentric and eccentric way, with 3 sets of 8 repetitions for each exercise at 8 RM. The exercise load of the two groups will be adjusted in weeks 3 and 5 of the six-week protocol. Treatment effects between groups will be evaluated using two-way ANOVAs with repeated measures, with the aid of the Statistical Package for the Social Sciences (SPSS) software. The results of this study will contribute to knowledge in the field of assessment and rehabilitation of the shoulder complex and may support decision-making process for treating RC tendinopathy.

Keywords: Pain management, exercise therapy, electromyography, subacromial impingement syndrome.

Summary

1 Introduction ... 5

2 Rationale ... 8

3 Aims ... 9

3.1 **Main aim** ... 9

3.2 **Specific aims** ... 9

4 Hypothesis ... 9

5 Methods ... 9

5.1 Characteristics of the study ... 9

5.2 Sample ... 10

5.3 Eligibility Criteria ... 11

5.4 Evaluation procedures ... 12

5.4.1 Pain and function assessment ... 14

5.4.2 Assessment of electromyographic activity during maximal isometric contraction and during arm elevation ... 14

5.4.3 Functional performance assessment ... 16

5.5 Intervention Protocols ... 17

5.5.1 *Stretching and strengthening of the periscapular muscles* ... 18

5.5.2 *Isometric Exercise Group* ... 18

5.5.3 *Isotonic Exercise Group* ... 19

5.6 Ethical aspects ... 20

5.7 Statistical analysis ... 21

6 Outcome and Expected Results ... 21

7 Timeline ... 24

8 Detailed and justified budget ... 25

References ... 26

Attachments ... 32

Appendices ... 40

1 Introduction

Shoulder pain is a common problem, being the third most frequent cause of musculoskeletal complaints (1). The dysfunctions that affect the shoulder can be characterized by limitations of range of motion due to pain and restrictions in activities of daily living, reducing functional independence and affecting the quality of life of affected individuals (2). Among the most common causes of shoulder pain is rotator cuff (RC) dysfunction, which consists of tendinopathy of one or more of the four RC muscles, which can progress to partial or total rupture of its tendons, associated or not with inflammation of shoulder bursae (3,4).

Effective shoulder movement and function is achieved through a combined effort of the scapula stabilizing muscles, deltoid, RC muscles, as well as the latissimus dorsi and pectoralis major (4). However, it is known the coordinated activity of the RC muscles (supraspinatus, infraspinatus, teres minor and subscapularis) is responsible for mechanical stability of the glenohumeral joint through stabilization or compression of the humeral head within the glenoid fossa during shoulder movements. (3.4).

Multiple factors may contribute to the development of RC tendinopathy, which are classified as: extrinsic, which originate externally to the tendons causing compression or shear to them, due to anatomical and biomechanical changes; and the intrinsic factors, which originate due to alterations in the mechanical, morphological and vascular properties of the tendons, as well as genetic predisposition (5,4). Both factors may be related and be potentiated with age and excessive mechanical load (5,6,3,4).

RC tendinopathy, related to subacromial impingement syndrome, has been associated with changes in motor control, since it is well described in the literature that there are changes in scapular kinematics during arm elevation, such as a reduction in posterior tilt and upward rotation and increase in the internal rotation of the scapula in individuals with subacromial impingement (7,8). In association with the movements, an altered activation of the periscapular musculature is verified during arm elevation, such as a decrease in the activation of the serratus anterior and the middle and lower portions of the trapezius (9,10), and an increase in the activation of the upper portion of the trapezius (11, 12,2,9). In addition, failure in the coordination between the lower trapezius and the

serratus anterior and between the upper and lower portions of the trapezius is verified, evidenced by the alteration of the activation ratio between these muscles (13).

Regarding the activation of RC muscles, there is reduced co-activation of RC and increased activation of the middle deltoid at the beginning of humeral elevation (14) and of the posterior deltoid, when the RC is fatigued, injured or ruptured, as a compensation to avoid reduction of the arm elevation movement and stabilize the glenohumeral joint(15,16). This imbalance between RC and deltoid activation may contribute to excessive superior translation of the humeral head (17) and, thus, greater risk for developing subacromial impingement syndrome and RC disease.

Although these muscle and movement changes are verified, there is little evidence to support whether these changes are caused by pain arising from the clinical condition or whether the pain is from the cumulative effect of changes in muscle activation and movement. Furthermore, the cause of local pain in tendinopathy remains elusive, as the level of pain varies substantially and is not always related to local pathology, suggesting a central sensitization in individuals with RC tendinopathy (3). Thus, in addition to focusing on interventions that reduce the risk of subacromial impingement, it is important to focus on interventions aimed at improving pain, in order to investigate the relationship between the clinical condition, pain and neuromuscular coordination in these individuals.

A primary intervention to treat RC tendinopathy is active therapeutic exercise (3), which has provided similar results to those who underwent surgical interventions, but with additional benefits of exercise, which are: less absenteeism, quick return to work and reduced cost to the health systems (18). Many exercise strategies have been proposed, however, uncertainty persists regarding the most effective one to treat the pain, weakness and loss of function associated with RC tendinopathy (3). However, pain reduction is a priority (3) and exercises with mechanical load, which stimulate tendon healing responses, accelerate the metabolism of tenocytes and tissue repair (19,20).

In this sense, resistance training in general, including isometric and isotonic exercises, focusing on both RC and scapular stabilizer muscles, in

patients with RC tendinopathy and subacromial impingement syndrome, has been shown to be effective in improving pain and function. (21). Comparison of isolated eccentric or concentric exercise interventions found no difference in efficacy, with both resulting in improvements in function, range of motion, and strength (22). Regarding isometric contractions, there is evidence that they help control pain when used in lower limb tendinopathies, but there is still no conclusive evidence about their effects on RC tendinopathies (23).

High load isotonic exercises have been shown to be effective for rehabilitation of lower limb tendinopathies, such as patellar tendinopathy (24) and Achilles tendinopathy (25). However, in recent years, isometric exercises have emerged as an excellent tool for tendinopathy rehabilitation (26–30). Isometric exercises have been shown to be more effective for quadriceps pain and function, both with short and long contraction durations (26), and more effective for reducing pain, increasing strength, and reducing cortical inhibition when compared with isotonic exercise, immediately (27) and after 4 weeks (28) when used in the treatment of patellar tendinopathy. Furthermore, submaximal isometric exercise has been shown to increase pressure pain threshold, reduce heat pain perception and reduce pain temporal summation in men and women (29). Applied to RC tendinopathy, we currently have data from a pilot study (30), which suggests that low-load isometric exercises for RC tendinopathy may positively influence pain and tendon stiffness.

Although these studies indicate positive effects of isometric exercises on pain, function and reduction of cortical inhibition in individuals with tendinopathy, to our knowledge, no studies have evaluated the effects of an isometric RC strengthening program on pain, function and neuromuscular control among RC muscles and periscapular and deltoid muscles during the arm raise task compared to an isotonic RC strengthening program. Thus, the aim of this study is to verify the effects of isometric exercise, applied to the RC muscles, compared to isotonic exercise, on pain, function and neuromuscular control in individuals with RC tendinopathy.

2 Rationale

RC tendon disease, both symptomatic and asymptomatic, have a high

prevalence in the general population, reaching up to 30% (31). This dysfunction is related to biomechanical factors, life habits and genetic predisposition, and influenced by age and overload to the tendons (5,32). The great clinical relevance of this condition is due to the fact that there is a high risk of progression of the tendinopathy to partial or total rupture of the tendons, causing symptoms of pain, weakness (32), and important functional limitations (33).

Conservative treatment, based on physiotherapeutic intervention, is indicated for treating RC tendinopathies and ruptures (32), especially resistance training which progressively imposes load on the tendon, in order to help in its repair by altering its metabolism and mechanical and structural properties (6). Among the various forms of resistance, eccentric and concentric exercises have been shown to be effective in improving overall shoulder function (22), and few studies (4,30) have been developed evaluating the effects of isometric exercise on RC tendinopathy.

However, isometric exercise has emerged as an important approach to improve pain in tendinopathies (27–29) and, when compared to isotonic exercise, it has been shown to be superior in terms of pain, strength, function in general, as well as in the reduction of cortical inhibition in the treatment of patellar tendinopathy (27). Thus, the rationale is the clinical relevance of evaluating the effects of isometric exercise applied to RC tendinopathy on pain, function and neuromuscular control involved in the shoulder complex. In this context, this study can help in clinical decision-making regarding the best approach to be considered in the treatment of RC tendinopathy.

3 Aims

3.1 Main aim:

To characterize the effects of two types of exercise – isometric and isotonic – on shoulder pain and function and the relationship with neuromuscular control in individuals with RC tendinopathy.

3.2 Specific aims:

- To evaluate the repercussions of isometric and isotonic exercises on pain and function in individuals with RC tendinopathy;
- To verify the effects of isometric and isotonic exercises on neuromuscular control of the shoulder complex muscles through electromyographic activity during maximal isometric contractions and on the motor task of arm arm elevation in individuals with RC tendinopathy;
- To analyze the relationship between effects on pain, neuromuscular control, functional performance and function of the shoulder complex;
- To verify the effects of isometric exercise compared to isotonic exercise on the variables described, immediately after an exercise session and after six weeks of intervention in individuals with RC tendinopathy.

4 Hypothesis

According to the positive and superior results presented from the isometric exercise applied to patellar tendinopathy in relation to the isotonic exercise, both immediately and after the period intervention, in pain, strength and function of the quadriceps, we hypothesize that this exercise modality will be superior to the exercise isotonic in individuals with RC tendinopathy, both immediately and after 6 weeks of intervention. We hypothesize that isometric exercise can reduce pain and improve functional performance and neuromuscular control of the shoulder complex, thus resulting in better function in this population.

5 Methods

5.1 Characteristics of the study

This is a randomized clinical trial, which will consist of two groups, and developed at the Department of Physiotherapy at the Federal University of Rio Grande do Norte.

5.2 Sample

Forty-six individuals with a clinical diagnosis of unilateral tendinopathy of the supraspinatus and/or infraspinatus tendon by means of ultrasonography (US) or nuclear magnetic resonance (NMR) exams, between 18 and 60 years of age, of both sexes will participate in this study. They will be randomly divided and allocated into two exercise groups, with 23 individuals each: 1) isometric group, which will undergo an isometric resistance training protocol for the RC musculature associated with a stretching and strengthening protocol focused on the periscapular musculature; and 2) isotonic group, which will undergo an isotonic resistance training protocol for the RC musculature associated with a stretching and strengthening protocol focused on the periscapular musculature.

Sampling will be non-probabilistic type of consecutive cases, where individuals will be recruited from a waiting list for physiotherapeutic care in the Physical Therapy Department of UFRN and through dissemination carried out at UFRN, in the city of Natal-RN and social media through posters. The sample size was determined considering the main outcome variable, pain with arm elevation without load, considering a difference between means of 2.05 and standard deviation of 2.30 in the numerical pain rating scale (NPRS) (34). Adopting a 80% Power and $\alpha = 0.05$, at least 21 individuals per group will be required. Considering possible sample losses of approximately 10%, the sample should be at least 23 individuals per group.

This project will be sent to the Ethics and Research Committee of UFRN (CEP/UFRN), through the registration in Plataforma Brasil, and will only start its activities after approval. All volunteers will receive a verbal and written explanation of the objectives and methodology of the study, as well as its risks and benefits; and those who agree to participate must sign an informed consent form, in accordance with Resolution 466/12 of the National Health Council, confirming their participation in the study. The project will be submitted to the Brazilian Registry of Clinical Trials (http://www.ensaiosclinicos.gov.br/) before

starting to recruit individuals.

5.3 Eligibility Criteria

Individuals of both sexes aged 18 to 60 years, with a history of shoulder pain for more than 3 months, and with a clinical diagnosis of rotator cuff tendinopathy by means of US or MRI exams, performed by a physician, will be included in the study (4).

Then, a physiotherapist with 6 years of experience will carry out an evaluation of the recruited individuals with the collection of personal data and clinical history, and physical examination, which will consist of palpation of the rotator cuff tendons, evaluation of the amplitude of the painful arc during arm elevation in the three planes of active and resisted shoulder movement, and the performance of the following special tests to detect dysfunction in the supraspinatus and infraspinatus tendons: Jobe's test to assess supraspinatus tendon injury (35), resisted external rotation for infraspinatus injury (36), apprehension and replacement tests for injury in the posterior-superior portion of the infraspinatus, characteristic of internal impact (35).

The diagnosis of RC tendinopathy will be performed by an orthopedic surgeon specialized in the shoulder complex, according to the clinical history, physical examination and morphological alterations of RC tendons, visualized by means of MRI or US: hypoechogenicity of the tendon and /or thickening of tendon fibers with or without bursal thickening (37).

Individuals will be excluded if they practice sports activities with high demand on the upper limbs; have impairments in the long head of the biceps; adhesive capsulitis (38); have history of symptom onset due to glenohumeral dislocation or subluxation, or fracture of the clavicle, scapula, or humerus (39); history of surgical stabilization or repair of the rotator cuff (40); signs of partial or complete rupture of the rotator cuff (12,40); neurological disorders (41); use of corticoid injection 3 months before the evaluation (39) and body mass index (BMI)>28kg/m2, since this can compromise the quality of the electromyography data (39), and individuals who are using antibiotics from the class of fluoroquinolones (42) and/or with diabetes (43), as both affect tendon metabolism.

5.4 Evaluation procedures

All included individuals will undergo the following assessments: a) pain and general function of the shoulder complex; b) electromyographic activity of the shoulder complex musculature during maximal isometric contractions; and c) electromyographic activity of the shoulder complex muscles during arm elevation in the scapula plane.

This evaluation sequence will be carried out in three moments: 1) initial evaluation, immediately before the first intervention session; 2) immediately after the first exercise session; and 3) after six weeks of intervention. All evaluations will be carried out by an evaluator who will not know which intervention group the individuals belong to (blind evaluator), with the interventions applied by two other trained researchers.

One week before the initial assessment, individuals will be randomly allocated using the online randomization tool for randomized clinical trials, available on the website (www.randomization.com). To guarantee the confidentiality of the allocation of individuals, this will be carried out by one of those responsible for applying the intervention, who will be the only one to know which group the individuals belong to. After determining the group, individuals will be introduced to the evaluation process and equipment, and will undergo a test to determine the elastic band to be used during periscapular muscle strengthening exercises. In addition, individuals allocated to the isometric or isotonic exercise group will perform the initial load determination test for each of the exercises. The sequences of the study procedures are described in Figure 1.

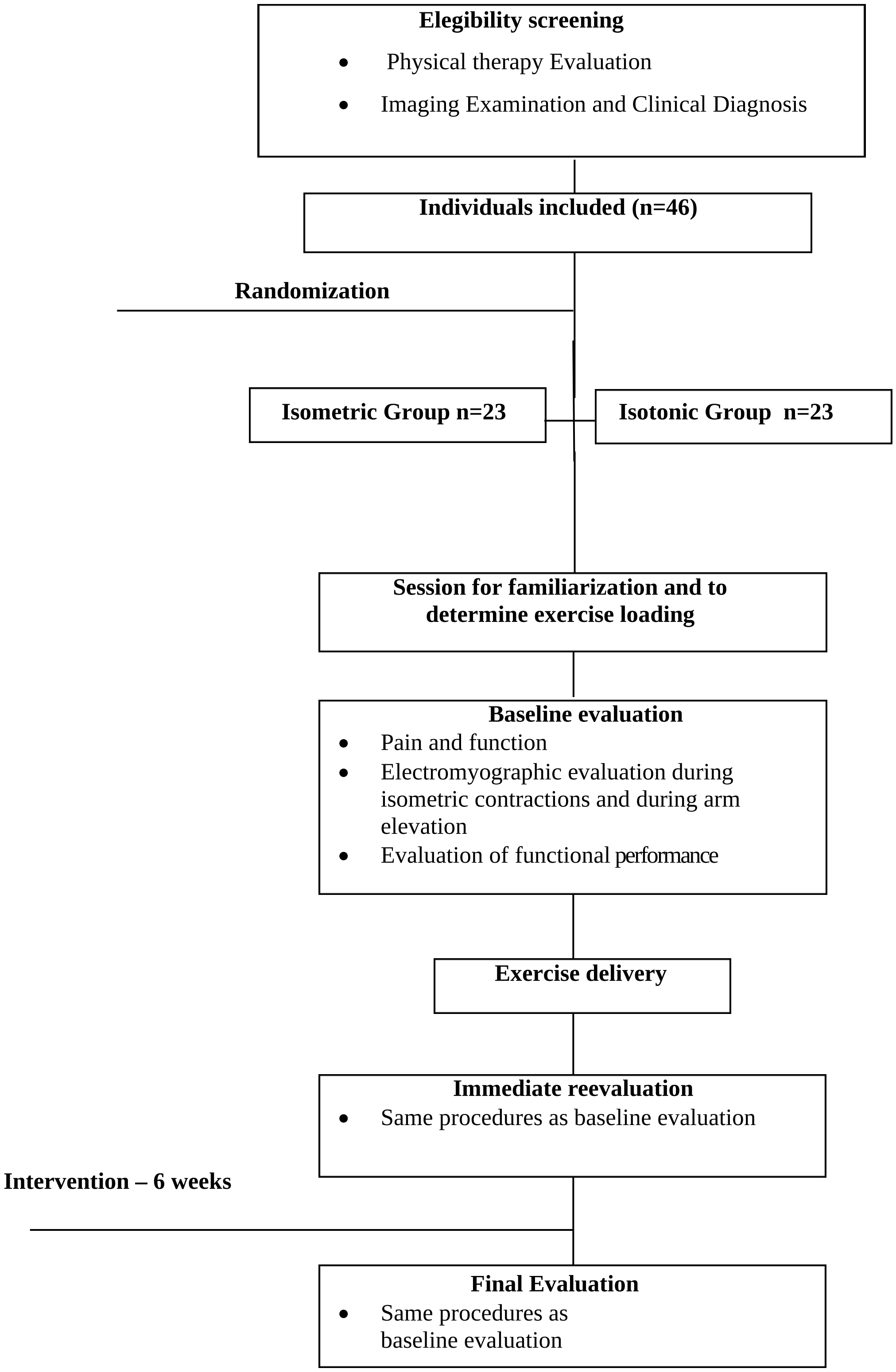

Figure 1. Study's flowchart.

5.4.1 Pain and function assessment

To assess shoulder pain and function, the Brazilian version of the Penn Shoulder Score (PSS) questionnaire will be used, which includes the domains of pain, satisfaction and function. The pain and satisfaction domains have, respectively, three items (pain at rest, pain during normal activities, and pain during strenuous activities) and one item (satisfaction with the current level of shoulder function) assessed using a numerical rating scale (NPRS) from 0 to 10, where 0 corresponds to no pain and not satisfied, while 10 corresponds to the worst possible pain and very satisfied. The function domain contains twenty items, graded on a four-point Likert scale, ranging from 0, which means “I can't do it at all”, to 3, “with no difficulty”, with a maximum score of 60 points. The PSS score ranges from 0 to 100 points, where the maximum score (100) indicates no pain, high satisfaction and good function (44).

In addition, aspects of quality of life will be evaluated through the Brazilian version of the Western Ontario Rotator Cuff Index (WORC) questionnaire, developed and validated for individuals with RC dysfunction, consisting of 21 items in five domains regarding life and health (physical symptoms, sport/recreation, work, lifestyle, and emotions). Each item has the same weight and varies from 0 to 100 (100 mm on a visual analogue scale - VAS), and the total of the questionnaire varies from 0 to 2100 mm, which are converted to a score of 100%, where 0% means the worst possible score and 100% implies no reduction in health-related quality of life (45).

5.4.2 Assessment of electromyographic activity during maximal isometric contraction and during arm elevation

The maximum isometric contractions will be performed with a dynamometer (Nextech, DFS-X1000, Nextech Global Company Limited, Thailand), which will be fixed in an apparatus that guarantees isometric contraction, and individuals will be instructed to push against the dynamometer for arm elevation and external and internal rotations as hard as possible. For arm elevation, individuals will be sitting in a chair with back support and shoulder to 90º of elevation in the scapular plane. For shoulder external and internal rotation, individuals will be sitting, shoulder positioned at 0º abduction, flexed elbow 90º

and fist in neutral position (34). Individuals will be verbally encouraged and maintain contraction for five seconds. Each test will be performed twice, with a two -minute interval between attempts, and the average values will be used in the analysis.

For arm elevation individuals will be standing and will perform arm elevation in the scapular plane (40º of humeral abduction) using a flat vertical surface as a reference. On this same surface will 30, 60, 90 and 120 degrees of elevation will be marked for each individual. After that, individuals will perform arm elevation in each of these angles with and without load, supporting a weight of 1.5 kg for those with less than 68 kg body weight and a weight of 2.5 kg for those who with more than 68.1 kg body weight (13).

During the maximum isometric contractions and arm elevation in the different angles with and without load, the electromyographic signal of the lower trapezius, serratus anterior, middle deltoid, and infraspinatus will be collected.

For the acquisition and processing of electromyographic signals, a signal an 8 channels (EMG System do Brasil®) conditioner module (MSC 1000) with digital analog converter - A/D (CAD, 12/36-60 K) with 12 bits resolution will be used. The equipment has a common rejection ratio (RMC) greater than 80 dB, with 2,000 Hz sampling frequency and the filtrate signal between 20 and 500 Hz. As the gain programmed in the converter is 50 times and 20 times in the electrodes, the signals will be amplified 1000 times. The electromyographer will be fed by a battery and connected to a notebook that will receive the signal and store it in file. EMGLAB (EMG System from Brazil®, Brazil) software will be used for digital signal analysis.

Active surface electrodes composed of an Ag/AgCl system associated with a conductive gel, simple differential, of bipolar configuration with dimensions of 4 cm X 2.2 cm of adhesive area and 1 cm of conductive area separated by 2 cm between electrode will be used (Miotec®, Brazil). A reference electrode of monopolar configuration will also be used, composed of an Ag/AgCl system associated with a conductive gel, with a diameter of 3.8 cm of adhesive area and 1 cm of conductive area (Miotec®, USA).

The positioning of sensors for the evaluation of lower trapezius, serratus anterior, middle deltoid and infraspinatus will follow the procedure described in

Michener et al. (46). A reference electrode will be attached to the ulnar styloid process on the contralateral side (48). Before placing the electrodes, the skin of the assessed regions will be shaved and cleaned with alcohol. The electromyographic activity will be evaluated only in the muscles of the symptomatic side, and after the tests, the individuals will be questioned about their pain, using the Numerical Pain Rating Scale (0-10, with 0 being no pain and 10 being the worst possible pain). (49).

To evaluate the amplitude of the electromyographic signal of each muscle and subsequently measure the muscle activation ratio between muscle pairs, the raw data will be filtered at a bandpass frequency of 20-450 Hz, rectified and smoothed using an algorithm of root mean square (RMS) with a 50-millisecond moving window, using Matlab software.

The ratio of muscle activation between the following pairs will be evaluated: 1) infraspinatus and middle deltoid; 2) infraspinatus and lower trapezius; and 3) lower trapezius and serratus anterior. These muscle pairs were selected since individuals with RC dysfunction have increased compensatory activity of the middle portion of the deltoid (14–16) and incoordination between the lower trapezius and serratus anterior and between the upper and lower portions of the trapezius (13).

5.4.3 Functional performance assessment

Three functional tests, which simulate activities of daily living will be performed. During the three tests, the individuals will be standing with their arms at their sides and will start each of the tests with their arms at their sides and their hands on the side of their thighs.

1. Reach a point above the head: the individuals will be positioned 30 cm away from a wall, which will have a target 10% higher than the individual's height. During the test, subjects will repeatedly reach the target point on the wall, perform a sagittal arm raise, and return to the starting position.

2. Reach Back of Head: Subjects will reach the back of head (occipital region) and return to starting position. Subjects will not be allowed to tilt the trunk or head during the test and the elbow must be aligned laterally with the head while subjects will touch the occipital region.

3. Reaching for the opposite shoulder blade or back: Subjects will reach for their opposite shoulder blade, or attempt to reach a point farther behind their back, and return to the starting position. Subjects will not be allowed to tilt or rotate their trunk or head during the test.

Each of the tests will be performed 20 times and as quickly as possible, once on each side. The time to complete the task will be measured with a stopwatch.

5.5 Intervention Protocol

Individuals from each group will perform an exercise session according to their group (isometric and isotonic) on the day of baseline assessment in order to verify the immediate effects of exercises. Then, they will participate in a six-week intervention, twice a week, in order to verify the effects of a strengthening program directed at the RC. Exercises will be performed on both limbs.

Individuals from all groups will undergo a protocol for stretching and strengthening the periscapular musculature following the protocol used by Camargo et al (53), which consists of stretching the upper trapezius and pectoralis minor muscles and the posterior portion of the shoulder, and strengthening the serratus anterior and lower trapezius muscles.

The evaluation to determine the initial loads of the exercises will be carried out one week before the baseline evaluation and beginning of the interventions. At that moment, participants will also familiarize themselves with each exercise with light resistance and will be guided by the evaluator to perform the technique correctly. In order to adjust and progress the exercise load over the six weeks of intervention, the assessments will be repeated in the third and fifth weeks of intervention, as shown in Figure 2 with the flowchart of the intervention procedures and progression of each group.

5.5.1 Stretching and strengthening of the periscapular muscles

Both groups will perform stretching and strengthening protocol proposed by Camargo et al (53). Stretching of the upper trapezius will be performed actively with the individual performing lateral flexion of the neck, in both sides; the pectoralis minor stretching will be performed with the individual facing two walls

with a 90º angle between them, with shoulder elevation in the scapular plane of 90º and elbow flexion at 90º, and the individual will be instructed to project the body forward as far as they can. Posterior shoulder stretching will be performed with the horizontal adduction of the shoulder, with the individuals supported on the wall in order to avoid stabilizing the scapula (54). 3 sets of 30 seconds will be performed for each stretch, with an interval of 30 seconds between repetitions (53).

To strengthen the serratus anterior, the scapula protraction exercise will be performed in supine with shoulder flexed at 90º; and to strengthen the lower trapezius muscle, shoulder extension exercises will be performed with the elbow extended and the individual in prone position. For strengthening, 3 sets of 10 repetitions will be performed with 1 minute rest. Exercise resistance will be offered by means of elastic bands with progression determined by changing the colors of the bands, according to the individual's perception, so that as soon as the exercise is performed without difficulty and fatigue, the elastic band will be replaced by one with greater resistance (53).

5.5.2 Isometric Exercise Group

Isometric exercises for RC muscles will consist of 3 repetitions of 32 seconds at an intensity of 70% of the maximum isometric strength for each exercise, according to the protocol adapted from Rio et al. (27,28), and with an interval between repetitions of 80 seconds (26). The evaluation of maximum strength as well as the performance of the exercises will be performed with a manual dynamometer coupled to a rigid bulkhead and attached to a fixed wooden column. The dynamometer has a function which allows real-time monitoring of the developed force, allowing a visual feedback that will show the individuals the maintenance of the determined load during the exercise.

The determination of maximum isometric strength for the supraspinatus and internal and external rotator muscles will be performed during the shoulder strength assessment described above. The positioning of the individuals to perform the isometric exercises for these muscle groups will be the same as in the evaluation. Elevation of the shoulder in the plane of the scapula at 90º was adopted because it presents a lower risk of compression of the supraspinatus in the coracoacromial arch and, consequently, generates less pain and additional

injuries than exercises at lower arm elevation angles (55). The elevation movement should be performed with the thumb facing upwards, as the supraspinatus muscle is more selective compared to the middle and posterior deltoids (56).

In order to carry out the exercises, the individuals will be instructed to push the dynamometer in the direction of the desired movement and follow the sound feedback of the equipment, pre-adjusted to 70% of the maximum force, and the researcher responsible for the training will give verbal feedback so that the individuals avoid change of positioning or compensation and to avoid excessive contraction of the upper trapezius during the supraspinatus exercise.

5.5.3 Isotonic Exercise Group

Isotonic exercises for strengthening RC muscles in a concentric and eccentric way will be performed using dumbbells. The exercises will consist of 3 sets of 8 repetitions of each exercise at an intensity of 8 RM, with load reassessment in the third and fifth week of intervention, according to the protocol adapted from Kongsgaard et al. (24). The rest interval between sets of exercises will be 80 seconds (28).

The external rotation exercise will be performed in side lying on the contralateral shoulder, with shoulder at 0º of abduction and flexion and elbow flexion at 90º with a towel between the lower part of the arm and the trunk of the individuals. For internal rotation, the patient will be positioned in side lying on the limb to be strengthened with the shoulder at 0º of abduction and flexion and elbow flexion at 90º (22). The participants will be instructed to perform the concentric and eccentric exercises at a pace of 3 seconds for each contraction mode, which will be controlled by a metronome that will also serve as feedback to the participants (57).

To strengthen the supraspinatus, an arm elevation will be performed in scapular plane with a neutral wrist and thumb upwards, in a range of 60 to 90º, since in this range there is less risk of compression of the supraspinatus in the coracoacromial arch and, consequently, less risk of increasing pain and generating additional injuries than at lower arm elevation angles (55).

5.6 Ethical aspects

The study will be submitted to evaluation by the Ethics Committee for Research on Human Beings and will respect the guidelines and norms contained in Resolution 466/12 of the National Health Council, which concerns ethics in carrying out research with human beings, advocating discretion and respect for research participants throughout the data collection, and must guarantee their duties and rights. All users must voluntarily assent their participation, which will be made explicit by signing two copies of the Informed Consent Form and the Authorization Term for Image Use.

The risks inherent to this research are minimal and the presence of some painful discomfort may occur during the evaluation and intervention procedures. Thus, individuals will have the right to withdraw from the research at any time they wish and the responsible researcher will have the duty to solve all their doubts and guarantee the privacy and confidentiality of the research participant, always having to be transparent about the procedures that will be carried out. If there is any physical damage, assistance and physiotherapy will be provided to the volunteer at the school clinics in the respective areas.

As for the promoted benefits, the participant will be contributing to the scientific community, as he will encourage an evidence-based physiotherapeutic practice. In addition, they will receive a complete and detailed assessment of the kinetic and functional conditions of their shoulder.

5.7 Statistical analysis

Data will be analyzed statistically in a descriptive and inferential way. Mean and standard deviation will be calculated for all demographics and dependent variables. Regarding inferential analyses, initially, the Shapiro-Wilk Normality Test will be used to classify the distribution of each variable.

In order to perform the comparison between the two groups (isometric and isotonic) and between the evaluations, for each dependent variable, we will apply two-way ANOVA with repeated measures when the data are considered parametric and Friedman when the data are non-parametric.

When necessary, post hoc Tukey or Duncan tests will be applied, respectively. All tests will be performed using the Statistical Package for Social Sciences – SPSS, version 20.0 software, and the significance level adopted will be $\alpha \leq 0,05$.

6 Outcome and Expected Results

Rotator cuff tendinopathy is a very common injury in general population and training through progressive resistance exercises has been one of the main alternatives for conservative treatment. However, there is still no evidence in the literature of the effects and benefits of different types of resistance exercises, such as isometric, concentric and eccentric exercises.

Thus, through the results obtained, this project will contribute to the advancement of scientific knowledge in the field of assessment and rehabilitation of the shoulder complex, specifically in RC dysfunction. Knowledge about the effects of isometric exercise applied to RC tendinopathy on pain, function and neuromuscular control involved in the shoulder complex is clinically relevant, as it may support the physiotherapeutic decision-making process in the treatment of RC tendinopathy. The activities that will be carried out during this project will enable important advance in the development of specialized knowledge and approach to the patient with disorders of the shoulder complex. It is important to emphasize that the development of projects aimed at evaluating and developing intervention protocols in upper limb disorders is very important, since despite the high incidence of shoulder disorders in the general population, there are few researchers working in this field of research in Brazil. Thus, this project can contribute by helping to train human resources to produce scientific knowledge in this area, which is so important for Physiotherapy.

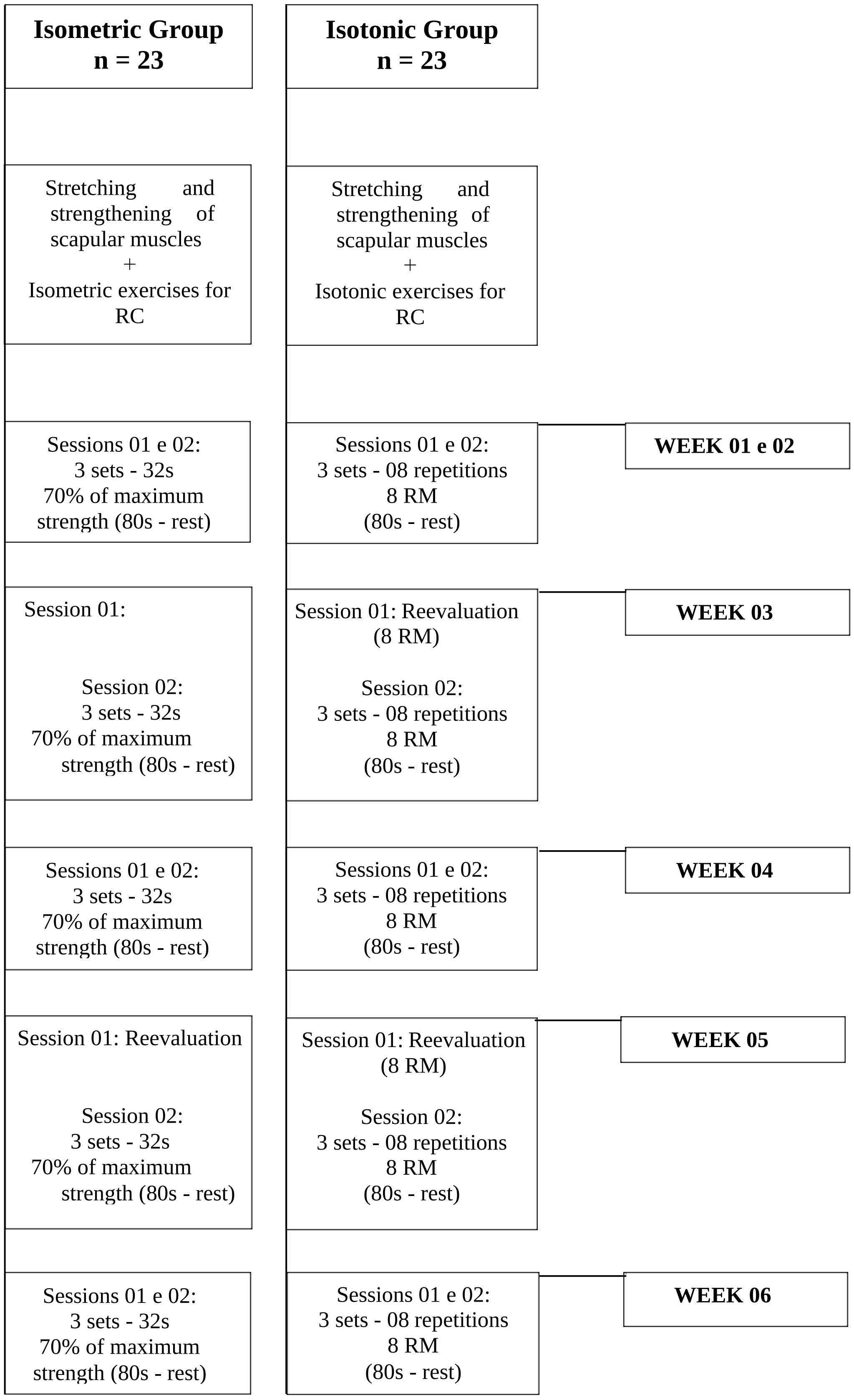

Figure 2. Flow chart of intervention protocol and progression.

7. Timeline

Goals and activities	2019											
	Jan	Feb	Mar	Apr	May	Jun	Jul	Aug	Sep	Oct	Nov	Dec
Submission of the project to CEP				X	X							
Registration of the project on the Brazilian Registry Clinical Trials					X	X						
Training of evaluators with kinematics equipment						X						
Subject recruitment and data collection						X	X	X	X	X	X	X
Data processing and analysis											X	X
Bibliographic Update					X	X	X	X	X	X	X	X
	2020											
Subject recruitment and data collection	X	X	X	X	X							
Data processing and analysis						X	X	X				
Discussion and partial presentation of results in scientific events			X	X	X	X	X	X	X	X	X	X
Bibliographic Update	X	X	X	X	X	X	X	X	X	X	X	X
	2021											
Bibliographic Update	X	X	X	X	X	X	X					
Elaboration, submission, review and publication of manuscripts	X	X	X	X	X	X	X					
Public defense of the Doctoral Dissertation								X				
	2022											
Bibliographic Update	X	X	X	X	X	X	X	X	X	X	X	X
	2023											
Bibliographic Update	X	X	X	X	X	X	X	X	X	X	X	X
Subject recruitment and data collection – after amendment approval by CEP	X	X	X	X	X	X	X	X	X			
Data processing and analysis				X	X	X	X	X	X	X	X	X
Elaboration, submission, review and publication of manuscripts								X	X	X	X	X
	2024											
Bibliographic Update	X	X										
Elaboration, submission, review and publication of manuscripts	X	X										
Public defense of the Doctoral Dissertation		X										

8. Detailed and justified budget

Costing Items		
10 packs of 100 disposable dual electrodes (Double Trace, Shanghai Litu Medical Appliances Co., Ltda, China)	Double electrodes for capturing electromyographic signal (Double Trace da Shanghai Litu Medical Appliances Co., Ltda, China).	R$1.800,00
Stationery supplies such as A4 legal paper, printouts, copies, pens and printer cartridges	This material will be used to print the evaluation sheets used in the research and the informed consent.	R$ 400,00
**Total - costing**		**R$2.200,00**

References

1. SERGIENKO S, KALICHMAN L. Myofascial origin of shoulder pain: A literature review. **J Bodyw Mov Ther.** v.19, n. 1, p. 91-101, 2015.

2. LIN JJ, HANTEN WP, OLSON SL, RODDEY TS, SOTO-QUIJANO DA, LIM HK, et al. Functional activity characteristics of individuals with shoulder dysfunctions. J **Electromyogr Kinesiol**. v. 15, n. 6, p. 576-86, 2005.

3. LEWIS J, MCCREESH K, ROY J-S, GINN K. Rotator Cuff Tendinopathy: Navigating the Diagnosis-Management Conundrum. **J Orthop Sport Phys Ther**. v. 45, n. 11, p. 923-37, 2015.

4. LUDEWIG PM, REYNOLDS JF. The Association of Scapular Kinematics and Glenohumeral Joint Pathologies. **J Orthop Sport Phys Ther**. v. 39, n. 2, p. 90–104, 2009.

5. SEITZ AL, MCCLURE PW, FINUCANE S, BOARDMAN ND, MICHENER LA. Mechanisms of rotator cuff tendinopathy: Intrinsic, extrinsic, or both? **Clin Biomech**. v. 26, n. 1, p. 1-12, 2011.

6. CAMARGO PR. Eccentric training as a new approach for rotator cuff tendinopathy: Review and perspectives. **World J Orthop**. v. 5, n. 5, p. 634, 2014.

7. LUDEWIG PM, REYNOLDS JF. The Association of scapulair Kinematics and Glenohumeral Joint Pathologies. **J Orthop Sport Phys Ther**. v. 39, n. 2, p. 90-104, 2009.

8. TIMMONS MK, THIGPEN CA, SEITZ AL, KARDUNA AR, ARNOLD BL, MICHENER LA. Scapular Kinematics and Subacromial-Impingement Syndrome : A Meta-Analysis. p. 354-70, 2012.

9. PADKE V, CAMARGO PR, LUDEWIG PM. Scapular and rotator cuff muscle activity during arm elevation: A review of normal function and alterations with shoulder impingement. **Rev Bras Fisioter**. v. 13, n. 1, p. 1-9, 2009.

10. PAGE P. Shoulder muscle imbalance and subacromial impingement syndrome in overhead athletes. **Int J Sports Phys Ther**. v. 6, n. 1, p. 51-8, 2011.

11. DIEDERICHSEN LP, NØRREGAARD J, DYHRE-POULSEN P, WINTHER A, TUFEKOVIC G, BANDHOLM T, et al. The activity pattern of shoulder muscles in subjects with and without subacromial impingement. **J Electromyogr Kinesiol**. v. 19, n. 5, p. 789-99, 2009.

12. HUNG CJ, JAN MH, LIN YF, WANG TQ, LIN JJ. Scapular kinematics and impairment features for classifying patients with subacromial impingement syndrome. **Man Ther**. v. 15, n. 6, p. 547-51, 2010.

13. MICHENER LA, SHARMA S, COOLS AM, TIMMONS MK. Relative scapular muscle activity ratios are altered in subacromial pain syndrome**. J Shoulder Elb Surg.** v. 25, n. 11, p. 1861-7, 2016.

14. MYERS JB, HWANG JH, PASQUALE MR, BLACKBURN JT, LEPHART SM. Rotator cuff coactivation ratios in participants with subacromial impingement syndrome. **J Sci Med Sport**. v. 12, n. 6, p. 603-8, 2009.

15. DYRNA F, KUMAR NS, OBOPILWE E, SCHEIDERER B, COMER B, NOWAK M, et al. Relationship Between Deltoid and Rotator Cuff Muscles During Dynamic Shoulder Abduction: A Biomechanical Study of Rotator Cuff Tear Progression. **Am J Sports Med**. v. 46, n. 8, p. 1919-26, 2018.

16. MULLA DM, MCDONALD AC, KEIR PJ. Upper body kinematic and muscular variability in response to targeted rotator cuff fatigue. **Hum Mov Sci**. v. 59, n. 6, p. 121-33, 2018.

17. PADKE V, CAMARGO PR, LUDEWIG PM. Scapular and rotator cuff muscle activity during arm elevation: A review of normal function and alterations with shoulder impingement. **Rev Bras Fisioter**. v. 13, n. 1, p. 1-9, 2009.

18. TOLIOPOULOS P, DESMEULES F, BOUDREAULT J, ROY JS, FRÉMONT P, MACDERMID JC, et al. Efficacy of surgery for rotator cuff tendinopathy: a systematic review. **Clin Rheumatol**. 2014;33(10):1373–83.

19. WANG JHC, IOSIFIDIS MI, FU FH. Biomechanical basis for tendinopathy. Clin Orthop Relat Res. v. 44, n. 3, p. 320-32, 2006.

20. KADER D. Achilles tendinopathy: some aspects of basic science and clinical management. **Br J Sport Med**. v. 36, n.1, p. 239-49, 2002.

21. KUHN JE. Exercise in the treatment of rotator cuff impingement: A systematic review and a synthesized evidence-based rehabilitation protocol**. J Shoulder Elb Surg**. v. 18, n. 1, p. 138-60, 2009.

22. BLUME C, WANG-PRICE S, TRUDELLE-JACKSON E, ORTIZ A. Comparison of Eccentric and Concentric Exercise Interventions in Adults With Subacromial Impingement Syndrome. **Int J Sports Phys Ther.** v. 10, n. 4, p. 441-55, 2015.

23. LEWIS J. Rotator cuff related shoulder pain: Assessment, management and uncertainties. **Man Ther**. v. 23, p. 57-68, 2016.

24. KONGSGAARD M, KOVANEN V, AAGAARD P, DOESSING S, HANSEN P, LAURSEN AH, et al. Corticosteroid injections, eccentric decline squat training and heavy slow resistance training in patellar tendinopathy. **Scand J Med Sci Sport**. v. 19, n. 6, p. 790-802, 2009.

25. BEYER R, KONGSGAARD M, HOUGS KJÆR B, ØHLENSCHLÆGER T, KJÆR M, MAGNUSSON SP. Heavy slow resistance versus eccentric training as treatment for achilles tendinopathy: A randomized controlled trial. **Am J Sports Med**. v. 43, n. 7, p. 1704-11, 2015.

26. PEARSON SJ, STADLER S, MENZ H, MORRISSEY D, SCOTT I, MUNTEANU S, et al. Immediate and Short- Term Effects of Short-and Long-Duration Isometric Contractions in Patellar Tendinopathy. v. 00, n. 00, p. 1-6, 2018.

27. RIO E, KIDGELL D, PURDAM C, GAIDA J, MOSELEY GL, PEARCE AJ, et al. Isometric exercise induces analgesia and reduces inhibition in patellar tendinopathy. **Br J Sports Med.** v. 49, n. 19, p. 1277-83, 2015.

28. RIO E, HONS BAP, PHYS M, ARK M VAN, DOCKING S, HONS B, et al. Isometric Contractions Are More Analgesic Than Isotonic Contractions for Patellar Tendon Pain : An In-Season Randomized Clinical Trial. v. 0, n. 0, p. 1-7, 2016.

29. NAUGLE KM, NAUGLE KE, FILLINGIM RB, RILEY JL. Isometric Exercise as a Test of Pain Modulation: Effects of Experimental Pain Test, Psychological Variables, and Sex. **Pain Med**. v. 15, n. 4, p. 692-701, 2014.

30. PARLE PJ, RIDDIFORD-HARLAND DL, HOWITT CD, LEWIS JS. Acute rotator cuff tendinopathy: Does ice, low load isometric exercise, or a combination of the two produce an analgaesic effect? **Br J Sports Med**. v. 51, n. 3, p. 208-9, 2017.

31. MINAGAWA H, YAMAMOTO N, ABE H, FUKUDA M, SEKI N, KIKUCHI K, et al. Prevalence of symptomatic and asymptomatic rotator cuff tears in the general population: From mass- screening in one village. **J Orthop**. v. 10, n. 1, p. 8-12, 2013

32. TASHJIAN RZ. Epidemiology, Natural History, and Indications for Treatment of Rotator Cuff Tears. **Clin Sports Med**. v. 31, n. 4, p. 589-604, 2012.

33. MACDERMID JC, SILBERNAGEL KG. Outcome Evaluation in Tendinopathy: Foundations of Assessment and a Summary of Selected Measures. **J Orthop Sport Phys Ther.** v. 45, n. 11, p. 950-64, 2015.

34. CHIEN CW, BAGRAITH KS, KHAN A, DEEN M, STRONG J. Comparative responsiveness of verbal and numerical rating scales to measure pain intensity in patients with chronic pain. **J Pain**. v. 14, n. 12, p. 1653–62, 2013.

35. COOLS AM, CAMBIER D, WITVROUW EE. Screening the athlete's shoulder for impingement symptoms: A clinical reasoning algorithm for early detection of shoulder pathology. **Br J Sports Med**. v. 42, n. 8, p. 628-35, 2008.

36. MICHENER LA, WALSWORTH MK, DOUKAS WC, MURPHY KP. Reliability and Diagnostic Accuracy of Physical Examination Tests and Combination of Tests for Subacromial Impingement. **Arch Phys Med Rehabil**. v, 90, n. 11, p. 1898-903, 2009.

37. NAREDO E. Painful shoulder: comparison of physical examination and ultrasonographic findings. Ann Rheum Dis. v. 61, n. 2, p. 132-6, 2002.

38. WALMSLEY S, RIVETT DA, OSMOTHERLY PG. Adhesive capsulitis: establishing consensus on clinical identifiers for stage 1 using the DELPHI technique. **Phys Ther**. v. 89, n. 9, p. 906-17, 2009.

39. ALBURQUERQUE-SENDÍN F, CAMARGO P, VIEIRA A, SALVINI T. Bilateral myofascial trigger points and pressure pain thresholds in the shoulder muscles in patients with unilateral shoulder impingement syndrome: a blinded, controlled study. **Clin J Pain**. v. 29, n. 6, p. 478-86, 2013.

40. MCCLURE PW, MICHENER L A, KARDUNA AR. Shoulder function and 3-dimensional scapular kinematics in people with and without shoulder impingement syndrome. **Phys Ther**. v. 86, n. 8, p. 1075-90, 2006.

41. SANTAMATO A, SOLFRIZZI V, PANZA F, TONDI G, FRISARDI V, LEGGIN BG, et al. Short-term effects of high-intensity laser therapy versus ultrasound therapy in the treatment of people with subacromial impingement syndrome: a randomized clinical trial. **Phys Ther**. v. 89, n. 7, p. 643-52, 2009.

42. LEWIS T, COOK J. Fluoroquinolones and tendinopathy: A guide for athletes and sports clinicians and a systematic review of the literature. **J Athl Train.** v. 49, n. 3, p.422–7, 2014.

43. LUI PPY. Tendinopathy in diabetes mellitus patients—Epidemiology, pathogenesis, and management. **Scand J Med Sci Sport.** V. 27, n. 8, p. 776–87, 2017.

44. Napoles BV, Hoffman CB, Martins J, Oliveira AS De. Tradução e adaptação cultural do Penn Shoulder Score para a Língua Portuguesa: PSS-Brasil. Rev Bras Reumatol. v. 50, n. 4, p. 389-97, 2010.

45. LOPES AD, CICONELLI RM, CARRERA EF, GRIFFIN S, FALOPPA F, DOS REIS FB. Validity and reliability of the Western Ontario Rotator Cuff Index (WORC) for use in Brazil. **Clin J Sport Med**. v. 18, n. 3, p. 266-72, 2008.

46. Michener LA, Elmore KA, Darter BJ, Timmons MK. Biomechanical measures in participants with shoulder pain: Intra-rater reliability. Man Ther. 2016; 22:86–93.

47. HERMENS HJ, FRERIKS B, MERLETTI R, ET AL. Project SENIAM (Surface Electromyography for the Non-Invasive Assessment of Muscles). European Recommendations for Surface Electromyography. 1999.

48. SOUSA C DE O, MICHENER LA, RIBEIRO IL, REIFF RB DE M, CAMARGO PR, SALVINI TF. Motion of the shoulder complex in individuals with isolated acromioclavicular osteoarthritis and associated with rotator cuff dysfunction: Part 2 - Muscle activity. **J Electromyogr Kinesiol**. v. 25, n. 1, p. 77-83, 2015.

49. PUGA VODO, LOPES AD, SHIWA SR, ALOUCHE SR, COSTA LOP. Clinimetric Testing Supports the Use of 5 Questionnaires Adapted Into Brazilian Portuguese for Patients With Shoulder Disorders. **J Orthop Sport Phys Ther**. v. 43, n. 6, p. 404-13, 2013.

50. LUDEWIG PM, PHADKE V, BRAMAN JP, HASSETT DR, CIEMINSKI CJ, LAPRADE RF. Motion of the shoulder complex during multiplanar humeral elevation. **J Bone Jt Surg**. v. 91, n. 2, p. 378-89, 2009.

51. WU G, VAN DER HELM FCT, VEEGER HEJ, MAKHSOUS M, VAN ROY P, ANGLIN C, et al. ISB recommendation on definitions of joint coordinate systems of various joints for the reporting of human joint motion - Part II: Shoulder, elbow, wrist and hand. **J Biomech**. v. 38, n. 5, p. 981-92, 2005.

52. TATE AR, MCCLURE P, KAREHA S, IRWIN D, BARBE MF. A clinical method for identifying scapular dyskinesis, part 2: Validity**. J Athl Train.** v. 44, n. 2, p. 165-73, 2009.

53. CAMARGO PR, ALBURQUERQUE-SENDÍN F, AVILA MA, HAIK MN, VIEIRA A, SALVINI TF. Effects of Stretching and Strengthening Exercises, With and Without Manual Therapy, on Scapular Kinematics, Function, and Pain in Individuals With Shoulder Impingement: A Randomized Controlled Trial. **J Orthop Sport Phys Ther**. v. 45, n. 12, p. 984-97, 2015.

54. SALAMH PA, KOLBER MJ, HANNEY WJ. Effect of scapular stabilization during horizontal adduction stretching on passive internal rotation and posterior shoulder tightness in young women volleyball athletes: A randomized controlled trial. **Arch Phys Med Rehabil**. v. 96, n. 2, p. 349-56, 2015.

55. LAWRENCE RL, SCHLANGEN DM, SCHNEIDER KA, SCHOENECKER J,

SENGER AL, STARR WC, et al. Effect of glenohumeral elevation on subacromial supraspinatus compression risk during simulated reaching. **J Orthop Res.** v. 35, n. 10, p. 2329-37, 2017.

56. REINOLD MM, MACRINA LC, WILK KE, FLEISIG GS, DUN S, BARRENTINE SW, et al. Electromyographic analysis of the supraspinatus and deltoid muscles during 3 common rehabilitation exercises. **J Athl Train.** v. 42, n. 4, p. 464-9, 2007.

57. COOLS AMJ, VANDERSTUKKEN F, VEREECKEN F, DUPREZ M, HEYMAN K, GOETHALS N, et al. Eccentric and isometric shoulder rotator cuff strength testing using a hand-held dynamometer: reference values for overhead athletes. **Knee Surgery, Sport Traumatol Arthrosc.** v. 24, n. 12, p. 3838-47, 2016.

ATTACHMENTS

Anexo I. *Penn Shoulder Score* (PSS)

IDENTIFICAÇÃO DO PACIENTE

Nome completo: . . . . Registro: . . . .

Data de nascimento: . . . . / . . . . / . . . . Idade: . . . . Sexo: ( ) F ( ) M

Profissão: . . . . Tel: . . . . Dominância: ( ) D ( ) E

Hipótese diagnóstica: . . . .

Cirurgia: Qual: . . . . Quando: . . . . / . . . . / . . . .

Braço com dor ou disfunção: ( ) D ( ) E ( ) Ambos Pior braço: ( ) D ( ) E

Há quanto tempo tem dor ou disfunção nesse braço: . . . .

PENN SHOULDER SCORE (PSS-BRASIL)

Nome: . . . . Braço avaliado: . . . . Data: . . . . . / . . . . . / . . . . .

PONTUAÇÃO PSS-BRASIL PARA O OMBRO

Parte I: Dor e Satisfação: Por favor, indique o número que mais se aproxima do seu nível de dor ou satisfação.

											Uso Exclusivo
Dor em repouso, com o braço parado ao lado do corpo:											
0	1	2	3	4	5	6	7	8	9	10	. . . .
sem dor										pior dor possível	(10 – Nº circulado)
Dor durante atividades normais (comer, vestir-se, banhar-se):											
0	1	2	3	4	5	6	7	8	9	10	. . . . (10 – Nº circulado)
sem dor										pior dor possível	(marcar 0 se não se aplica)
Dor durante atividades de esforço (alcançar, levantar, empurrar, puxar, jogar um objeto):											
0	1	2	3	4	5	6	7	8	9	10	. . . . (10 – Nº circulado)
sem dor										pior dor possível	(marcar 0 se não se aplica)
										**Pontuação para dor =**	. . . . / 30
Qual a sua satisfação com o nível atual de função do seu ombro?											
0	1	2	3	4	5	6	7	8	9	10	. . . . / 10
não satisfeito										muito satisfeito	(Nº circulado)

PONTUAÇÃO PSS-BRASIL

Parte II: Função: Por favor, indique o nível de dificuldade que você pode ter ao realizar cada atividade.

		Já não realizava antes da lesão	Sem dificuldade	Alguma dificuldade	Muita dificuldade	Não consegue de forma alguma
1	Alcançar a parte inferior da sua coluna com a mão do braço afetado, para arrumar a camisa dentro das calças.	X	3	2	1	0
2	Lavar o meio das costas ou prender o sutiã pelas costas com o braço afetado.	X	3	2	1	0
3	Realizar atividades de higiene pessoal com o braço afetado.	X	3	2	1	0
4	Lavar a parte posterior do ombro oposto com o braço afetado.	X	3	2	1	0
5	Pentear os cabelos com o braço afetado.	X	3	2	1	0
6	Colocar a mão do braço afetado atrás da cabeça com o cotovelo para fora e para o lado.	X	3	2	1	0
7	Vestir-se (incluindo vestir casaco e tirar a blusa pela cabeça).	X	3	2	1	0
8	Dormir em cima do lado afetado.	X	3	2	1	0
9	Abrir/empurrar a porta com o braço afetado.	X	3	2	1	0
10	Carregar um livro ou pasta, junto ao corpo, com o braço afetado.	X	3	2	1	0
11	Carregar uma sacola de compras ou maleta com o braço afetado.	X	3	2	1	0
12	Colocar uma lata (500 g a 1 kg) em uma prateleira à altura do ombro com o braço afetado esticado.	X	3	2	1	0
13	Colocar um pote de aproximadamente 5 kg (saco grande de arroz) em uma prateleira à altura do ombro com o braço afetado esticado.	X	3	2	1	0
14	Alcançar uma prateleira acima da cabeça com o braço afetado esticado.	X	3	2	1	0
15	Colocar uma lata (500 g a 1 kg) em uma prateleira acima da cabeça com o braço afetado esticado.	X	3	2	1	0
16	Colocar um pote de aproximadamente 5 kg (saco grande de arroz) em uma prateleira acima da cabeça com o braço afetado esticado.	X	3	2	1	0
17	Praticar atividades de lazer regulares ou esportes.	X	3	2	1	0
18	Realizar as tarefas de casa (limpar, lavar a roupa, cozinhar).	X	3	2	1	0
19	Arremessar acima do ombro/ nadar/ esportes com raquete, com o braço afetado. (Circule as atividades que se aplicam ao paciente)	X	3	2	1	0
20	Trabalhar o tempo todo em seu emprego ou função usual.	X	3	2	1	0

PONTUAÇÃO PARA FUNÇÃO

Total de colunas = ... (a)

Número de "X" x 3 = ... (b), 60 - . . . . (b) = ... (c)

(se nenhum X for circulado, a pontuação da função = número total de colunas)

Pontuação da função = ... (a) ÷ . . . . (c) = ... x 60 . . . . /60

Pontuação total (Parte I e II) =

Anexo II. ***Western Ontario Rotator Cuff Index*** **(WORC)**

INSTRUÇÕES AOS PACIENTES

As perguntas deste questionário possuem o formato abaixo. Você deverá indicar sua resposta colocando uma barra "/" na linha horizontal de acordo com a explicação seguinte:

OBSERVE:

1. Se você colocar uma barra "/" à esquerda, no final da linha, isto é:

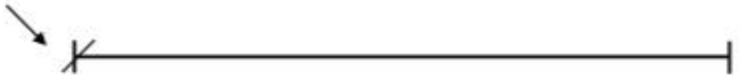

Então, você está indicando que não tem dor.

2. Se você colocar uma barra "/" à direita, no final da linha, isto é:

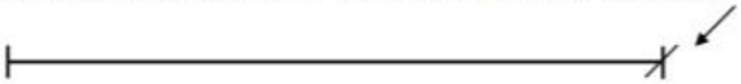

Então, você está indicando que sua dor é extrema.

Por favor, preste atenção:

a) quanto mais à direita você colocar a barra "/", **mais** você apresentará aquele sintoma.

b) quanto mais à esquerda você colocar a barra "/", **menos** você apresentará aquele sintoma.

c) Não coloque sua barra "/" fora dos marcadores finais.

Você deverá indicar no questionário a intensidade do sintoma que você sentiu nesta última semana com relação a seu ombro afetado. Se você não tiver certeza sobre o ombro que está envolvido ou se você tiver quaisquer outras dúvidas, pergunte antes de preencher o questionário.

Se, por algum motivo, você não entender uma pergunta, procure as explicações que estão incluídas no final desse questionário.

Se um item não se relacionar a você ou se você não o tiver sentido nesta última semana, imagine qual seria sua resposta mais adequada para tal.

Seção A: Sintomas Físicos
INSTRUÇÕES AOS PACIENTES

As perguntas abaixo relacionam aos sintomas físicos que você apresentou por causa do problema do seu ombro. Em todos os casos, por favor, indique a quantidade de sintomas que você apresentou nesta última semana. (Por favor, assinale a sua resposta com uma barra "/").

1. Quanta dor aguda você sente no seu ombro?

dor sem dor |————————————| extrema

2. Quanta dor constante, incômoda, você sente no seu ombro?

sem dor dor |————————————| extrema

3. Quanta fraqueza você sente no seu ombro ?

sem fraqueza |————————————| fraqueza extrema

4. Quanto você sente seu ombro endurecido ou travado?

Nada |————————————| extremamente endurecido

5. Quanto você se sente incomodado quando seu ombro estala, range ou crepita?

nenhum incomodo |————————————| extremamente incomodado

6. Quanto desconforto você sente nos músculos do seu pescoço por causa do seu ombro?

sem desconforto |————————————| extremo desconforto

Seção B: Esportes/Recreação
INSTRUÇÕES AOS PACIENTES

A seção a seguir questiona como o problema do seu ombro afetou suas atividades esportivas ou de lazer nesta última semana. (Por favor, para cada pergunta assinale sua resposta através de uma barra "/").

7. Quanto o seu ombro afetou seu nível de desempenho físico?

não afetou |————————————| extremamente afetado

8. Quanto o seu ombro afetou sua habilidade de arremessar com força ou à distância?

não afetou |————————————| extremamente afetado

9. Quanto medo você tem de que alguém ou alguma coisa esbarre no seu ombro afetado?

nenhum |————————————| medo extremo

10. Quanta dificuldade você sente quando faz "flexão de braços" ou outros exercícios pesados por causa do seu ombro?

sem dificuldade |————————————| extrema dificuldade

Seção C: Trabalho
INSTRUÇÕES AOS PACIENTES

A seção abaixo questiona quanto o problema do seu ombro afetou o seu trabalho em casa e fora de casa. (Por favor, indique a quantidade apropriada nesta última semana com uma barra "/").

11. Quanta dificuldade você sente na execução das atividades diárias em casa ou nas áreas externas dela (ex: jardim, quintal)?

nenhuma

dificuldade dificuldade |————————————————| extrema

12. Quanta dificuldade você sente para desempenhar tarefas acima do nível de sua cabeça?

nenhuma dificuldade |————————————————| extrema dificuldade

13. Quanto você usa seu braço bom para substituir seu braço machucado?

Não uso |————————————————| constantemente

14. Quanta dificuldade você tem para levantar objetos pesados na altura ou abaixo da altura do seu ombro?

sem dificuldade |————————————————| extrema dificuldade

Seção D: Estilo de vida
INSTRUÇÕES AOS PACIENTES

A seção seguinte questiona quanto o seu problema do ombro afetou ou mudou seu estilo de vida (Novamente, indique a quantidade apropriada nesta última semana com uma barra "/").

15. Quanta dificuldade você tem para dormir por causa do seu ombro?

Sem dificuldade |——————————| extrema dificuldade

16. Quanto desconforto você sente para arrumar o seu cabelo por causa do seu ombro?

nenhum desconforto |——————————| extremo desconforto

17. Quanta dificuldade você tem para brincar/rolar no chão com familiares ou amigos?

nenhuma dificuldade |——————————| extrema dificuldade

18. Quanta dificuldade você tem para se vestir ou se despir?

Sem dificuldade |——————————| extrema dificuldade

Seção E: Emoções
INSTRUÇÕES AOS PACIENTES

As perguntas abaixo questionam como você se sentiu nesta última semana com relação ao problema do seu ombro (Por favor, indique sua resposta com uma barra "/").

19. Quanta frustração você sente por causa do seu ombro?

nenhuma frustração ├──────────┤ extrema frustração

20. Quanto você se sente triste ou deprimido por causa do seu ombro?

nada extremamente ├──────────┤

21. Quanto você se sente preocupado com relação aos efeitos do seu ombro na sua ocupação ou trabalho?

não me sinto extremamente preocupado preocupado ├──────────┤

OBRIGADO POR COMPLETAR O QUESTIONÁRIO

APPENDICES

Appendice A. Research flyers

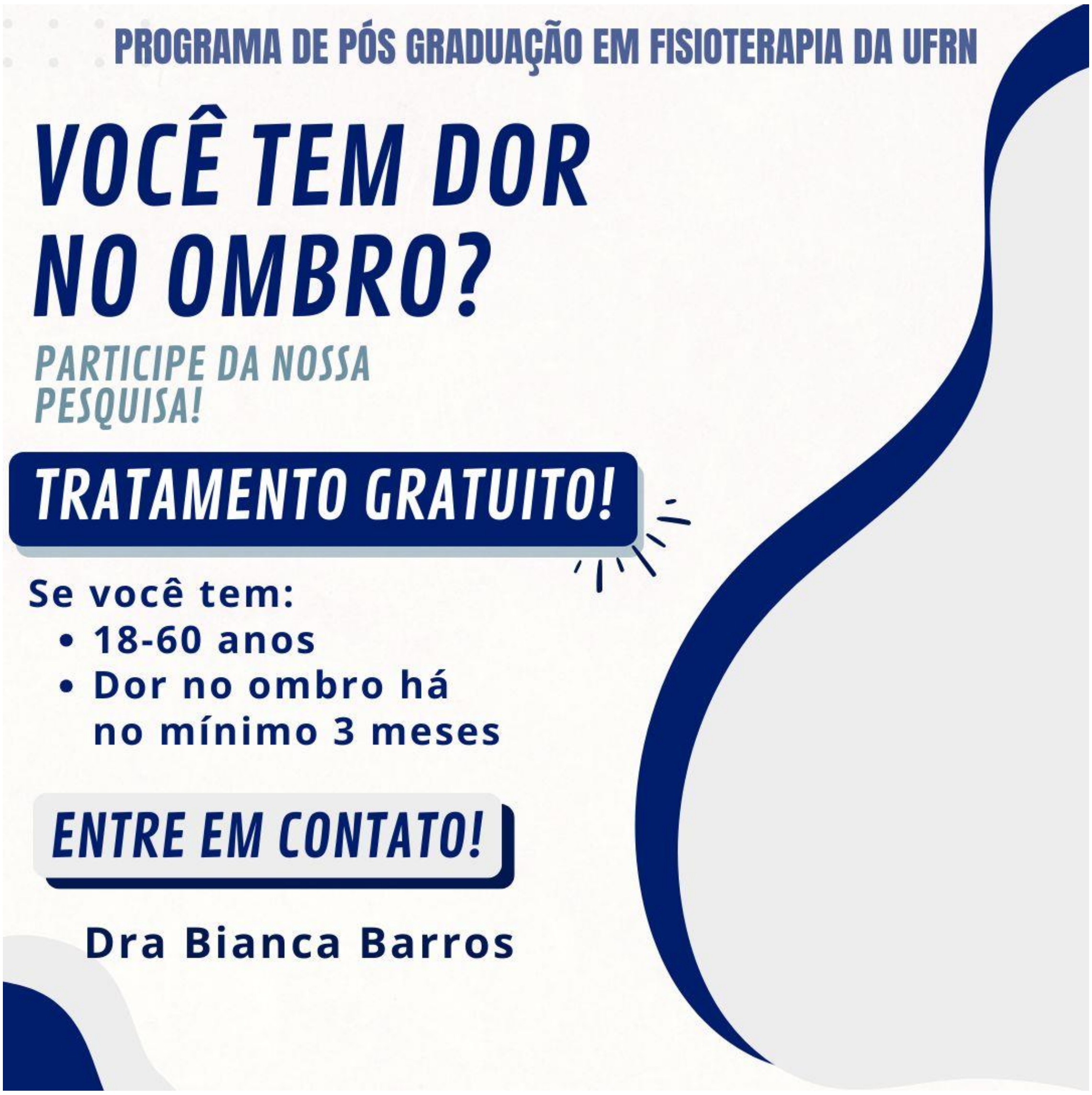

Appendice B. Informed Consent

FEDERAL UNIVERSITY OF RIO GRANDE DO NORTE
HEALTH SCIENCE CENTER
DEPARTMENT OF PHYSICAL THERAPY
INFORMED CONSENT

This is an invitation to participate in the research: isometric *versus* isotonic exercise in individuals with rotator cuff tendinopathy – effects on shoulder pain, function, muscle strength, and neuromuscular control: a randomized clinical trial, developed by the PhD student in Physiotherapy Bianca Rodrigues da Silva Barros, whose principal investigator is Professor Catarina de Oliveira Sousa.

The aim of this research is to compare and characterize the effects of two types of shoulder strengthening exercises on shoulder pain, function, and control in individuals with rotator cuff tendinopathy, a muscle group responsible for stabilizing the shoulder.

The reason that led us to carry out this study is to better understand the effects and characteristics of strengthening exercises used in the treatment of shoulder disorders. Conservative treatment, based on physiotherapeutic intervention, is indicated for the treatment of tendinopathies and ruptures of the rotator cuff muscles, especially resistance training that progressively imposes load on the tendon, in order to help it to repair by altering its metabolism, mechanical and structural properties. Among the various forms of strengthening, eccentric and concentric exercises (which are performed with shoulder movement) have been shown to be effective in improving general shoulder function, and few studies have been developed evaluating the effects of isometric exercise (performed without shoulder motion) in rotator cuff tendinopathy.

If you have rotator cuff tendinopathy diagnosed by an orthopedic surgeon through MRI or ultrasound imaging, and you decide to accept the invitation, you will undergo some assessment and intervention procedures. The assessment procedures will occur in three moments, and you will need to come in three

different days. The intervention procedures will be done twice a week for a total period of six weeks. Your participation is voluntary, and you may withdraw from the study at any time if you wish. In the first moment of the evaluation, you will be examined by a physical therapist with six years of experience. This evaluation consists of taking personal data and medical history, and physical examination to see if you have signs and symptoms of tendinopathy in one of the rotator cuff muscles, and to exclude other painful shoulder conditions.

After completing the initial assessment, you will undergo an evaluation to determine the load and familiarize yourself with the exercises which will be performed during intervention. Between 3 and 7 days you will be scheduled to the assessment of shoulder pain, function, electromyography activity of muscles, and analysis of shoulder movement. These assessments will be repeated in two other moments: right after the first training session with the exercises and after the total training period, at the end of the 6 weeks. For the evaluation of the electrical activity of the muscles and movement, sensors will be attached with double-sided adhesives and hypoallergenic tape at specific anatomical points on the shoulder and you will be instructed to perform some arm elevation movements. These sensors do not generate any stimulation or painful sensations.

After these evaluation procedures, you will participate in a 6-week training program, according to the group you will be allocated to by lot: 1) exercises involving neck and shoulder musculature stretching, strengthening of the muscles that move the scapula and strengthening of the rotator cuff muscles isometrically; or 2) exercises involving neck and shoulder muscle stretching, strengthening the muscles that move the scapula and isotonic strengthening of the rotator cuff muscles.

All procedures, from assessments to the intervention program, will be performed at the Neuromuscular Performance Analysis Laboratory at the Physical Therapy Department of the Federal University of Rio Grande do Norte (UFRN), ensuring complete privacy for study participants.

The assessments and interventions are not invasive, however, during the research, occasional muscle discomfort may occur as a result of performing the

physical exercises, which can be minimized with the application of cryotherapy to reduce these discomforts.

As benefits from the research, you will receive a complete assessment of your shoulder and receive a training program aimed at reducing pain and improving function and motor control of your shoulder. If we observe at the end of the research a superiority in the results of one group over the other, we will guarantee the participants that treatment protocol with the best results.

In case of any problem that you may have related to the research, you will have the right to free assistance that will be provided by those responsible for the research at the UFRN Physiotherapy service.

During the entire period of the research, you will be able to resolve your doubts by calling Professor Catarina de Oliveira Sousa (responsible researcher).

You have the right to refuse to participate or withdraw your consent at any stage of the research, without prejudice to you.

The data you will provide us will be confidential and will only be disclosed in congresses or scientific publications, always anonymously, with no disclosure of any data that could identify you. These data will be kept by the researcher responsible for this research in a safe place and for a period of 5 years.

Any eventual expenses incurred by your participation in this research, they will be assumed by the responsible researcher and reimbursed to you.

If you suffer any damage because of this research, whether immediate or delayed, foreseen or unforeseen, you will be indemnified.

If you have any questions about the ethics of this research, you should call the Research Ethics Committee – an institution that assesses the ethics of research before they begin and provides protection to participants – at the Federal University of Rio Grande do Norte. You can still go in person to their headquarters, from Monday to Friday, from 08:00h to 12:00h and from 14:00h to 18:00h, at the Federal University of Rio Grande do Norte, Av. Senator Salgado Filho, s/n. Central Campus, Lagoa Nova. Natal, RN.

This document has been printed in duplicate. One will stay with you and the other with the responsible researcher Professor Catarina de Oliveira Sousa.

Statement of consent

After being clarified about the objectives, importance and way in which the

data will be collected in this research, in addition to knowing the risks, discomforts and benefits that it will bring to me and having become aware of all my rights, I agree to participate in the research " Isometric versus isotonic exercise in rotator cuff tendinopathy – effects on pain, function, muscle strength and neuromuscular control: a randomized clinical trial", and I authorize the disclosure of the information provided by me in congresses and/or scientific publications as long as no data can identify me.

Natal (RN), / / .

Participant's fingerprint

Participant's signature

Statement by the principal investigator

As the principal investigator of the study "Isometric versus isotonic exercise in rotator cuff tendinopathy – effects on pain, function and neuromuscular control: a randomized clinical trial", I declare that I assume full responsibility for faithfully complying with the methodological procedures and rights that have been clarified and assured to the participant of this study, as well as maintaining secrecy and confidentiality about his/her identity.

I also declare that I am aware that in failing to comply with the commitment assumed herein, I will be violating the rules and guidelines proposed by Resolution 466/12 of the National Health Council - CNS, which regulates research involving human beings.

Natal _____/_____/_______.

Prof. Dr. Catarina de Oliveira Sousa
Principal investigator

Appendice C. Individual Assessment Form

ASSESSMENT FORM

Identification number: ___________________________ Phone number: (   ) ____________
Screening date _____/______/_____
Age ______years old   Date of birth ____/____/_____   Occupation _______________
Gender   ○ Male   ○Female
Race   ○White   ○Black   ○Brown   ○Indian   ○Yellow

Are you able to read and understand Portuguese?   ○ Yes ○ No

CLINICAL HISTORY
Shoulder pain: ○ Yes ○ No   ○ Right   ○ Left   ○ Both
Onset of Symptoms:
Have you had previous treatment?   ○ Yes ○ No   Which:
Have you been taking any medication? ○ Yes ○ No   Which:
Do you have history of trauma on the arm? ○ Yes ○ No When?   What?
Do you have history of systemic connective tissue, orthopedic, or neurological disease? ○ Yes ○ No   Which:
Have you been diagnosed with frozen shoulder? ○ Yes ○ No
Have you had surgery in the upper limbs? ○ Yes ○ No
PHYSICL EXAM
Pain at palpation of RC tendons? ○ Yes ○ No   Which:
Range of painful arc when raising the arm:<br><br>Active: ○ Yes ○ No<br><br>Resisted: ○ Yes ○ No
Special tests:<br><br>Jobe Test: ○ Yes ○ No<br><br>Resisted external rotation: ○ Yes ○ No<br><br>Apprehension and relocation test: ○ Yes ○ No
ANTHROPOMETRIC DATA / PHYSICAL ACTIVITY
Dominant upper limb? ○ Right   ○ Left   ○ Both
Weight: ______   Height:_______   BMI:______
Do you do physical activity? ○ Yes ○ No. How long? ____________<br>Type of activity? _____________________<br>Frequency: ○2x/week ○ 3 x/week ○ 5 x/week
