# Supplementary material for: Isometric versus isotonic exercise in individuals with rotator cuff tendinopathy—Effects on shoulder pain, functioning, muscle strength, and electromyographic activity: A protocol for randomized clinical trial
Source: PLoS One. 2023 Nov 13;18(11):e0293457. doi: 10.1371/journal.pone.0293457 (PMC10642785; doi:10.1371/journal.pone.0293457)
Supplement: S4 File — (PDF) [file pone.0293457.s004.pdf]

UNIVERSIDADE FEDERAL DO RIO GRANDE DO NORTE
CENTRO DE CIÊNCIAS DA SAÚDE
PROGRAMA DE PÓS GRADUAÇÃO EM FISIOTERAPIA
DEPARTAMENTO DE FISIOTERAPIA

BIANCA RODRIGUES DA SILVA BARROS

EXERCÍCIO ISOMÉTRICO *VERSUS* ISOTÔNICO NA TENDINOPATIA DO MANGUITO ROTADOR – EFEITOS NA DOR, FUNÇÃO E CONTROLE NEUROMUSCULAR: UM ENSAIO CLÍNICO RANDOMIZADO

NATAL – RN
2019

UNIVERSIDADE FEDERAL DO RIO GRANDE DO NORTE
CENTRO DE CIÊNCIAS DA SAÚDE
PROGRAMA DE PÓS GRADUAÇÃO EM FISIOTERAPIA
DEPARTAMENTO DE FISIOTERAPIA

BIANCA RODRIGUES DA SILVA BARROS

EXERCÍCIO ISOMÉTRICO *VERSUS* ISOTÔNICO NA TENDINOPATIA DO MANGUITO ROTADOR – EFEITOS NA DOR, FUNÇÃO E CONTROLE NEUROMUSCULAR: UM ENSAIO CLÍNICO RANDOMIZADO

Orientadora: Profª. Drª. Catarina de Oliveira Sousa

Projeto de pesquisa de doutorado apresentado ao Comitê de Ética e Pesquisa em Seres Humanos da Universidade Federal do Rio Grande do Norte– CEP/UFRN.

NATAL – RN

2019

RESUMO

O objetivo desse projeto é caracterizar os efeitos de dois tipos de exercícios – isométrico *versus* isotônico – na dor e função do ombro e a relação com o controle neuromuscular em indivíduos com tendinopatia do manguito rotador (MR). Quarenta e seis indivíduos (18-60 anos) com dor no ombro (>3 meses) e comprovada tendinopatia unilateral no suprespinhal e/ou infraespinhal por meio de exames de ultrassonografia ou ressonância nuclear magnética, participarão deste estudo. Os indivíduos serão alocados aleatoriamente em um dos dois grupos de exercícios: isométrico e isotônico. Eles serão avaliados antes e após a primeira sessão de tratamento, e novamente após seis semanas de tratamento. Os desfechos a serem avaliados são: a) dor e função geral do ombro, por meio do *Penn Shoulder Score* e o questionário *The Western Ontario Rotator Cuff Index*; b) controle neuromuscular por meio da atividade eletromiográfica do trapézio (porção inferior), serrátil anterior, deltóide (porção média), e infraespinal, durante as contrações isométricas máximas de elevação do ombro em 90º, rotações medial e lateral do ombro em 0º de elevação do braço e durante elevação do braço a 30, 60, 90 e 120 graus com e sem carga usando um sistema de registro de sinal eletromiográfico de superfície (*EMG system do Brasil®*); nós iremos avaliar a amplitude de atividade muscular de cada músculo e a razão entre os pares musculares: 1) infraespinhal e deltóide médio; 2) infraespinhal e trapézio inferior; e 3) trapézio inferior e serrátil anterior; e c) performance funcional por meio de testes que simulam atividades de vida diária, os quais incluem: alcançar um ponto acima da cabeça; alcançar a parte posterior da cabeça; e alcançar as costas ou a escápula oposta, onde serão avaliados o tempo de realização e os níveis de dor e esforço. Os dois grupos serão submetidos a um protocolo de alongamento e fortalecimento da musculatura periescapular. O grupo isométrico irá desenvolver adicionais exercícios para o MR, com 3 repetições de 32s a 70% da força isométrica máxima para cada exercício. O grupo isotônico irá desenvolver os exercícios para o MR de forma concêntrica e excêntrica, com 3 séries de 8 repetições para cada exercício a 8 RM. A carga de exercícios dos dois grupos irá ser ajustada nas semanas 3 e 5 do protocolo de seis semanas. Os efeitos do tratamento entre os grupos irão ser avaliados usando ANOVAs *two-way* com medidas repetidas, com o auxílio do *software Statistical Package for the Social Sciences* (SPSS). Os resultados deste estudo irão contribuir para o conhecimento no campo da avaliação e reabilitação do complexo do ombro e poderão subsidiar o processo de tomada de decisão no tratamento da tendinopatia do MR.

Palavras-chave: Manejo da dor; terapia por exercício; eletromiografia; movimento; síndrome do impacto subacromial.

Sumário

1 Introdução... 5

2 Justificativa.. 8

3 Objetivos... 9

3.1 Objetivo geral.. 9

3.2 Objetivos específicos... 9

4 Hipótese.. 9

5 Métodos.. 9

5.1 Características do estudo.. 9

5.2 Amostra.. 10

5.3 Critérios de Elegibilidade do estudo... 11

5.4 Procedimentos de avaliação... 12

5.4.1 Avaliação da dor e função... 14

5.4.2 Avaliação da atividade eletromiográfica durante a contração isométrica máxima e durante a elevação do braço.. 14

5.4.3 Avaliação da performance funcional... 16

5.5 Protocolos de Intervenção.. 17

5.5.1 *Alongamento e fortalecimento da musculatura periescapular*....................................... 18

5.5.2 *Grupo Exercício Isométrico*.. 18

5.5.3 *Grupo Exercício Isotônico*.. 19

5.6 Aspectos Éticos.. 20

5.7 Análise Estatística... 21

6 Desfecho e Resultados Esperados.. 21

7 Cronograma de Execução... 24

8 Orçamento detalhado e justificado... 25

Referências... 26

Anexos.. 32

Apêndices.. 40

1 Introdução

A dor no ombro é um problema comum, sendo a terceira causa mais frequente de queixa musculoesquelética (1). As disfunções que acometem o ombro podem ser caracterizadas por limitações da amplitude de movimento devido à dor e restrições nas atividades da vida diária, diminuindo a independência funcional e afetando a qualidade de vida dos indivíduos acometidos (2). Dentre as causas mais comuns de dor no ombro está a disfunção manguito rotador (MR), que consiste de tendinopatia de um ou mais dos quatro músculos do MR, que pode evoluir para ruptura parcial ou total dos seus tendões, associada ou não à inflamação das bursas (3,4).

A função e o movimento efetivo do ombro são alcançados por meio de um esforço combinado dos músculos estabilizadores da escápula, deltóide, músculos do MR, bem como grande dorsal e peitoral maior (4). Entretanto, acredita-se que a atividade coordenada dos músculos do MR (supraespinal, infraespinal, redondo menor e subescapular) seja a responsável pela estabilidade mecânica da articulação glenoumeral por meio da estabilização ou compressão da cabeça umeral dentro da fossa glenóide durante os movimentos do ombro (3,4).

Múltiplos fatores podem contribuir para o desenvolvimento da tendinopatia do MR, sendo esses classificados como: extrínsecos, que se originam externamente aos tendões causando compressão ou cisalhamento aos mesmos, devido alterações anatômicas e biomecânicas; e os intrínsecos, que se originam devido alterações nas propriedades mecânicas, morfológicas e vasculares dos tendões, bem como predisposição genética (5,4). Ambos os fatores podem estar relacionados e serem potencializados com a idade e a excessiva carga mecânica (5,6,3,4).

A tendinopatia do MR, relacionada à síndrome do impacto subacromial, tem sido associada a alterações no controle motor, uma vez que está bem descrito na literatura que existem alterações na cinemática escapular durante a elevação do braço, como a redução da inclinação posterior e rotação superior e aumento na rotação interna da escápula em indivíduos com impacto subacromial (7,8). Em associação aos movimentos, é verificada alterada ativação da musculatura periescapular durante a elevação do braço, como diminuição da ativação do

serrátil anterior e porções média e inferior do trapézio (9,10), e aumento da ativação da porção superior do trapézio (11,12,2,9). Além disso, é verificada falha na coordenação entre trapézio inferior e serrátil anterior e entre as porções superior e inferior do trapézio, evidenciada pela alteração razão de ativação entre esses músculos (13).

Considerando a ativação dos músculos do MR, verifica-se reduzida co-ativação do MR e aumentada ativação do deltóide médio no início da elevação umeral (14) e do deltóide posterior, quando o MR está fadigado, lesionado ou rompido, como uma compensação para evitar a redução do movimento de elevação do braço e estabilizar a glenoumeral (15,16). Esse desequilíbrio entre a ativação do MR e o deltóide pode contribuir para excessiva translação superior da cabeça umeral (17) e, assim, maior risco para desenvolvimento da síndrome do impacto subacromial e disfunções do MR.

Embora sejam verificadas essas alterações musculares e no movimento, há pouca evidência para apoiar se essas alterações são causadas pela dor proveniente da condição clínica ou se esta é proveniente do efeito cumulativo das alterações de ativação muscular e de movimento. Ademais, a causa da dor local na tendinopatia permanece elusiva, visto que o nível de dor varia substancialmente e nem sempre está relacionada à patologia local, sugerindo uma sensibilização central em indivíduos com tendinopatia do MR (3). Assim, além do foco em intervenções que reduzam o risco de impacto subacromial, é importante focar em intervenções que visem à melhora na dor, a fim de investigar a relação entre a condição clínica, dor e a coordenação neuromuscular nesses indivíduos.

A intervenção primária para tratar a tendinopatia do MR é o exercício terapêutico ativo (3), a qual tem proporcionado resultados similares aos das intervenções cirúrgicas, mas com benefícios adicionais do exercício, com menos absenteísmo, retorno rápido ao trabalho e reduzido custo aos sistemas de saúde (18). Muitas estratégias de exercícios têm sido propostas, entretanto, persiste a incerteza a respeito da mais eficaz para tratar a dor, fraqueza e perda de função associada à tendinopatia do MR (3). No entanto, é prioridade a redução da dor (3) e os exercícios com carga mecânica, os quais estimulam a resposta cicatricial do tendão, acelera o metabolismo dos tenócitos e a reparação tecidual (19,20).

Nesse sentido, o treinamento de resistência em geral, incluindo isometria e exercícios isotônicos, com foco tanto no MR quanto nos músculos estabilizadores da escápula, em pacientes com tendinopatia do MR e síndrome do impacto subacromial tem se mostrado eficazes para a melhora da dor e função (21). Comparação de intervenções de exercícios excêntricos ou concêntricos isolados não verificou diferença na eficácia, com ambos resultando em melhora na função, amplitude de movimento e força (22). A respeito das contrações isométricas, existe evidência de que estas auxiliam no controle da dor quando utilizado nas tendinopatias do membro inferior, mas ainda não se tem evidências conclusivas sobre seus efeitos nas tendinopatias do MR (23).

Exercícios isotônicos de alta carga têm se mostrado eficazes para a reabilitação de tendinopatias de membro inferior, como a tendinopatia patelar (24) e a tendinopatia de Aquiles (25). No entanto, nos últimos anos, os exercícios isométricos têm surgido como uma excelente ferramenta para reabilitação de tendinopatias (26–30). Exercícios isométricos têm se mostrado mais eficazes para a dor e função do quadríceps, tanto com durações de contração curta quanto longa (26), e mais efetivo para redução da dor, aumento da força e redução da inibição cortical quando comparado com o exercício isotônico, de maneira imediata (27) e após 4 semanas (28) quando utilizado no tratamento de tendinopatia patelar. Além disso, o exercício isométrico submáximo tem mostrado aumentar o limiar de dor a pressão, reduzir a percepção dolorosa ao calor e reduzir a somação temporal da dor em homens e mulheres (29). Aplicado a tendinopatia do MR, temos até o momento dados de um estudo piloto (30), o qual sugere que exercícios isométricos de baixa carga para tendinopatia do MR podem influenciar positivamente a dor e a rigidez do tendão.

Apesar desses estudos indicarem efeitos positivos dos exercícios isométricos na dor, função e redução da inibição cortical em indivíduos com tendinopatia, pelo nosso conhecimento, nenhum estudo avaliou os efeitos de um programa de fortalecimento isométrico do MR na dor, função e no controle neuromuscular entre os músculos do MR e músculos periescapulares e deltóide durante a tarefa de elevação do braço em comparação a um programa de fortalecimento isotônico do MR. Assim, o objetivo deste estudo é verificar os efeitos do exercício isométrico, aplicado aos músculos do MR, comparado ao

exercício isotônico, na dor, função e controle neuromuscular em indivíduos com tendinopatia do MR.

2 Justificativa

Disfunções nos tendões do MR, tanto sintomáticas quanto assintomáticas, têm uma alta prevalência na população em geral, podendo chegar a 30% (31). Essas disfunções estão relacionadas a fatores biomecânicos, hábitos de vida e predisposição genética, e influenciadas pela idade e sobrecarga imposta aos tendões (5,32). A grande relevância clínica dessa condição se deve ao fato de que há um alto risco de progressão da tendinopatia para ruptura parcial ou total dos tendões, ocasionando em sintomas de dor, fraqueza (32), e importantes limitações funcionais (33).

Tratamento conservador, baseado em intervenção fisioterapêutica, é indicado para o tratamento das tendinopatias e rupturas do MR (32), especialmente o treinamento de resistido que impõe carga ao tendão de forma progressiva, a fim de auxiliar na sua reparação por meio da alteração de seu metabolismo e propriedades mecânicas e estruturais (6). Dentre as diversas formas de resistência, os exercícios excêntrico e concêntrico têm se mostrado eficazes para a melhora da função geral do ombro (22), e poucos estudos (4,30) têm sido desenvolvidos avaliando os efeitos do exercício isométrico na tendinopatia do MR.

Entretanto, o exercício isométrico tem surgido como uma importante abordagem para melhora da dor em tendinopatias (27–29) e, quando comparado ao exercício isotônico, tem se mostrado superior nos resultados de dor, força, função em geral, bem como na redução da inibição cortical no tratamento de tendinopatia patelar (27). Assim, justifica-se a relevância clínica de se avaliar os efeitos do exercício isométrico aplicado à tendinopatia do MR na dor, função e controle neuromuscular envolvido no complexo do ombro. Nesse contexto, este estudo pode auxiliar na tomada de decisão clínica quanto a melhor abordagem a ser considerada no tratamento da tendinopatia do MR.

3 Objetivos

3.1 Objetivo Geral:

Caracterizar os efeitos de dois tipos de exercícios – isométrico *versus* isotônico – na dor e função do ombro e a relação com o controle neuromuscular em indivíduos com tendinopatia do MR.

3.2 Objetivos Específicos:

- Avaliar as repercussões dos exercícios isométrico e isotônico na dor e função de indivíduos com tendinopatia do MR;
- Verificar os efeitos dos exercícios isométrico e isotônico no controle neuromuscular dos músculos do complexo do ombro por meio da atividade eletromiográfica durante as contrações isométricas máximas e na tarefa motora de elevação do braço de indivíduos com tendinopatia do MR;
- Analisar a relação entre os efeitos na dor, controle neuromuscular, performance funcional e função do complexo do ombro;
- Verificar os efeitos do exercício isométrico comparado ao isotônico nas variáveis descritas, imediatamente após uma sessão de exercícios e após seis semanas de intervenção em indivíduos com tendinopatia do MR.

4 Hipóteses

Conforme os resultados positivos e superiores apresentados a partir do exercício isométrico aplicado à tendinopatia patelar em relação ao exercício isotônico, tanto imediatamente quanto após um período de intervenção, na dor, força e função do quadríceps, hipotetizamos que esta modalidade de exercício será superior ao exercício isotônico em indivíduos com tendinopatia do MR, tanto de maneira imediata quanto após 6 semanas de intervenção. Hipotetizamos que o exercício isométrico pode reduzir a dor e melhorar a performance funcional e o controle neuromuscular do complexo do ombro, resultando assim em melhor função nessa população.

5 Métodos

5.1 Características do estudo

Este estudo é caracterizado como do tipo ensaio clínico randomizado, que será constituído por dois grupos, e desenvolvido no Departamento de Fisioterapia da Universidade Federal do Rio Grande do Norte.

5.2 Amostra

Participarão deste estudo 46 indivíduos com diagnóstico clínico de tendinopatia unilateral do tendão do supraespinal e/ou infraespinal por meio de exames de ultrassonografia (US) ou ressonância nuclear magnética (RNM), entre 18 e 60 anos de idade, de ambos os sexos, os quais serão divididos e alocados aleatoriamente em dois grupos de exercícios, com 23 indivíduos cada: 1) grupo isométrico, que será submetido a um protocolo de treino resistido isométrico para a musculatura do MR associado a um protocolo de alongamento e fortalecimento voltado para musculatura periescapular; e 2) grupo isotônico, que será submetido a um protocolo de treino resistido isotônico para a musculatura do MR associado a um protocolo de alongamento e fortalecimento voltado para musculatura periescapular.

A amostragem será do tipo não-probabilística de casos consecutivos, onde os indivíduos serão recrutados a partir de uma lista de espera por atendimento fisioterapêutico do setor de Fisioterapia da UFRN e por meio de divulgação realizada na UFRN, na cidade de Natal-RN e redes sociais por meio de cartazes. O tamanho da amostra foi determinado considerando a variável de desfecho principal, a dor durante a elevação do braço sem carga, considerando uma diferença entre médias de 2,05 e desvio padrão de 2,30 na ENAD (34). Adotando um Power de 80% e $\alpha = 0{,}05$, serão necessários pelo menos 21 indivíduos por grupo. Levando em consideração possíveis perdas amostrais de aproximadamente 10%, a amostra deverá ser de pelo menos 23 indivíduos por grupo.

Este projeto será enviado ao Comitê de Ética e Pesquisa da UFRN (CEP/UFRN), por meio do cadastro na Plataforma Brasil, e apenas iniciará suas atividades após aprovação. Todos os voluntários receberão uma explicação verbal e escrita quanto aos objetivos e a metodologia do estudo, bem como seus riscos e benefícios; e os que aceitarem participar deverão assinar um termo de consentimento livre e esclarecido, de acordo com a Resolução 466/12 do Conselho Nacional de Saúde, confirmando a participação no estudo. O projeto

será submetido ao Registro Brasileiro de Ensaios Clínicos (http://www.ensaiosclinicos.gov.br/) antes do início do recrutamento dos indivíduos.

5.3 Critérios de elegibilidade do estudo

Serão incluídos no estudo indivíduos de ambos os sexos, de 18 a 60 anos, com história de dor no ombro há mais de 3 meses, e com diagnóstico clínico de tendinopatia do manguito rotador por meio de exames de US ou RNM, realizados por um médico ortopedista (4).

Em seguida, uma fisioterapeuta com 6 anos de experiência realizará uma avaliação dos indivíduos recrutados com coleta de dados pessoais e história clínica, e exame físico, que consistirá de palpação dos tendões do manguito rotador, avaliação da amplitude do arco doloroso durante a elevação do braço nos três planos de movimento do ombro ativa e resistida, e a realização dos seguintes testes especiais para detectar disfunção nos tendões do supraespinal e infraespinal: teste de Jobe para avaliar lesão no tendão do supraespinhal (35), rotação externa resistida para lesão no infraespinhal (36), testes de apreensão e recolocação para lesão na porção póstero-superior do infraespinhal, característica de impacto interno (35).

O diagnóstico de tendinopatia do MR será realizado por um médico cirurgião ortopedista especialista na área do complexo do ombro, de acordo com a história clínica, exame físico e alterações morfológicas dos tendões do MR, visualizadas por meio da RNM ou US: hipoecogenicidade do tendão e/ou espessamento das fibras do tendão com ou sem espessamento bursal (37).

Serão excluídos os indivíduos que pratiquem atividade esportiva com alta demanda dos membros superiores; que apresentem comprometimentos na cabeça longa do bíceps; capsulite adesiva (38); história de início de sintoma devido à luxação ou subluxação da glenoumeral, ou à fratura na clavícula, escápula ou úmero (39); história de estabilização cirúrgica ou reparo do manguito rotador (40); sinais de ruptura parcial ou completa do manguito rotador (12,40); disfunções neurológicas (41); uso de injeção de corticóide 3 meses antes da avaliação (39) e índice de massa corporal (IMC)>28kg/m^2, uma vez que isso pode

comprometer a qualidade dos dados da eletromiografia (39), indivíduos que estiverem em uso de antibióticos da classe das fluoroquinolonas (42) e/ou com diabetes (43), pois ambos afetam o metabolismo dos tendões.

5.4 Procedimentos de avaliação

Todos os indivíduos incluídos serão submetidos às seguintes avaliações: a) dor e função geral do complexo do ombro; b) atividade eletromiográfica da musculatura do complexo do ombro durante contrações isométricas máximas; e c) atividade eletromiográfica dos músculos do complexo do ombro durante a elevação do braço no plano da escápula.

Esta sequência de avaliação será realizada em três momentos: 1) avaliação inicial, imediatamente antes da primeira sessão de intervenção; 2) imediatamente após a primeira sessão de exercícios; e 3) após seis semanas de intervenção. Todas as avaliações serão realizadas por um avaliador que não saberá a que grupo de intervenção os indivíduos pertencem (avaliador cego), sendo as intervenções aplicadas por dois outros pesquisadores treinados.

Uma semana antes da avaliação inicial, os indivíduos serão alocados de maneira randomizada, por meio da ferramenta online de aleatorização para ensaios clínicos randomizados, disponíveis no site (www.randomization.com). Para garantir o sigilo de alocação dos indivíduos, esta será realizada por um dos responsáveis pela aplicação da intervenção, o qual será o único a ter conhecimento a que grupo os indivíduos pertencem. Após a determinação do grupo, os indivíduos serão apresentados ao processo e aos equipamentos de avaliação, e farão um teste para determinar a faixa elástica a ser utilizada durante os exercícios de fortalecimento da musculatura periescapular. Além disso, os indivíduos que forem alocados no grupo de exercícios isométricos ou isotônicos farão o teste de determinação inicial da carga para cada um dos exercícios. As sequências dos procedimentos do estudo estão descritas na Figura 1.

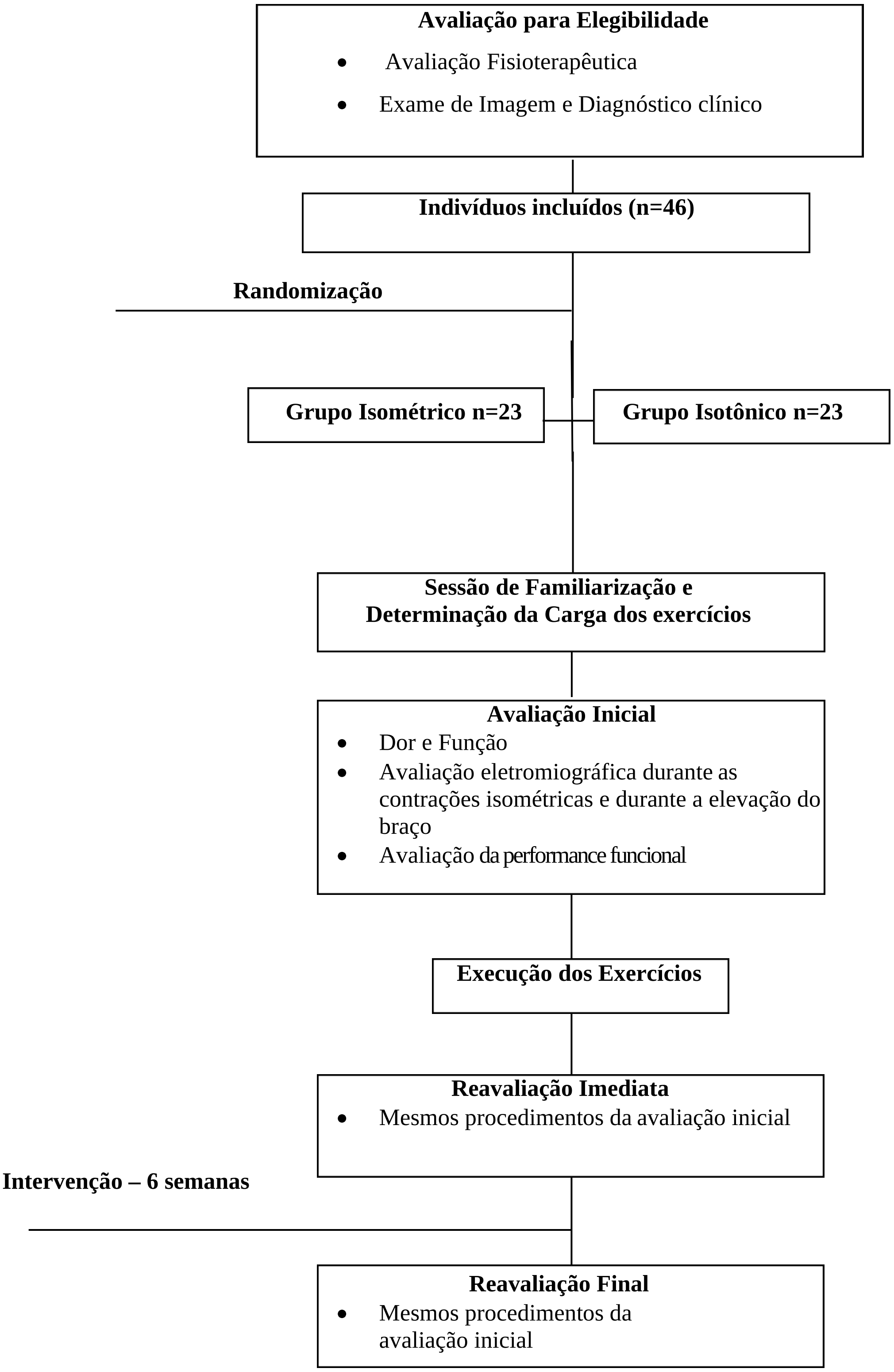

Figura 1. Fluxograma do desenho do estudo.

5.4.1 Avaliação da dor e função

Para avaliar a dor e função do ombro, será utilizada a versão brasileira do questionário *Penn Shoulder Score* (PSS), que consiste de uma escala que inclui os domínios de dor, satisfação e função. O domínio de dor e satisfação apresentam, respectivamente, três itens (dor em repouso, dor durante atividades normais e dor durante atividades estenuantes) e um item (satisfação com nível atual de função do ombro) avaliados por meio de uma escala de avaliação numérica (EN) de 0 a 10, sendo que 0 corresponde à ausência de dor e a não satisfeito, enquanto 10 corresponde à pior dor possível e a muito satisfeito. O domínio de função contém vinte itens, graduados em uma escala de Likert de quatro pontos, variando de 0, que significa "não consigo fazer de forma alguma", a 3, "sem dificuldade", com pontuação máxima de 60 pontos. A pontuação do PSS varia de 0 a 100 pontos, onde o escore máximo (100) indica nenhuma dor, alta satisfação e boa função (44).

Além disso, serão avaliados os aspectos da qualidade de vida relacionados por meio da versão brasileira do questionário *Western Ontario Rotator Cuff Index* (WORC), elaborado e validado para indivíduos com disfunção do MR, e consiste de 21 itens em cinco domínios de vida e saúde (sintomas físicos, esporte/recreação, trabalho, estilo de vida, e emoções). Cada um dos itens possui o mesmo peso e varia de 0 a 100 (100 mm numa escala visual analógica – EVA), e o total do questionário varia de 0 a 2100 mm, que são convertidos a um escore de 100%, onde 0% significa o pior escore possível e 100% implica nenhuma redução na qualidade de vida relacionada à saúde (45).

5.4.2 Avaliação da atividade eletromiográfica durante a contração isométrica máxima e elevação do braço com e sem carga

As contrações isométricas máximas serão realizadas com um dinamômetro (Nextech, DFS-X1000, Nextech Global Company Limited, Thailand), que será fixado em um aparato que garante a isometria da contração, e os indivíduos serão instruídos a realizar uma força no sentido da elevação do braço e das rotações lateral e medial contra o equipamento com a máxima força possível. Para a contração na elevação do braço, os indivíduos estarão sentados em uma cadeira com apoio e ombro a 90º de elevação no plano escapular, e para a rotação lateral e medial do ombro, estarão sentados com o ombro posicionado

a 0º de abdução, cotovelo flexionado em 90º e punho em posição neutra (34). Os indivíduos serão encorajados verbalmente, mantendo a contração por cinco segundos. Cada teste será realizado duas vezes, com intervalo de dois minutos entre as tentativas, e a média dos valores será utilizada nas análises.

A elevação do braço será realizada com o indivíduo em bipedestação e será realizada a elevação no plano escapular (40º de abdução úmero-torácica) usando uma superfície vertical planar como referência, nesta mesma superfície serão marcados 30, 60, 90 e 120 graus de elevação de cada indivíduo. Na sequência, os indivíduos realizarão elevação em cada um desses ângulos com carga e depois com carga, sustentando um peso de 1,5 kg para aqueles que apresentarem menos de 68 kg de peso corporal e um peso de 2,5 kg para aqueles que apresentarem mais de 68,1 kg de peso corporal com e sem carga (13).

Durante a realização das contrações isométricas máximas e da elevação do braço nos diferentes ângulos com e sem carga, será coletado o sinal eletromiográfico dos músculos trapézio (porção inferior), serrátil anterior, deltóide (porção média), e infraespinhal.

Para aquisição e processamento dos sinais eletromiográficos, será utilizado um módulo condicionador de sinais (MSC 1000) de 8 canais (EMG system do Brasil®) com um conversor analógico digital – A/D (CAD, 12/36-60 K) com resolução de 12 bits. O equipamento tem uma razão de rejeição de modo comum (RRMC) maior de 80 Db, com frequência de amostragem de 2.000 Hz e o sinal filtrado entre 20 e 500 Hz (Figura 02). Como o ganho programado no conversor é de 50 vezes e de 20 vezes nos eletrodos, os sinais serão amplificados em 1000 vezes. O eletromiógrafo será alimentado por uma bateria e conectado a um notebook que receberá o sinal e o armazenará em arquivo. O *software* EMGlab (EMG System do Brasil®, Brasil) será utilizado para análise digital dos sinais.

Serão utilizados eletrodos de superfície ativos, simples diferenciais, compostos por um sistema Ag/AgCl associado a um gel condutor, de configuração bipolar com dimensão de 4 cm X 2,2 cm de área adesivada e 1 cm de área condutora separados por uma distância inter-eletrodo de 2 cm (Miotec®, Brasil). Também será utilizado um eletrodo de referência de configuração monopolar, composto por um sistema Ag/AgCl associado a um gel condutor, com diâmetro de 3,8 cm de área adesivada e 1 cm de área condutora (Miotec®, USA).

O posicionamento dos sensores para a avaliação da porção inferior do trapézio, serrátil anterior, deltóide médio e infraespinhal seguirá o procedimento descrito em Michener et al. (46). Um eletrodo de referência será afixado no processo estilóide da ulna do lado contralateral ao lado avaliado (48). Antes da colocação dos eletrodos, a pele das regiões delimitadas será tricotomizada e limpa com álcool. A atividade eletromiográfica será avaliada apenas nos músculos do lado sintomático, e após os testes, os indivíduos serão questionados quanto a sua dor, por meio da Escala Numérica de Avaliação da Dor (0-10, sendo 0 nenhuma dor e 10 pior dor possível) (49).

Para a avaliação da amplitude do sinal eletromiográfico de cada músculo e posteriormente realizar a medida da razão de ativação muscular entre os pares musculares, os dados brutos serão filtrados a uma frequência passa-banda de 20-450 Hz, retificados e suavizados usando um algoritmo de raiz quadrada da média (RMS) com uma janela móvel de 50-milisegundos, utilizando-se o *software Matlab.*

Será avaliada a razão de ativação muscular entre os seguintes pares: 1) infraespinhal e deltóide médio; 2) infraespinhal e trapézio inferior; e 3) trapézio inferior e serrátil anterior. Esses pares musculares foram selecionados uma vez que indivíduos com disfunção de MR apresentam aumentada atividade compensatória da porção média do deltóide (14–16) e incoordenação entre trapézio inferior e serrátil anterior e entre as porções superior e inferior do trapézio (13).

5.4.3 Avaliação da performance funcional

Serão realizados três testes funcionais que simulam atividades de vida diária. Durante os três testes os indivíduos estarão em bipedestação e com os braços ao lado do corpo e irão iniciar cada um dos testes com o braço ao lado do corpo e a mão na parte lateral da coxa.

1. Alcançar um ponto acima da cabeça: os indivíduos serão posicionados com a face direcionada a uma parede, a 30 cm de distância, a qual possuirá um alvo com altura estipulada de acordo com a estatura do indivíduo, a qual será a medida da estatura do indivíduo acrescida 10%. Durante o teste, os indivíduos repetidamente alcançarão o ponto

alvo na parede, realizando a elevação do braço no plano sagital, e retornarão a posição inicial.

2. Alcanlçar a parte posterior da cabeça: os indivíduos alcançarão a parte posterior da cabeça (região occipital) e retornarão a posição inicial. Os indivíduos não poderão inclinar o tronco ou a cabeça durante o teste e o cotovelo deverá ficar alinhando lateralmente com a cabeça enquanto os indivíduos tocarão a região occipital.
3. Alcançar as costas ou a escápula oposta: os indivíduos alcançarão a sua escápula oposta, ou tentarão alcançar um ponto mais longe nas suas as costas, e retornarão a posição inicial. Os indivíduos não poderão inclinar ou fazer rotação do tronco ou a cabeça durante o teste.

Cada um dos testes será realizado 20 vezes e o mais rápido possível, uma vez de cada lado. O tempo para completar a tarefa será medido com um cronômetro.

5.5 Protocolo de intervenção

Os indivíduos de cada grupo irão realizar uma sessão de exercícios de acordo com o seu grupo (isométrico e isotônico) no dia da avaliação inicial a fim de verificar os efeitos imediatos dos exercícios, e, a seguir, realizarão uma intervenção de seis semanas, com uma frequencia de duas vezes na semana, a fim de verificar os efeitos de um programa de fortalecimento direcionado para o MR. Os exercícios serão realizados em ambos os membros.

Indivíduos de todos os grupos serão submetidos a um protocolo de alongamento e fortalecimento da musculatura periescapular seguindo o protocolo utilizado no estudo de Camargo et al (53), o qual é composto por alongamento dos músculos trapézio superior e peitoral menor e da porção posterior do ombro, e fortalecimento dos músculos serrátil anterior e trapézio inferior.

A avaliação para determinar as cargas iniciais dos exercícios será realizada uma semana antes da avaliação inicial e início das intervenções. Nesse momento, os participantes também realizarão a familiarização de cada exercício com uma resistência leve e serão orientados pelo avaliador a realizarem a técnica corretamente. A fim de ajustar e progredir a carga dos exercícios ao longo das seis

semanas de intervenção, as avaliações serão repetidas na terceira e quinta semanas de intervenção, conforme apresentado na Figura 2 com o fluxograma dos procedimentos de intervenção e progressão de cada grupo.

5.5.1 Alongamento e fortalecimento da musculatura periescapular

O dois grupos realizarão o protocolo de alongamento e fortalecimento utilizado no estudo de Camargo et al (53). O alongamento do trapézio superior será realizado de forma ativa com o indivíduo realizando a flexão lateral do pescoço, para ambos os lados; o alongamento do peitoral menor será realizado com o indivíduo de frente para duas paredes com 90º de angulação entre elas, com elevação de ombro no plano escapular de 90º e flexão de cotovelos a 90º, e o indivíduo será instruído a projetar o corpo para frente o máximo que conseguir. O alongamento da porção posterior do ombro será realizado com a adução horizontal do ombro, com os indivíduos apoiados na parede a fim de evitar estabilizar a escápula (54). Serão realizadas 3 séries de 30 segundos para cada alongamento, com um intervalo de 30 segundos entre as repetições (53).

Para fortalecimento do serrátil anterior será realizado o exercício de protração da escápula em decúbito dorsal com o ombro flexionado a 90º; e para o fortalecimento do músculo trapézio inferior serão realizados exercícios de extensão do ombro com cotovelo estendido e o indivíduo em decúbito ventral. Para as séries de fortalecimento, serão realizadas 3 séries de 10 repetições com 1 minuto de descanso entre as séries. A resistência dos exercícios será oferecida por meio de faixas elásticas com progressão determinada pela mudança nas cores das faixas, de acordo com a percepção do indivíduo, de maneira que assim que o exercício for realizado sem dificuldade e fadiga, a faixa elástica será trocada por uma de maior resistência, de forma individual (53).

5.5.2 Grupo exercício isométrico

Os exercícios isométricos voltados para os músculos do MR consistirão de 3 repetições de 32 segundos numa intensidade de 70% da força isométrica máxima para cada exercício, conforme protocolo adaptado de Rio et al. (27,28), e com intervalo entre as repetições de 80 segundos (26). A avaliação da força máxima bem como a realização dos exercícios serão realizados com um

dinamômetro manual acoplado em um anteparo rígido e preso a uma coluna fixa. O dinamômetro tem uma função que permite acompanhamento em tempo real da força desenvolvida, permitindo um *feedback* visual que indicará aos indivíduos a manutenção da carga determinada durante a execução do exercício.

A determinação da força isométrica máxima para os músculos supraespinhal e rotadores mediais e laterais será realizada durante a avaliação da força do ombro, descrita anteriormente. O posicionamento dos indivíduos para a execução dos exercícios isométricos para esses grupos musculares será a mesma da avaliação. A elevação do ombro no plano da escápula a 90º foi adotada por apresentar menor risco de compressão do supraespinhal no arco coracoacromial e, consequentemente, gerar menos dor e lesões adicionais do que exercícios em menores ângulos de elevação do braço (55). O movimento de elevação deverá ser realizado com o polegar voltado para cima, por apresentar maior seletividade do músculo supraespinhal comparado ao deltóide médio e posterior (56).

Para a realização dos exercícios, os indivíduos serão instruídos a empurrar o dinamômetro na direção do movimento desejado e seguir o *feedback* sonoro do equipamento, pré ajustado para 70% da força máxima, e o pesquisador responsável pelo treinamento dará *feedback* verbal para que os indivíduos evitem alteração de posicionamento ou compensação e para evitar excessiva contração do trapézio superior durante o exercício para supraespinhal.

5.5.3 Grupo exercício isotônico

Os exercícios isotônicos para o fortalecimento dos músculos do MR de forma concêntrica e excêntrica serão realizados utilizando halteres. Os exercícios consistirão de 3 séries de 8 repetições de cada exercício numa intensidade de 8 RM, com reavaliação da carga na terceira e quinta semana de intervenção, conforme protocolo adaptado de Kongsgaard *et al*. (24). O intervalo de descanso entre as séries de exercícios será de 80 segundos (28).

O exercício de rotação externa será realizado em decúbito lateral contralateral com o ombro a 0º de abdução e flexão e flexão de cotovelo a 90º com uma toalha entre a parte inferior do braço e o tronco dos indivíduos. Para a rotação medial, o paciente se posicionará em decúbito lateral sobre o membro a ser fortalecido com o ombro a 0º de abdução e flexão e flexão de cotovelo a 90º (22). Os participantes serão orientados a realizarem os exercícios concêntricos e

excêntricos com um tempo de 3 segundos para cada modo de contração, que será controlado por meio de um metrônomo que também servirá como *feedback* aos participantes (57).

Para o fortalecimento do supraespinhal, será realizado o movimento de elevação no plano da escápula com punho neutro no sentido do polegar para cima e na amplitude de 60 a 90º, visto que nessa amplitude há menor risco de compressão do supraespinhal no arco coracoacromial e consequentemente menos risco de aumentar a dor e gerar lesões adicionais do que em menores ângulos de elevação do braço (55).

5.6 Aspectos Éticos

O estudo será submetido à avaliação do Comitê de Ética em Pesquisa de Seres Humanos e respeitará as diretrizes e normas constadas na Resolução 466/12 do Conselho Nacional de Saúde que diz respeito à ética na realização de pesquisas com seres humanos, preconizando discrição e respeito aos participantes da pesquisa ao longo de toda a coleta dos dados, devendo garantir os deveres e direitos dos mesmos. Todos os usuários deverão assentir sua participação de espontânea vontade que será explicitada pela assinatura de duas vias do Termo de Consentimento Livre e Esclarecido (TCLE) e do Termo de Autorização para o Uso da Imagem.

Os riscos inerentes à essa pesquisa são mínimos e poderá acontecer a presença de algum desconforto doloroso durante a realização dos procedimentos de avaliação e intervenção. Dessa forma, o indivíduo possuirá o direito de desistência da pesquisa em qualquer momento que desejar e o pesquisador responsável terá o dever de sanar todas as suas dúvidas e garantir a privacidade e sigilo do participante da pesquisa, devendo sempre ser transparente acerca dos procedimentos que irão ser realizados. Caso venha a apresentar algum dano físico, será disponibilizado ao voluntário, assistência e Fisioterapêutica nas Clínicas escola das respectivas áreas.

Quanto aos benefícios promovidos, percebe que o participante estará contribuindo para a comunidade científica, a medida de incentivará uma prática fisioterapêutica baseada em evidências. Ademais, receberá uma avaliação completa e detalhada sobre as condições cinético-funcionais de seu ombro.

5.7 Análise Estatística

Os dados serão analisados estatisticamente de forma descritiva e inferencial. Média e desvio padrão serão calculados para todos os dados demográficos e variáveis dependentes. No tocante às análises inferenciais, inicialmente, será empregado o Teste de Normalidade de Shapiro-Wilk, para classificar a distribuição de cada variável.

A fim de realizar a comparação entre os dois grupos (isométrico, isotônico e controle) e entre as avaliações, para cada variável dependente, aplicaremos ANOVA two-way com medidas repetidas quando os dados forem considerados paramétricos e Friedman quando os dados forem não paramétricos.

Quando necessários, testes *post hoc* de Tukey ou Ducan serão empregados, respectivamente. Todos os testes serão realizados com auxílio do *software Statistical Package for the Social Sciences* – SPSS, versão 20.0, e o nível de significância adotado será de $\alpha \leq 0{,}05$.

6 Desfecho e Resultados Esperados

A tendinopatia do manguito rotador é uma lesão muito frequente na população em geral e o treinamento por meio de exercícios resistidos progressivos tem sido uma das principais alternativas para o tratamento conservador. Entretanto ainda não há evidências na literatura dos efeitos e benefícios de diferentes tipos de exercícios resistidos, tais como isométricos, concêntricos e excêntricos.

Desta forma, por meio dos resultados obtidos, este projeto irá contribuir para o avanço do conhecimento científico no campo da avaliação e reabilitação do complexo do ombro, especificamente na disfunção do MR. O conhecimento acerca dos efeitos do exercício isométrico aplicado à tendinopatia do MR na dor, função e controle neuromuscular envolvido no complexo do ombro é muito relevante clinicamente, uma vez que poderá subsidiar o processo de tomada de decisão fisioterapêutica no tratamento da tendinopatia do MR. As atividades que serão realizadas no decorrer desse projeto possibilitam uma importante avanço no desenvolvimento de conhecimentos especializados na abordagem do paciente com disfunções do complexo do ombro. É importante ressaltar que o desenvolvimento de projetos voltados para a avaliação e intervenção nas disfunções dos membros superiores tem grande relevância, uma vez que apesar da grande incidência de disfunções no ombro na população de modo geral, são poucos os pesquisadores que atuam nesta linha de pesquisa no Brasil. Deste modo, este projeto poderá contribuir auxiliando na capacitação de recursos humanos para produção

do conhecimento científico nessa área tão importante para a Fisioterapia.

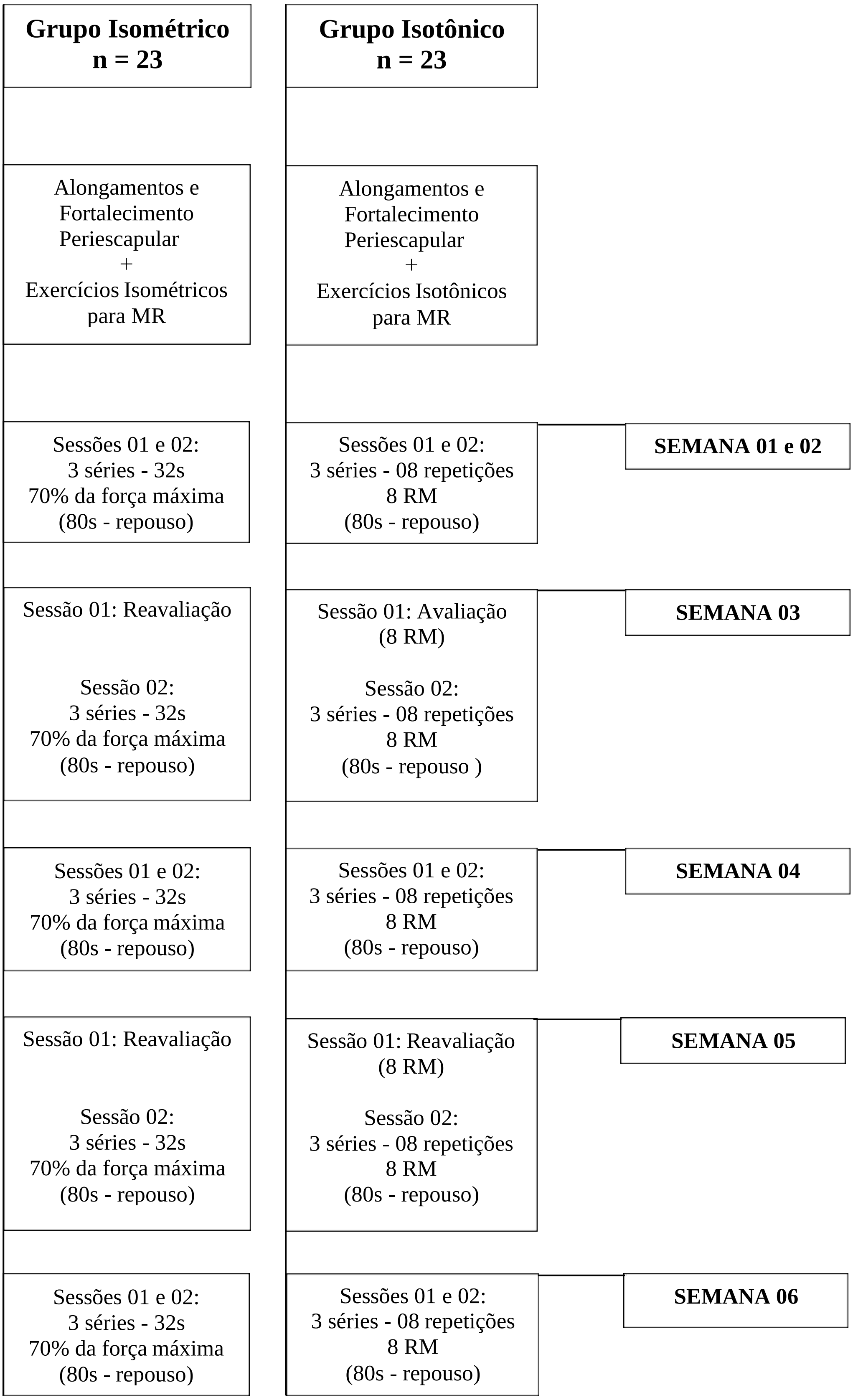

Figura 2. Fluxograma dos protocolos de intervenção e progressão .

7. Cronograma de execução

Metas e atividades	2019											
	Jan	Fev	Mar	Abr	Maio	Jun	Jul	Ago	Set	Out	Nov	Dez
Submissão do Projeto ao CEP				X	X							
Cadastro do projeto ao Registro Brasileiro Ensaios Clínicos					X	X						
Treinamento do avaliador com o equipamento da cinemática						X						
Recrutamento dos voluntários e coleta de dados						X	X	X	X	X	X	X
Processamento e análise dos dados											X	X
Atualização Bibliográfica					X	X	X	X	X	X	X	X
	**2020**											
Recrutamento dos voluntários e coleta de dados	X	X	X	X	X							
Processamento e análise dos dados						X	X	X				
Discussão e apresentação parcial dos resultados em eventos científicos			X	X	X	X	X	X	X	X	X	X
Atualização Bibliográfica	X	X	X	X	X	X	X	X	X	X	X	X
	**2021**											
Atualização Bibliográfica	X	X	X	X	X	X	X					
Elaboração, submissão, revisão e publicação dos manuscritos	X	X	X	X	X	X	X					
Defesa pública da Tese de Doutorado								X				
	**2022**											
Atualização Bibliográfica	X	X	X	X	X	X	X	X	X	X	X	X
	**2023**											
Atualização Bibliográfica	X	X	X	X	X	X	X	X	X	X	X	X
Recrutamento dos voluntários e coleta de dados – após aprovação da emenda pelo CEP	X	X	X	X	X	X	X	X	X			
Processamento e análise dos dados				X	X	X	X	X	X	X	X	X
Elaboração, submissão, revisão e publicação dos manuscritos								X	X	X	X	X
	**2024**											
Atualização Bibliográfica	X	X										
Elaboração, submissão, revisão e publicação dos manuscritos	X	X										
Defesa pública da Tese de Doutorado		X										

8. Orçamento detalhado e justificado

Itens de custeio		
10 pacotes de 100 eletrodos duplo descartáveis (Double Trace, Shanghai Litu Medical Appliances Co., Ltda, China)	Os eletrodos duplos de captura do sinal eletromiográfico (Double Trace da Shanghai Litu Medical Appliances Co., Ltda, China).	R$1.800,00
Material de papelaria, como folha de ofício A4, impressões, cópias, canetas esferográficas e cartuchos para impressora	Este material será utilizado para impressão das fichas de avaliação utilizadas na pesquisa e dos termos de consentimento livre e esclarecido.	R$ 400,00
**Total - custeio**		**R$2.200,00**

Referências

1. SERGIENKO S, KALICHMAN L. Myofascial origin of shoulder pain: A literature review. **J Bodyw Mov Ther.** v.19, n. 1, p. 91-101, 2015.

2. LIN JJ, HANTEN WP, OLSON SL, RODDEY TS, SOTO-QUIJANO DA, LIM HK, et al. Functional activity characteristics of individuals with shoulder dysfunctions. J **Electromyogr Kinesiol**. v. 15, n. 6, p. 576-86, 2005.

3. LEWIS J, MCCREESH K, ROY J-S, GINN K. Rotator Cuff Tendinopathy: Navigating the Diagnosis-Management Conundrum. **J Orthop Sport Phys Ther**. v. 45, n. 11, p. 923-37, 2015.

4. LUDEWIG PM, REYNOLDS JF. The Association of Scapular Kinematics and Glenohumeral Joint Pathologies. **J Orthop Sport Phys Ther**. v. 39, n. 2, p. 90–104, 2009.

5. SEITZ AL, MCCLURE PW, FINUCANE S, BOARDMAN ND, MICHENER LA. Mechanisms of rotator cuff tendinopathy: Intrinsic, extrinsic, or both? **Clin Biomech**. v. 26, n. 1, p. 1-12, 2011.

6. CAMARGO PR. Eccentric training as a new approach for rotator cuff tendinopathy: Review and perspectives. **World J Orthop**. v. 5, n. 5, p. 634, 2014.

7. LUDEWIG PM, REYNOLDS JF. The Association of scapulair Kinematics and Glenohumeral Joint Pathologies. **J Orthop Sport Phys Ther**. v. 39, n. 2, p. 90-104, 2009.

8. TIMMONS MK, THIGPEN CA, SEITZ AL, KARDUNA AR, ARNOLD BL, MICHENER LA. Scapular Kinematics and Subacromial-Impingement Syndrome : A Meta-Analysis. p. 354-70, 2012.

9. PADKE V, CAMARGO PR, LUDEWIG PM. Scapular and rotator cuff muscle activity during arm elevation: A review of normal function and alterations with shoulder impingement. **Rev Bras Fisioter**. v. 13, n. 1, p. 1-9, 2009.

10. PAGE P. Shoulder muscle imbalance and subacromial impingement syndrome in overhead athletes. **Int J Sports Phys Ther**. v. 6, n. 1, p. 51-8, 2011.

11. DIEDERICHSEN LP, NØRREGAARD J, DYHRE-POULSEN P, WINTHER A, TUFEKOVIC G, BANDHOLM T, et al. The activity pattern of shoulder muscles in subjects with and without subacromial impingement. **J Electromyogr Kinesiol**. v. 19, n. 5, p. 789-99, 2009.

12. HUNG CJ, JAN MH, LIN YF, WANG TQ, LIN JJ. Scapular kinematics and impairment features for classifying patients with subacromial impingement syndrome. **Man Ther**. v. 15, n. 6, p. 547-51, 2010.

13. MICHENER LA, SHARMA S, COOLS AM, TIMMONS MK. Relative scapular muscle activity ratios are altered in subacromial pain syndrome**. J Shoulder Elb Surg.** v. 25, n. 11, p. 1861-7, 2016.

14. MYERS JB, HWANG JH, PASQUALE MR, BLACKBURN JT, LEPHART SM. Rotator cuff coactivation ratios in participants with subacromial impingement syndrome. **J Sci Med Sport**. v. 12, n. 6, p. 603-8, 2009.

15. DYRNA F, KUMAR NS, OBOPILWE E, SCHEIDERER B, COMER B, NOWAK M, et al. Relationship Between Deltoid and Rotator Cuff Muscles During Dynamic Shoulder Abduction: A Biomechanical Study of Rotator Cuff Tear Progression. **Am J Sports Med**. v. 46, n. 8, p. 1919-26, 2018.

16. MULLA DM, MCDONALD AC, KEIR PJ. Upper body kinematic and muscular variability in response to targeted rotator cuff fatigue. **Hum Mov Sci**. v. 59, n. 6, p. 121-33, 2018.

17. PADKE V, CAMARGO PR, LUDEWIG PM. Scapular and rotator cuff muscle activity during arm elevation: A review of normal function and alterations with shoulder impingement. **Rev Bras Fisioter**. v. 13, n. 1, p. 1-9, 2009.

18. TOLIOPOULOS P, DESMEULES F, BOUDREAULT J, ROY JS, FRÉMONT P, MACDERMID JC, et al. Efficacy of surgery for rotator cuff tendinopathy: a systematic review. **Clin Rheumatol**. 2014;33(10):1373–83.

19. WANG JHC, IOSIFIDIS MI, FU FH. Biomechanical basis for tendinopathy. Clin Orthop Relat Res. v. 44, n. 3, p. 320-32, 2006.

20. KADER D. Achilles tendinopathy: some aspects of basic science and clinical management. **Br J Sport Med**. v. 36, n.1, p. 239-49, 2002.

21. KUHN JE. Exercise in the treatment of rotator cuff impingement: A systematic review and a synthesized evidence-based rehabilitation protocol**. J Shoulder Elb Surg**. v. 18, n. 1, p. 138-60, 2009.

22. BLUME C, WANG-PRICE S, TRUDELLE-JACKSON E, ORTIZ A. Comparison of Eccentric and Concentric Exercise Interventions in Adults With Subacromial Impingement Syndrome. **Int J Sports Phys Ther.** v. 10, n. 4, p. 441-55, 2015.

23. LEWIS J. Rotator cuff related shoulder pain: Assessment, management and uncertainties. **Man Ther**. v. 23, p. 57-68, 2016.

24. KONGSGAARD M, KOVANEN V, AAGAARD P, DOESSING S, HANSEN P, LAURSEN AH, et al. Corticosteroid injections, eccentric decline squat training and heavy slow resistance training in patellar tendinopathy. **Scand J Med Sci Sport**. v. 19, n. 6, p. 790-802, 2009.

25. BEYER R, KONGSGAARD M, HOUGS KJÆR B, ØHLENSCHLÆGER T, KJÆR M, MAGNUSSON SP. Heavy slow resistance versus eccentric training as treatment for achilles tendinopathy: A randomized controlled trial. **Am J Sports Med**. v. 43, n. 7, p. 1704-11, 2015.

26. PEARSON SJ, STADLER S, MENZ H, MORRISSEY D, SCOTT I, MUNTEANU S, et al. Immediate and Short- Term Effects of Short-and Long-Duration Isometric Contractions in Patellar Tendinopathy. v. 00, n. 00, p. 1-6, 2018.

27. RIO E, KIDGELL D, PURDAM C, GAIDA J, MOSELEY GL, PEARCE AJ, et al. Isometric exercise induces analgesia and reduces inhibition in patellar tendinopathy. **Br J Sports Med.** v. 49, n. 19, p. 1277-83, 2015.

28. RIO E, HONS BAP, PHYS M, ARK M VAN, DOCKING S, HONS B, et al. Isometric Contractions Are More Analgesic Than Isotonic Contractions for Patellar Tendon Pain : An In-Season Randomized Clinical Trial. v. 0, n. 0, p. 1-7, 2016.

29. NAUGLE KM, NAUGLE KE, FILLINGIM RB, RILEY JL. Isometric Exercise as a Test of Pain Modulation: Effects of Experimental Pain Test, Psychological Variables, and Sex. **Pain Med**. v. 15, n. 4, p. 692-701, 2014.

30. PARLE PJ, RIDDIFORD-HARLAND DL, HOWITT CD, LEWIS JS. Acute rotator cuff tendinopathy: Does ice, low load isometric exercise, or a combination of the two produce an analgaesic effect? **Br J Sports Med**. v. 51, n. 3, p. 208-9, 2017.

31. MINAGAWA H, YAMAMOTO N, ABE H, FUKUDA M, SEKI N, KIKUCHI K, et al. Prevalence of symptomatic and asymptomatic rotator cuff tears in the general population: From mass- screening in one village. **J Orthop**. v. 10, n. 1, p. 8-12, 2013

32. TASHJIAN RZ. Epidemiology, Natural History, and Indications for Treatment of Rotator Cuff Tears. **Clin Sports Med**. v. 31, n. 4, p. 589-604, 2012.

33. MACDERMID JC, SILBERNAGEL KG. Outcome Evaluation in Tendinopathy: Foundations of Assessment and a Summary of Selected Measures. **J Orthop Sport Phys Ther.** v. 45, n. 11, p. 950-64, 2015.

34. CHIEN CW, BAGRAITH KS, KHAN A, DEEN M, STRONG J. Comparative responsiveness of verbal and numerical rating scales to measure pain intensity in patients with chronic pain. **J Pain**. v. 14, n. 12, p. 1653–62, 2013.

35. COOLS AM, CAMBIER D, WITVROUW EE. Screening the athlete's shoulder for impingement symptoms: A clinical reasoning algorithm for early detection of shoulder pathology. **Br J Sports Med**. v. 42, n. 8, p. 628-35, 2008.

36. MICHENER LA, WALSWORTH MK, DOUKAS WC, MURPHY KP. Reliability and Diagnostic Accuracy of Physical Examination Tests and Combination of Tests for Subacromial Impingement. **Arch Phys Med Rehabil**. v, 90, n. 11, p. 1898-903, 2009.

37. NAREDO E. Painful shoulder: comparison of physical examination and ultrasonographic findings. Ann Rheum Dis. v. 61, n. 2, p. 132-6, 2002.

38. WALMSLEY S, RIVETT DA, OSMOTHERLY PG. Adhesive capsulitis: establishing consensus on clinical identifiers for stage 1 using the DELPHI technique. **Phys Ther**. v. 89, n. 9, p. 906-17, 2009.

39. ALBURQUERQUE-SENDÍN F, CAMARGO P, VIEIRA A, SALVINI T. Bilateral myofascial trigger points and pressure pain thresholds in the shoulder muscles in patients with unilateral shoulder impingement syndrome: a blinded, controlled study. **Clin J Pain**. v. 29, n. 6, p. 478-86, 2013.

40. MCCLURE PW, MICHENER L A, KARDUNA AR. Shoulder function and 3-dimensional scapular kinematics in people with and without shoulder impingement syndrome. **Phys Ther**. v. 86, n. 8, p. 1075-90, 2006.

41. SANTAMATO A, SOLFRIZZI V, PANZA F, TONDI G, FRISARDI V, LEGGIN BG, et al. Short-term effects of high-intensity laser therapy versus ultrasound therapy in the treatment of people with subacromial impingement syndrome: a randomized clinical trial. **Phys Ther**. v. 89, n. 7, p. 643-52, 2009.

42. LEWIS T, COOK J. Fluoroquinolones and tendinopathy: A guide for athletes and sports clinicians and a systematic review of the literature. **J Athl Train.** v. 49, n. 3, p.422–7, 2014.

43. LUI PPY. Tendinopathy in diabetes mellitus patients—Epidemiology, pathogenesis, and management. **Scand J Med Sci Sport.** V. 27, n. 8, p. 776–87, 2017.

44. Napoles BV, Hoffman CB, Martins J, Oliveira AS De. Tradução e adaptação cultural do Penn Shoulder Score para a Língua Portuguesa: PSS-Brasil. Rev Bras Reumatol. v. 50, n. 4, p. 389-97, 2010.

45. LOPES AD, CICONELLI RM, CARRERA EF, GRIFFIN S, FALOPPA F, DOS REIS FB. Validity and reliability of the Western Ontario Rotator Cuff Index (WORC) for use in Brazil. **Clin J Sport Med**. v. 18, n. 3, p. 266-72, 2008.

46. Michener LA, Elmore KA, Darter BJ, Timmons MK. Biomechanical measures in participants with shoulder pain: Intra-rater reliability. Man Ther. 2016; 22:86–93.

47. HERMENS HJ, FRERIKS B, MERLETTI R, ET AL. Project SENIAM (Surface Electromyography for the Non-Invasive Assessment of Muscles). European Recommendations for Surface Electromyography. 1999.

48. SOUSA C DE O, MICHENER LA, RIBEIRO IL, REIFF RB DE M, CAMARGO PR, SALVINI TF. Motion of the shoulder complex in individuals with isolated acromioclavicular osteoarthritis and associated with rotator cuff dysfunction: Part 2 - Muscle activity. **J Electromyogr Kinesiol**. v. 25, n. 1, p. 77-83, 2015.

49. PUGA VODO, LOPES AD, SHIWA SR, ALOUCHE SR, COSTA LOP. Clinimetric Testing Supports the Use of 5 Questionnaires Adapted Into Brazilian Portuguese for Patients With Shoulder Disorders. **J Orthop Sport Phys Ther**. v. 43, n. 6, p. 404-13, 2013.

50. LUDEWIG PM, PHADKE V, BRAMAN JP, HASSETT DR, CIEMINSKI CJ, LAPRADE RF. Motion of the shoulder complex during multiplanar humeral elevation. **J Bone Jt Surg**. v. 91, n. 2, p. 378-89, 2009.

51. WU G, VAN DER HELM FCT, VEEGER HEJ, MAKHSOUS M, VAN ROY P, ANGLIN C, et al. ISB recommendation on definitions of joint coordinate systems of various joints for the reporting of human joint motion - Part II: Shoulder, elbow, wrist and hand. **J Biomech**. v. 38, n. 5, p. 981-92, 2005.

52. TATE AR, MCCLURE P, KAREHA S, IRWIN D, BARBE MF. A clinical method for identifying scapular dyskinesis, part 2: Validity**. J Athl Train.** v. 44, n. 2, p. 165-73, 2009.

53. CAMARGO PR, ALBURQUERQUE-SENDÍN F, AVILA MA, HAIK MN, VIEIRA A, SALVINI TF. Effects of Stretching and Strengthening Exercises, With and Without Manual Therapy, on Scapular Kinematics, Function, and Pain in Individuals With Shoulder Impingement: A Randomized Controlled Trial. **J Orthop Sport Phys Ther**. v. 45, n. 12, p. 984-97, 2015.

54. SALAMH PA, KOLBER MJ, HANNEY WJ. Effect of scapular stabilization during horizontal adduction stretching on passive internal rotation and posterior shoulder tightness in young women volleyball athletes: A randomized controlled trial. **Arch Phys Med Rehabil**. v. 96, n. 2, p. 349-56, 2015.

55. LAWRENCE RL, SCHLANGEN DM, SCHNEIDER KA, SCHOENECKER J,

SENGER AL, STARR WC, et al. Effect of glenohumeral elevation on subacromial supraspinatus compression risk during simulated reaching. **J Orthop Res.** v. 35, n. 10, p. 2329-37, 2017.

56. REINOLD MM, MACRINA LC, WILK KE, FLEISIG GS, DUN S, BARRENTINE SW, et al. Electromyographic analysis of the supraspinatus and deltoid muscles during 3 common rehabilitation exercises. **J Athl Train.** v. 42, n. 4, p. 464-9, 2007.

57. COOLS AMJ, VANDERSTUKKEN F, VEREECKEN F, DUPREZ M, HEYMAN K, GOETHALS N, et al. Eccentric and isometric shoulder rotator cuff strength testing using a hand-held dynamometer: reference values for overhead athletes. **Knee Surgery, Sport Traumatol Arthrosc.** v. 24, n. 12, p. 3838-47, 2016.

ANEXOS

Anexo I. *Penn Shoulder Score* (PSS)

IDENTIFICAÇÃO DO PACIENTE

Nome completo: . . . . Registro: . . . .

Data de nascimento: . . . . / . . . . / . . . . Idade: . . . . Sexo: ( ) F ( ) M

Profissão: . . . . Tel: . . . . Dominância: ( ) D ( ) E

Hipótese diagnóstica: . . . .

Cirurgia: Qual: . . . . Quando: . . . . / . . . . / . . . .

Braço com dor ou disfunção: ( ) D ( ) E ( ) Ambos Pior braço: ( ) D ( ) E

Há quanto tempo tem dor ou disfunção nesse braço: . . . .

PENN SHOULDER SCORE (PSS-BRASIL)

Nome: . . . . Braço avaliado: . . . . Data: . . . . . / . . . . . / . . . . .

PONTUAÇÃO PSS-BRASIL PARA O OMBRO

Parte I: Dor e Satisfação: Por favor, indique o número que mais se aproxima do seu nível de dor ou satisfação.

											**Uso Exclusivo**
Dor em repouso, com o braço parado ao lado do corpo:											
0	1	2	3	4	5	6	7	8	9	10	. . . .
sem dor										pior dor possível	(10 – Nº circulado)
Dor durante atividades normais (comer, vestir-se, banhar-se):											
0	1	2	3	4	5	6	7	8	9	10	. . . . (10 – Nº circulado)
sem dor										pior dor possível	(marcar 0 se não se aplica)
Dor durante atividades de esforço (alcançar, levantar, empurrar, puxar, jogar um objeto):											
0	1	2	3	4	5	6	7	8	9	10	. . . . (10 – Nº circulado)
sem dor										pior dor possível	(marcar 0 se não se aplica)
										**Pontuação para dor =**	. . . . / 30
Qual a sua satisfação com o nível atual de função do seu ombro?											
0	1	2	3	4	5	6	7	8	9	10	. . . . / 10
não satisfeito										muito satisfeito	(Nº circulado)

PONTUAÇÃO PSS-BRASIL

Parte II: Função: Por favor, indique o nível de dificuldade que você pode ter ao realizar cada atividade.

		Já não realizava antes da lesão	Sem dificuldade	Alguma dificuldade	Muita dificuldade	Não consegue de forma alguma
1	Alcançar a parte inferior da sua coluna com a mão do braço afetado, para arrumar a camisa dentro das calças.	X	3	2	1	0
2	Lavar o meio das costas ou prender o sutiã pelas costas com o braço afetado.	X	3	2	1	0
3	Realizar atividades de higiene pessoal com o braço afetado.	X	3	2	1	0
4	Lavar a parte posterior do ombro oposto com o braço afetado.	X	3	2	1	0
5	Pentear os cabelos com o braço afetado.	X	3	2	1	0
6	Colocar a mão do braço afetado atrás da cabeça com o cotovelo para fora e para o lado.	X	3	2	1	0
7	Vestir-se (incluindo vestir casaco e tirar a blusa pela cabeça).	X	3	2	1	0
8	Dormir em cima do lado afetado.	X	3	2	1	0
9	Abrir/empurrar a porta com o braço afetado.	X	3	2	1	0
10	Carregar um livro ou pasta, junto ao corpo, com o braço afetado.	X	3	2	1	0
11	Carregar uma sacola de compras ou maleta com o braço afetado.	X	3	2	1	0
12	Colocar uma lata (500 g a 1 kg) em uma prateleira à altura do ombro com o braço afetado esticado.	X	3	2	1	0
13	Colocar um pote de aproximadamente 5 kg (saco grande de arroz) em uma prateleira à altura do ombro com o braço afetado esticado.	X	3	2	1	0
14	Alcançar uma prateleira acima da cabeça com o braço afetado esticado.	X	3	2	1	0
15	Colocar uma lata (500 g a 1 kg) em uma prateleira acima da cabeça com o braço afetado esticado.	X	3	2	1	0
16	Colocar um pote de aproximadamente 5 kg (saco grande de arroz) em uma prateleira acima da cabeça com o braço afetado esticado.	X	3	2	1	0
17	Praticar atividades de lazer regulares ou esportes.	X	3	2	1	0
18	Realizar as tarefas de casa (limpar, lavar a roupa, cozinhar).	X	3	2	1	0
19	Arremessar acima do ombro/ nadar/ esportes com raquete, com o braço afetado. (Circule as atividades que se aplicam ao paciente)	X	3	2	1	0
20	Trabalhar o tempo todo em seu emprego ou função usual.	X	3	2	1	0

PONTUAÇÃO PARA FUNÇÃO

Total de colunas = ... (a)

Número de "X" x 3 = ... (b), 60 - . . . . (b) = ... (c)

(se nenhum X for circulado, a pontuação da função = número total de colunas)

Pontuação da função = ... (a) ÷ . . . . (c) = ... x 60 . . . . /60

Pontuação total (Parte I e II) =

Anexo II. ***Western Ontario Rotator Cuff Index*** **(WORC)**

INSTRUÇÕES AOS PACIENTES

As perguntas deste questionário possuem o formato abaixo. Você deverá indicar sua resposta colocando uma barra "/" na linha horizontal de acordo com a explicação seguinte:

OBSERVE:

1. Se você colocar uma barra "/" à esquerda, no final da linha, isto é:

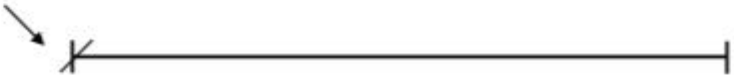

Então, você está indicando que não tem dor.

2. Se você colocar uma barra "/" à direita, no final da linha, isto é:

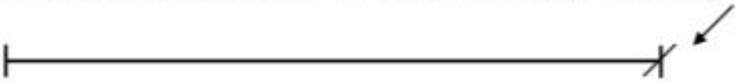

Então, você está indicando que sua dor é extrema.

Por favor, preste atenção:

a) quanto mais à direita você colocar a barra "/", **mais** você apresentará aquele sintoma.

b) quanto mais à esquerda você colocar a barra "/", **menos** você apresentará aquele sintoma.

c) Não coloque sua barra "/" fora dos marcadores finais.

Você deverá indicar no questionário a intensidade do sintoma que você sentiu nesta última semana com relação a seu ombro afetado. Se você não tiver certeza sobre o ombro que está envolvido ou se você tiver quaisquer outras dúvidas, pergunte antes de preencher o questionário.

Se, por algum motivo, você não entender uma pergunta, procure as explicações que estão incluídas no final desse questionário.

Se um item não se relacionar a você ou se você não o tiver sentido nesta última semana, imagine qual seria sua resposta mais adequada para tal.

Seção A: Sintomas Físicos
INSTRUÇÕES AOS PACIENTES

As perguntas abaixo relacionam aos sintomas físicos que você apresentou por causa do problema do seu ombro. Em todos os casos, por favor, indique a quantidade de sintomas que você apresentou nesta última semana. (Por favor, assinale a sua resposta com uma barra "/").

1. Quanta dor aguda você sente no seu ombro?

dor
sem dor |————————————| extrema

2. Quanta dor constante, incômoda, você sente no seu ombro?

sem dor
dor |————————————| extrema

3. Quanta fraqueza você sente no seu ombro ?

sem
fraqueza |————————————| fraqueza extrema

4. Quanto você sente seu ombro endurecido ou travado?

Nada |————————————| extremamente
endurecido

5. Quanto você se sente incomodado quando seu ombro estala, range ou crepita?

nenhum |————————————| extremamente
incomodado
incomodo

6. Quanto desconforto você sente nos músculos do seu pescoço por causa do seu ombro?

sem |————————————| extremo desconforto
desconforto

Seção B: Esportes/Recreação
INSTRUÇÕES AOS PACIENTES

A seção a seguir questiona como o problema do seu ombro afetou suas atividades esportivas ou de lazer nesta última semana. (Por favor, para cada pergunta assinale sua resposta através de uma barra "/").

7. Quanto o seu ombro afetou seu nível de desempenho físico?

não ├──────────┤ afetou
extremamente
afetado

8. Quanto o seu ombro afetou sua habilidade de arremessar com força ou à distância?

extremamente
não
afetou ├──────────┤ afetado

9. Quanto medo você tem de que alguém ou alguma coisa esbarre no seu ombro afetado?

nenhum ├──────────┤ medo extremo

10. Quanta dificuldade você sente quando faz "flexão de braços" ou outros exercícios pesados por causa do seu ombro?

extrema
sem
dificuldade ├──────────┤ dificuldade

Seção C: Trabalho
INSTRUÇÕES AOS PACIENTES

A seção abaixo questiona quanto o problema do seu ombro afetou o seu trabalho em casa e fora de casa. (Por favor, indique a quantidade apropriada nesta última semana com uma barra "/").

11. Quanta dificuldade você sente na execução das atividades diárias em casa ou nas áreas externas dela (ex: jardim, quintal)?

nenhuma

extrema

dificuldade dificuldade

12. Quanta dificuldade você sente para desempenhar tarefas acima do nível de sua cabeça?

nenhuma dificuldade — extrema dificuldade

13. Quanto você usa seu braço bom para substituir seu braço machucado?

Não uso — constantemente

14. Quanta dificuldade você tem para levantar objetos pesados na altura ou abaixo da altura do seu ombro?

sem dificuldade — extrema dificuldade

Seção D: Estilo de vida
INSTRUÇÕES AOS PACIENTES

A seção seguinte questiona quanto o seu problema do ombro afetou ou mudou seu estilo de vida (Novamente, indique a quantidade apropriada nesta última semana com uma barra "/").

15. Quanta dificuldade você tem para dormir por causa do seu ombro?

Sem dificuldade |———————————| extrema dificuldade

16. Quanto desconforto você sente para arrumar o seu cabelo por causa do seu ombro?

nenhum desconforto |———————————| extremo desconforto

17. Quanta dificuldade você tem para brincar/rolar no chão com familiares ou amigos?

nenhuma dificuldade |———————————| extrema

dificuldade

18. Quanta dificuldade você tem para se vestir ou se despir?

Sem dificuldade |———————————| extrema

dificuldade

Seção E: Emoções
INSTRUÇÕES AOS PACIENTES

As perguntas abaixo questionam como você se sentiu nesta última semana com relação ao problema do seu ombro (Por favor, indique sua resposta com uma barra "/").

19. Quanta frustração você sente por causa do seu ombro?

nenhuma frustração |————————————————| extrema frustração

20. Quanto você se sente triste ou deprimido por causa do seu ombro?

nada extremamente |————————————————|

21. Quanto você se sente preocupado com relação aos efeitos do seu ombro na sua ocupação ou trabalho?

não me sinto extremamente preocupado preocupado |————————————————|

OBRIGADO POR COMPLETAR O QUESTIONÁRIO

APÊNDICES

Apêndice A. Cartaz de Divulgação

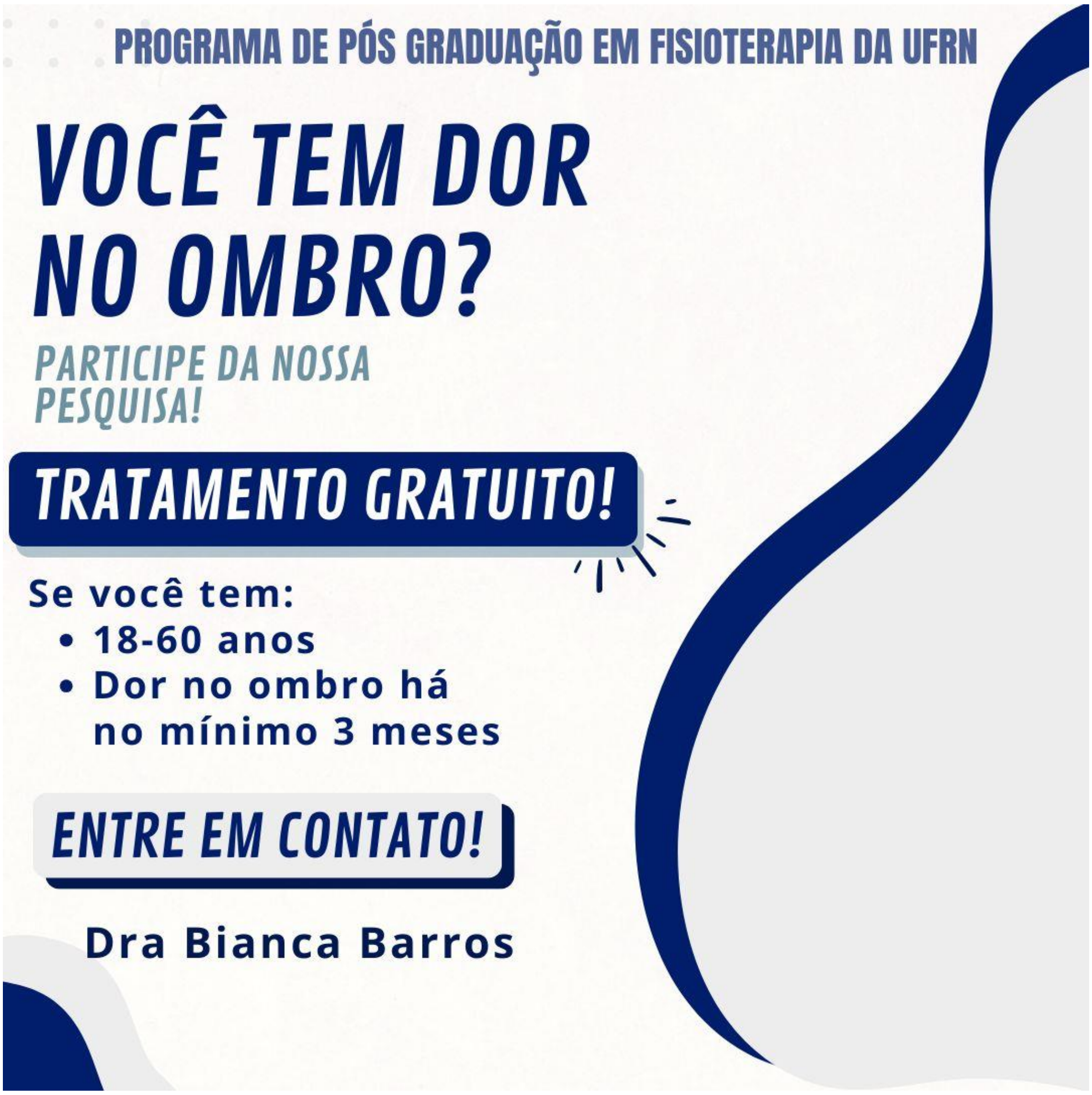

Apêndice B. Termo de Consentimento Livre e Esclarecido

UNIVERSIDADE FEDERAL DO RIO GRANDE DO NORTE
CENTRO DE CIÊNCIAS DA SAÚDE
DEPARTAMENTO DE FISIOTERAPIA

TERMO DE CONSENTIMENTO LIVRE E ESCLARECIDO – TCLE

Este é um convite para você participar da pesquisa: “Exercício isométrico *versus* isotônico na tendinopatia do manguito rotador – efeitos na dor, função e controle neuromuscular: um ensaio clínico randomizado”, desenvolvida pela doutoranda em Fisioterapia Bianca Rodrigues da Silva Barros e tem como pesquisador responsável a Professora Catarina de Oliveira Sousa.

Esta pesquisa pretende comparar e caracterizar dois tipos de exercícios de fortalecimento de músculos do ombro na dor, função e controle do ombro em pessoas com tendinopatia do manguito rotador, grupo muscular responsável por estabilizar o ombro.

O motivo que nos leva a fazer este estudo é entender melhor os efeitos e as características dos exercícios de fortalecimento utilizados no tratamento de disfunções do ombro. O tratamento conservador, baseado em intervenção fisioterapêutica, é indicado para o tratamento das tendinopatias e rupturas dos músculos do manguito rotador, especialmente o treinamento resistido que impõe carga ao tendão de forma progressiva, a fim de auxiliar na sua reparação por meio da alteração de seu metabolismo e propriedades mecânicas e estruturais. Dentre as diversas formas de fortalecimento, os exercícios excêntricos e concêntricos (que são realizados com o movimento do ombro) têm se mostrado eficazes para a melhora da função geral do ombro, e poucos estudos têm sido desenvolvidos avaliando os efeitos do exercício isométrico (realizados sem movimento do ombro) na tendinopatia do MR.

Caso você tenha tendinopatia do manguito rotador diagnosticada por um cirurgião ortopedista por meio de exames de imagem do tipo ultrasom ou ressonância nuclear magnética e decida aceitar o convite, você será submetido

a alguns procedimentos de avaliação e de intervenção. Os procedimentos de avaliação ocorrerão em três momentos e você terá que comparecer em 03 dias distintos. Já os procedimentos da intervenção serão realizados duas vezes por semana por um periodo total de 06 semanas. Sua participação não é obrigatória e você poderá se retirar da pesquisa em qualquer momento, caso queira. No primeiro momento da avaliação você será examinado por uma fisioterapeuta com 6 anos de experiência. Essa avaliação constará de coleta de dados pessoais e história clínica, e exame físico para verificar se você tem sinais e sintomas de tendinopatia em um dos músculos do manguito rotador e excluir outras condições clínicas dolorosas do ombro.

Após a conclusão dessa avaliação incial, você passará por uma avaliação para determinação da carga e familiarização dos exercícios que serão realizados no período da intervenção. Entre 3 e 7 dias, serão realizadas as avaliações de dor, função, atividade elétrica dos músculos e análise dos movimentos do ombro. Essas avaliações se repetirão em outros dois momentos: exatamente após a primeira sessão de treinamento com exercícios e após o período total de treinamento, ao final de 06 semanas. Para a avaliação da atividade elétrica dos músculos e do movimento, serão colocados sensores afixados com adesivos do tipo dupla-face e esparadrapo hipoalergênico em pontos anatômicos específicos do ombro e você será orientado a realizar alguns movimentos de elevação do braço. Esses sensores não geram nenhuma estimulação ou sensação dolorosa.

Após estes procedimentos de avaliação você participará de um programa de treinamento de 06 semanas, de acordo com o grupo que você for alocado por sorteio: 1) exercícios envolvendo alongamentos da musculatura do pescoço e ombro, fortalecimento dos músculos que movimentam a escápula e fortalecimento dos músculos do manguito rotador de forma isométrica; ou 2) exercícios envolvendo alongamentos da musculatura do pescoço e ombro, fortalecimento dos músculos que movimentam a escápula e fortalecimento dos músculos do manguito rotador de forma isotônica.

Todos os procedimentos, desde as avaliações até o programa de intervenção serão realizados no Laboratório de Análise da Performance Neuromuscular no Departamento de Fisioterapia da Universidade Federal do Rio Grande do Norte (UFRN), garantindo total privacidade para os participantes do

estudo.

As avaliações e intervenções não têm caráter invasivo, no entanto durante a realização da pesquisa poderão ocorrer eventuais desconfortos musculares em decorrência da realização dos exercícios físicos, que poderão ser minimizados com aplicação de crioterapia para reduzir esses desconfortos.

Como benefícios da pesquisa você terá uma avaliação completa do seu ombro e receberá um programa de treinamento com o objetivo de reduzir a dor e melhorar a função e o controle motor de seu ombro. Caso observemos ao final da pesquisa uma superioridade nos resultados de um grupo em detrimento de outro, garantiremos aos participantes aquele protocolo de tratamento com os melhores resultados.

Em caso de algum problema que você possa ter relacionado(a) com a pesquisa, você terá direito à assistência gratuita que será prestada pelos responsáveis da pesquisa no serviço de Fisioterapia da UFRN.

Durante todo o período da pesquisa você poderá tirar suas dúvidas ligando para a Professora Catarina de Oliveira Sousa (pesquisadora responsável).

Você tem o direito de se recusar a participar ou retirar seu consentimento, em qualquer fase da pesquisa, sem nenhum prejuízo para você.

Os dados que você irá nos fornecer serão confidenciais e serão divulgados apenas em congressos ou publicações científicas, sempre de forma anônima, não havendo divulgação de nenhum dado que possa lhe identificar. Esses dados serão guardados pela pesquisadora responsável por essa pesquisa em local seguro e por um período de 5 anos.

Qualquer eventual gasto realizado pela sua participação nessa pesquisa, eles serão assumidos pela pesquisadora responsãvel e reembolsado para você.

Se você sofrer qualquer dano decorrente desta pesquisa, sendo ele imediato ou tardio, previsto ou não, você será indenizado.

Qualquer dúvida sobre a ética dessa pesquisa você deverá ligar para o Comitê de Ética em Pesquisa – instituição que avalia a ética das pesquisas antes que elas comecem e fornece proteção aos participantes das mesmas – da Universidade Federal do Rio Grande do Norte. Você ainda pode ir pessoalmente à sede do CEP, de segunda a sexta, das 08:00h às 12:00h e das 14:00h às

18:00h, na Universidade Federal do Rio Grande do Norte, Av. Senador Salgado Filho, s/n. Campus Central, Lagoa Nova. Natal/RN.

Este documento foi impresso em duas vias. Uma ficará com você e a outra com a pesquisadora responsável Profa. Catarina de Oliveira Sousa.

Consentimento Livre e Esclarecido

Após ter sido esclarecido sobre os objetivos, importância e o modo como os dados serão coletados nessa pesquisa, além de conhecer os riscos, desconfortos e benefícios que ela trará para mim e ter ficado ciente de todos os meus direitos, concordo em participar da pesquisa “Exercício isométrico versus istotônico na tendinopatia do manguito rotador – efeitos na dor, função e controle neuromuscular: um ensaio clínico randomizado”, e autorizo a divulgação das informações por mim fornecidas em congressos e/ou publicações científicas desde que nenhum dado possa me identificar.

Natal (RN), / / .

Impressão datiloscópica do participante

Assinatura do participante da pesquisa

Declaração da pesquisadora responsável

Como pesquisadora responsável pelo estudo “Exercício isométrico versus istotônico na tendinopatia do manguito rotador – efeitos na dor, função e controle neuromuscular: um ensaio clínico randomizado”, declaro que assumo a inteira responsabilidade de cumprir fielmente os procedimentos metodologicamente e direitos que foram esclarecidos e assegurados ao participante desse estudo, assim como manter sigilo e confidencialidade sobre a identidade do mesmo.

Declaro ainda estar ciente que na inobservância do compromisso ora assumido estarei infringindo as normas e diretrizes propostas pela Resolução 466/12 do Conselho Nacional de Saúde – CNS, que regulamenta as pesquisas envolvendo o ser humano.

Natal _____/_____/_______.

____

Profa. Dra. Catarina de Oliveira Sousa

Pesquisadora responsável

Apêndice C. Ficha de Avaliação Individual

FICHA DE AVALIAÇÃO

Nº de Identificação: ___________________________ Telefone: ( ) ___________
Data de triagem _____/______/_____
Idade ______anos Data de Nascimento ____/____/_____ Profissão _______________
Sexo ○ Masculino ○Feminino
Raça ○Branca ○Negra ○Parda ○Indígena ○Amarela

Você é capaz de ler e compreender português? ○ Sim○ Não

HISTÓRIA CLÍNICA
Sente dor no ombro: ○ Sim ○Não ○ Direito ○ Esquerdo ○ Ambos
Início dos Sintomas:
Já realizou tratamento anteriormente? ○ Sim ○Não Qual:
Faz uso de algum medicamento? ○ Sim ○Não Qual:
História de trauma no braço? ○ Sim ○Não Quando? O que?
Histórico de doença sistêmica de tecidos conectivos, ortopédicos ou neurológicos? ○ Sim ○Não Qual:
Possui capsulite adesiva? ○ Sim ○Não
Histórico de cirurgia nos MMSS? ○ Sim ○Não
EXAME FÍSICO
Dor à palpação dos tendões do MR? ○ Sim ○Não Qual:
Amplitude do arco doloroso na elevação do braço:<br><br>Ativa: ○ Sim ○Não<br><br>Resistida: ○ Sim ○Não
Testes Especiais:<br><br>Teste de Jobe: ○ Sim ○Não<br><br>Rotação externa resistida: ○ Sim ○Não<br><br>Teste de apreensão e recolocação: ○ Sim ○Não
DADOS ANTROPOMÉTRICOS / ATIVIDADE FÍSICA
Membro superior dominante? ○ Direito ○ Esquerdo ○ Ambos
Peso: ______ Altura:_______ IMC:______
Faz atividade física? ○ Sim ○Não. Quanto tempo? ___________<br>Tipo de atividade? ____________________<br>Frequência:○2x sem○ 3x sem ○ 5x sem
